# The Astounding World of Glycans from Giant Viruses

**DOI:** 10.1021/acs.chemrev.2c00118

**Published:** 2022-07-12

**Authors:** Immacolata Speciale, Anna Notaro, Chantal Abergel, Rosa Lanzetta, Todd L. Lowary, Antonio Molinaro, Michela Tonetti, James L. Van Etten, Cristina De Castro

**Affiliations:** † Department of Agricultural Sciences, University of Napoli, Via Università 100, 80055 Portici, Italy; ‡ Centre National de la Recherche Scientifique, Information Génomique & Structurale, Aix-Marseille University, Unité Mixte de Recherche 7256, IMM, IM2B, 13288 Marseille, Cedex 9, France; § Department of Chemical Sciences, University of Napoli, Via Cintia 4, 80126 Napoli, Italy; ∥ Institute of Biological Chemistry, Academia Sinica, Academia Road, Section 2, Nangang 11529, Taipei, Taiwan; ⊥ Department of Experimental Medicine and Center of Excellence for Biomedical Research, University of Genova, 16132 Genova, Italy; # Nebraska Center for Virology, University of Nebraska, Lincoln, Nebraska 68583-0900, United States; ∇ Department of Plant Pathology, University of Nebraska, Lincoln, Nebraska 68583-0722, United States

## Abstract

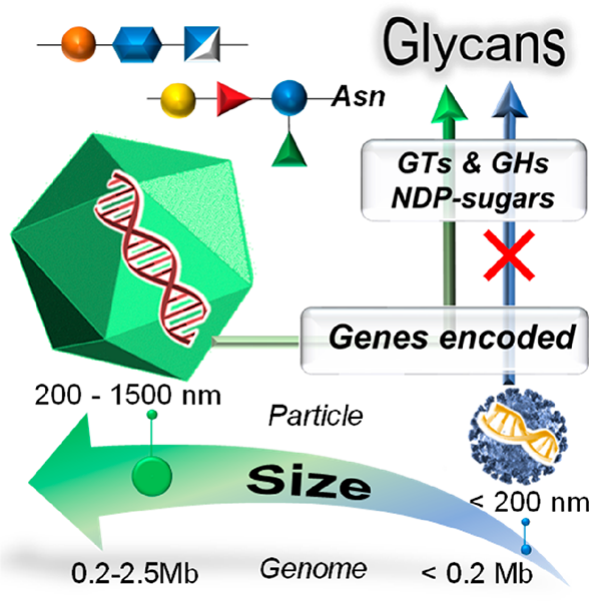

Viruses are a heterogeneous ensemble of entities, all sharing the need for a suitable host to replicate. They are extremely diverse, varying in morphology, size, nature, and complexity of their genomic content. Typically, viruses use host-encoded glycosyltransferases and glycosidases to add and remove sugar residues from their glycoproteins. Thus, the structure of the glycans on the viral proteins have, to date, typically been considered to mimick those of the host. However, the more recently discovered large and giant viruses differ from this paradigm. At least some of these viruses code for an (almost) autonomous glycosylation pathway. These viral genes include those that encode the production of activated sugars, glycosyltransferases, and other enzymes able to manipulate sugars at various levels. This review focuses on large and giant viruses that produce carbohydrate-processing enzymes. A brief description of those harboring these features at the genomic level will be discussed, followed by the achievements reached with regard to the elucidation of the glycan structures, the activity of the proteins able to manipulate sugars, and the organic synthesis of some of these virus-encoded glycans. During this progression, we will also comment on many of the challenging questions on this subject that remain to be addressed.

## 1 Introduction

The general perception of viruses is that they are small entities and dangerous human pathogens. This view is understandable because, over the years, viruses have caused major disease burdens such as AIDS and the Ebola outbreak in 2014 or the ongoing pandemic caused by SARS-CoV-2. However, this view is biased because it only focuses on a restricted number of viruses and it neglects the fact that viruses target all cellular organisms. Indeed, viruses are the most abundant biological entities on the planet, with surface ocean water reported to contain ∼10^7^ virus particles per milliliter.^1,2^ This value translates into ∼10^31^ virus particles in the world’s oceans, and this estimate does not include RNA viruses or viruses present in soils and other environments. Most of these viruses infect bacteria, with lesser numbers infecting microalgae and other organisms. Thus, viruses should be seen as integral components of any ecosystem where they contribute to the maintenance of the balance between species and resources, thus playing a positive role in the living community.^3^

Viruses are a heterogeneous ensemble of biological entities, all sharing the common trait that their replication requires a suitable host even though they vary in morphology, size, nature, and complexity of their genomic content. Depending on the virus, the genetic material can be either DNA or RNA, which exists either as double stranded (ds) or single stranded (ss) molecules while their physical size can vary by orders of magnitude. Viruses can be as small as 17 nm, e.g., porcine circovirus, which has a ssDNA genome of ∼1.8 kilobases (kb) that encodes for three proteins,^4^ or they can reach 1500 nm in size like Pithovirus sibericum,^5^ or they can be as genetically complex as Pandoravirus salinus, the virus with the largest genome found to date, with a dsDNA genome of ∼2500 kb, which encodes ∼1500 predicted proteins.^6^ Finally, viral particles (virions) can have different morphologies: e.g., porcine circovirus particles have an icosahedral geometry while Pandoravirus salinus, visible under a light microscope as are bacteria, has amphora-shaped virions that resemble the morphology of some bacteria. In contrast, many plant viruses are rod shaped.^7^

When discussing species that produce carbohydrate-manipulating enzymes, viruses are seldom considered as they are often thought to lack these proteins. Again, this view is flawed from the knowledge acquired from viruses that are human pathogens, such as rhabdoviruses, herpesviruses, poxviruses, coronaviruses, and paramyxoviruses, all endowed with structural proteins that are glycosylated. These human pathogenic viruses use host-encoded glycosyltransferases (GTs) and glycosyl hydrolases (GH) located in the endoplasmic reticulum (ER) and Golgi apparatus of the host to add and remove *N*-linked sugar residues from virus glycoproteins either cotranslationally or shortly after translation of the protein. Thus, the glycoproteins produced by these pathogenic viruses are host-specific, and the structures of their glycans echo those of the host because they are synthesized by the same biosynthetic machinery as the host glycoproteins.^8−10^

However, certain viruses, the so-called large or giant viruses, differ from this general view because many of them are reported to either encode, or are predicted to encode, enzymes involved in making extracellular polysaccharides, sugar nucleotides, and glycans attached to their major capsid proteins, i.e., glycoproteins. In addition, many of these viruses also encode enzymes involved in polysaccharide degradation, such as GHs and polysaccharide lyases (PL).

The evidence that some viruses have carbohydrate biosynthetic enzymes began with the discovery that the green microalga *Chlorella variabilis* NC64A acquired the ability to synthesize hyaluronic acid when infected with Paramecium bursaria chlorella virus type 1 (PBCV-1),^11^ a large virus discussed later in this review. Since this discovery in the late 1990s, reports of large and giant viruses that encode putative carbohydrate biosynthetic enzymes and produce their own glycan structures have steadily increased, with most of the reports occurring in the last 10 years. As a result, the concept of viral autonomous glycosylation has only recently entered into the literature.

The purpose of this review is to take readers on a journey into the world of large and giant viruses as seen through the lenses of carbohydrate structure, synthetic chemistry, and biochemistry. In doing so, this review will first describe the characteristics of these large and giant viruses that infect eukaryotic organisms, with a focus on those that have either known or putative genes encoding carbohydrate active enzymes. These include GTs, GHs, sugar methyltransferases, and those involved in the production of activated sugar nucleotide precursors.^12−14^ Then, the structure of the glycans, reported so far for only a few of these viruses, will be discussed along with how these findings have inspired synthetic organic chemistry. Finally, we will discuss how these findings open new avenues of research in many areas of chemistry and biochemistry.

## 2 Nucleocytoplasmic Large DNA Viruses

To introduce the reader to viruses that encode carbohydrate manipulating enzymes, we need to mention some changes that are occurring in the classification of viruses. There is an effort by the International Committee on the Taxonomy of Viruses (ICTV)^15^ to classify viruses into taxonomic schemes like those used for living organisms. The schemes include Kingdoms, Phyla, Classes, Orders, Families, Genera, and Species. Currently, there are 17 Phyla, and all the large dsDNA viruses considered in this review either belong to the Phylum *Nucleocytoviricota* and Class *Megaviricetes* or are predicted to eventually be classified in this phylum and class, such as pithoviruses, pandoraviruses, and molliviruses.^16,17^ For the families (or the subfamilies or the genera) whose names are proposed but not yet approved by the ICTV committee, the term “proposed” will precede their name when discussed in this review.

**Figure 1:**
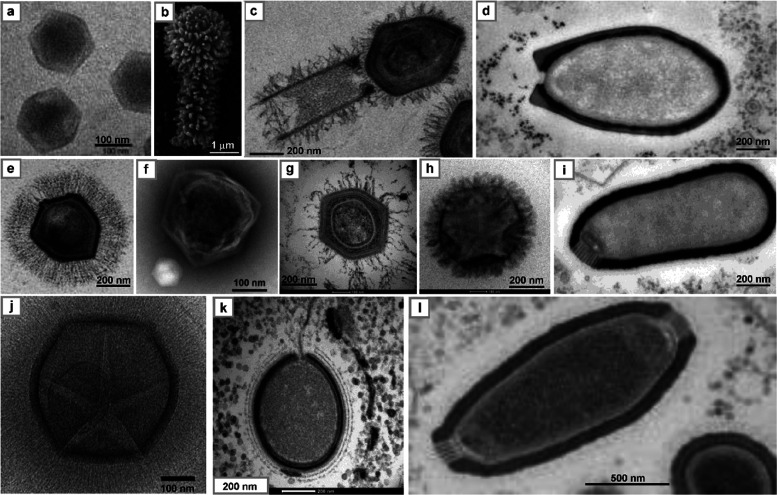
Electron microscopy images of selected giant viruses. SEM or TEM images of different giant viruses taken to display the morphology or other morphological features; their classification is reported in Table 1 along with other features. (a) PBCV-1, a member of the *Phycodnaviridae* family (genus *Chlorovirus*) and is an icosahedral virion (see also section 3.2.1). (b) Tupanvirus soda lake (clade D of *Mimivirus* genus): tridimensional image detailing the morphology of the virus, unique in having a long tubular tail (extending along the lower part of the image) attached to the capsid (top part), and fully covered with fibrils. Adapted with permission from ref (76). Copyright 2018 Springer Nature. (c) Different view of Tupanvirus soda lake virion: here the tail is defined from the two boundaries that extend down to the lower left corner of the image, while the capsid is at the up-right corner; both capsid and tail are covered with a dense layer of fibrils. Adapted with permission from ref (76). Copyright 2018 Springer Nature. (d) Pandoravirus dulcis (proposed *Pandoraviridae* family), an amphora shaped virus with an ostiole-like depression located at the left-end of the particle. (e) APMV (clade A of *Mimivirus* genus): the particle has an icosahedral shape covered with a dense layer of long fibrils. Adapted with permissoin from ref (77). Copyright 2008 Public Library of Science. (f) CroV and its virophage Mavirus (lower left corner). Reproduced with permission from ref (83). Copyright 2019 Public Library of Science. (g) Moumouvirus australiensis (clade B in *Mimivirus* genus): its icosahedral virion differs from the other in the same clade with a sparse number of fibrils attached to the capsid. (h) Moumouvirus maliensis (clade B in *Mimivirus* genus): image detailing the nature of the fibrils, shorter than those of APMV, and denser than those of Moumouvirus australiensis (g). (i) *Pithovirus sibericum*, an amphora shaped virus with a cork-like element at the left side of its longitudinal edge. (j) APMV image (cryo-EM) showing an area of lesser density (lighter gray shading) between five fibered faces, the stargate on mature particle. Adapted with permission from ref (84). Copyright 2009 Public Library of Science. (k) Mollivirus sibericum, a virus characterized by a round-shaped morphology. (l) Cedratvirus mature particle with the two cork-like elements visible at the opposite longitudinal edges. Adapted with permission from ref (85). Copyright 2009 Multidisciplinary Digital Publishing Institute. All of the images are from the authors of this review except where specified.

Collectively, large and giant viruses are often referred to as nucleocytoplasmic large DNA viruses (NCLDVs), and this term will be used in this review when referring to the group. The NCLDV group is rapidly expanding, and currently it includes several families of viruses, which differ in morphology (Figure 1), replication cycles in the host, and number of encoded genes. NCLDVs are found in diverse habitats and infect various organisms, mostly protists and microalgae. However, some NCLDVs, such as poxviruses and asfarviruses, impact animal health and food security^18^ but will not be considered in this review because they lack genes encoding GTs. The dsDNA genomes of some NCLDVs are large, going from about 300 kbp to over 2.5 Mbp (Table 1), and their genetic complexity can be larger than many small cellular organisms, like the bacterium *Mycoplasma genitalium* and the archaean *Nanoarcheum equitans* or the parasitic eukaryotic *Encephalitozoon* species.

Interestingly, NCLDV genomes encode a repertoire of putative uncharacterized proteins because their amino acid sequences lack any resemblance to annotated proteins, and for this reason are termed ORFans. Regarding the restricted pool of proteins with some homologies with known proteins, many have cell-metabolism like properties, such as components of the translational system, proteases, and other elements of the protein degradation machinery. Moreover, a certain set of these viruses encode putative proteins able to manipulate sugars at different levels: from the biosynthesis of nucleotide activated precursors, to those used to build glycans, namely GTs, and the hydrolases involved in the degradation of these molecules.

The next sections will briefly describe the properties of representative members of each of the NCLDV families discussed in this review (Table 1). Selection will be driven by the presence of putative GT encoding genes in their genome, as these genes more than others indicate the potential of these viruses to synthesize either glycans independently, or with a limited usage, of the host machinery.

### 2.1 Family Marseilleviridae

Since the discovery of the first member, Marseillevirus marseillevirus, in 2009,^19^ the family has rapidly expanded with new isolates coming from all kinds of environments. They all infect **Acanthamoeba** and belong to at least five phylogenetically related clades, each labeled with a letter (Table 1).^20^ This family has some affinity with *Iridoviridae*, even though a majority of their genes are ORFans. The icosahedral virions have a diameter of ∼250 nm and enclose AT-rich 350–390 kb dsDNA circular genomes encoding 45– 550 proteins.^21−23^ The prototype Marseillevirus marseillevirus, codes for six GTs with only two characterized by biochemical studies: L230, hydroxylates the lysine residues of a collagen-like viral protein, and transfers a Glc unit to it,^24^ and R707,^25^ discussed latter in this review (section 3.3.3).

The *Marseilleviridae* family members appear to be prone to horizontal gene transfer and genome rearrangements, localized to one side of the genome.^26^ Surprisingly, they encode histone doublets^27^ that form unstable nucleosomes to compact and package the viral genome in capsids.^28,29^ They enter the host by phagocytosis and lose their icosahedral appearance to become spherical after disappearance of the vacuole membrane. At this stage, the viral nucleosomes probably unfold to allow early transcription. Marseilleviruses encode an RNA polymerase that is not packaged in the virion and therefore must rely on the host for early transcription. During early transcription, the cell host nucleus starts changing appearance and becomes leaky through an unknown mechanism triggered by the viral infection.^20^ The nuclear proteins are recruited to the early viral factory (VF, a transitory organelle developed by the virus in which replication and viral assembly takes place), located in the cytoplasm, including the host RNA polymerase that carries out early transcription. After a few hours post infection (PI), the host nucleus morphology is restored and the viral-encoded RNA polymerase performs intermediate and late transcription in the VF.^30^ A predicted conserved promoter motif is not sufficient to explain the temporal classes of transcription, suggesting transcription factors drive the process.^30,31^ The VF expands and icosahedral particles assemble inside this host cytoplasmic region. Mature particles gather in large vesicles^32^ and cell lysis leads to the release of both individual virions and virus-filled vacuoles. The cryo-EM structure of the capsid was obtained for two members of the family at various resolutions revealing an icosahedral shape of the virion and a complex organization of the capsids^33−35^ with a major and many minor capsid proteins like the chlorovirus PBCV-1 (Figure 1a, section 2.6.1)^36^ and Cafeteria roenbergensis virus (CroV, a member of the *Megavirinae* subfamily, section 2.3.2, Figure 1f).^37^

**Table 1:** Principal Features of the Large and Giant Virus Isolates That Encode (Putative) Glycosyltransferases^a^

					virion			
family	subfamily	genus	clade	species or prototype	type	size (nm)	dsDNA (Mb)	natural host or lab replication host
*Marseilleviridae*		*Marseillevirus*	A	Marseillevirus marseillevirus	icosahedral	250	0.35–0.38	*Acanthamoeba*
			B	Lausannevirus			0.39	
			C	lnsectomime virus			0.36	
			D	Brazilian virus			0.36	
			E	Golden marseillevirus			0.38	

*Medusaviridae				Medusavirus	icosahedral	260	0.38	*Acanthamoeba*

*Mimiviridae*	*Klosneu- virinae^b^			*Bodo saltans* virus	icosahedral with fibrils	300	1.39	*Bodo saltans*
				Yasminevirus		330	2.1	*Vermamoeba*
				Fadolivirus		300	1.5	

	*Megavirinae	*Cafeteria-virus*		CroV	icosahedral	300	0.69	*Cafeteria roenbergensis*

		*Mimivirus*	A	APMV	icosahedral with fibrils	500 + 250	1.18	*Acanthamoeba*
			B	Moumouvirus		420 + 200	1.02	
			C	Megavirus chilensis		520 + 150	1.26	
			D	Tupanvirus deep ocean	icosahedral with fibrils and tail	450 + 150 + 550	1.44–1.51	*Acanthamoeba*, *Vermamoeba*
			E	Cotonvirus japonicus	Icosahedral with fibrils	400 + 200	1.48	*Acanthamoeba*

*Molliviridae				Mollivirus sibericum	spherical	650	0.65	*Acanthamoeba*

*Pandoraviridae			A	Pandoravirus salinus	amphora	1000 × 500	2.5	*Acanthamoeba*
			B	Pandoravirus neocaledonia			2.0	

*Phycodnaviridae*		*Chlorovirus*		PBCV-1	icosahedral	190	0.33	inland water green microalgae
		*Coccolithovirus*		Emiliania huxleyi virus		100–220	0.41	*Emiliania huxleyi*
		*Phaeovirus*		Ectocarpus siliculosus virus 1		120–150	0.15–0.35	marine brown algae
		*Prasinovirus*		Micromonas pusilla virus SP1		100–130	0.18–0.22	marine green microalgae
		*Prymnesiovirus*		CpV PW1		150–250	0.37–1.42	marine microalgae
		*Raphidovirus*		Heterosigma akashiwo virus		100–220	0.30	marine microalgae

*Pithocedratviridae		*Cedratvirus		Cedratvirus A11	amphora	1500 × 500	0.46–0.59	*Acanthamoeba*
		*Pithovirus		Pithovirus sibericum			0.61	

a The classification follows the criteria used by the ICTV; for the viruses whose position is proposed but not yet defined, the name of the family or of the subfamily is starred. Families and/or subfamilies can be further divided into clades (denoted with a letter) or into genera (denoted with a name). For each family/subfamily, the prototype is indicated based on the ICTV classification in 2020. After this date, type species were abolished by the committee.

b For this proposed subfamily, there is no prototype representative, accordingly the three isolates have been reported.

### 2.2 Proposed *Medusaviridae* Family

Currently, there are just two isolates in this family,^38^ both named *Medusavirus*, which infect *Acanthamoeba*.^39,40^ A low-resolution structure of the virion by cryo-EM reported an icosahedral shape with the capsid covered by spherical-headed 14 nm spikes, each extending from the capsomer, the building block of the capsid is in turn made of three copies of the major capsid protein (MCP).^40^ The icosahedral virions have a diameter of 260 nm and enclose a 381 kb dsDNA GC-rich genome, encoding more than 400 putative proteins, two-thirds of which are ORFans. Virus DNA replication takes place in the host nucleus while virions assemble in the VF in the cytoplasm; information regarding the entry and egress of the virions has not been reported. Genes used to manipulate the DNA replication machinery, such as DNA topoisomerase II and RNA polymerase, are lacking. However, a large repertoire of histones are encoded by the viruses, with the majority of them detected by in silico analyses and they are involved in the packaging of the DNA into the capsids, similarly to that reported for *Marseilleviridae*.^29^ Regarding the ability of medusaviruses to manipulate sugars, the two isolates encode one putative GT each, and the isolate with the largest genome also has a putative NDP-sugar dehydrogenase gene.

### 2.3 Family *Mimiviridae*

The first representative of the *Mimiviridae* family, Acanthamoeba polyphaga Mimivirus (APMV), was isolated from a cooling tower in Bradford (UK) in 1992. It was originally mistaken as an intracellular bacterium infecting an *Acanthamoeba* spp. because of its large size and positive Gram staining. However, infected cells imaged by electron microscopy later revealed a typical viral icosahedral morphology (Figure 1e,j), and its viral origin was confirmed by genome sequencing.^41,42^ The genome is a 1.2 Mb dsDNA, with a GC content of 28%, encoding more than 1000 proteins, including several components of the protein translation machinery. The APMV virion has a 500 nm diameter, and it is covered by a dense layer of glycosylated fibers, 120–140 nm long (Figure 1e,j).

All members of this family have dsDNA AT-rich linear genomes, and they have one internal lipidic membrane underneath the capsid shell and one delineating the nucleoprotein core. They all replicate in large VFs in the host cell cytoplasm, where the infectious cycle starts.^43−47^ All of these viruses encode a RNA polymerase and transcription maturation machinery that includes a mRNA capping enzyme, a RNase, a poly A polymerase, and methyltransferases that are packaged in the virions. As a result of this complete transcription and replication machinery, the VF can be the target of viral infection by so-called virophages, which are true viruses of viruses (Figure 1f).^48−54^ As of today, members of the *Mimiviridae* family, along with those in the genus *Prymnesiovirus* (section 2.6.5) are the only known viruses allowing the replication of virophages. Finally, these viruses encode complete DNA repair machinery,^55,56^ an asparagine synthase,^57^ as well as numerous glyco-related genes.^24,25,58−65^

It has been proposed to divide the family into two subfamilies, the *Klosneuvirinae* and the phylogenetically related amoeba-infecting *Megavirinae* (Table 1).^66^

#### 2.3.1 Proposed Subfamily *Klosneuvirinae*

All members of this proposed subfamily were initially identified from metagenomic data from bottom sediments of reservoirs at a wastewater treatment plant in Klosterneuburg (Austria).^67^ Subsequently, three different viruses have been isolated, enabling the characterization of their morphology, along with their replication style.

Bodo saltans virus (BsV) was the first isolated member of this subfamily.^68^ It infects the marine kinetoplastid *Bodo saltans*, a group of flagellated protists. The icosahedral capsids are 300 nm in diameter and contain an AT-rich linear genome of ∼1.4 Mb, encoding ∼1225 predicted proteins. Forty percent of the genes correspond to ORFans and, like other members of this subfamily, BsV encodes a complete DNA replication and repair system, along with several genes able to manipulate sugars at different levels: nine GTs, one GDP-Man 4,6-dehydratase, a Man 6-phosphoisomerase, and a 4,6 dehydratase 5-epimerase. The icosahedral capsid is made of two proteinaceous layers surrounded by 40 nm long fibrils. An apparent stargate-like structure, similar to that of APMV (Figure 1j) and other mimiviruses (section 2.3.2.2), is present at one vertex of the capsid. This structural motif is relevant during the initial stage of viral infection because it mediates the capsid opening and the creation of a channel for the delivery of the viral genome into the host cytoplasm.^69^ Finally, there are two membranes, one lining the external protein shell and one internal to the nucleoid compartment containing the genome. In contrast to other members of the proposed *Megavirinae* subfamily, BsV lacks tRNA genes, and there are only two genes encoding aminoacyl tRNA synthetases (aaRS). The infectious cycle is comparable to other members of the *Megavirinae* except that the host nuclear genome is degraded by the end of the infection cycle. Lipid vesicles are recruited for virion assembly, which takes place at one side of the VF, and mature virions are released by budding in vesicles from the host membrane and ultimately after cell lysis.^68^

Regarding the two other members of the *Klosneuvirinae*, both have recently been isolated by using *Vermamoeba vermiformis* as the host in laboratory conditions, thus, their natural host is yet to be defined.^70,71^ In temporal order, Yasminevirus was the second member to be discovered. It has an icosahedral shape of about 330 nm and the capsid is covered with a thin layer of fibrils. Its dsDNA is ∼2.1 Mb, with a GC content of 40.2%, and it codes for 1541 putative proteins including a nearly complete translational system, consisting of 70 tRNAs that are predicted to recognize 20 amino acids, along with 20 aaRSs and several translation initiation, termination, and elongation factors.^70^ The repertoire of carbohydrate active enzymes includes 10 putative GTs, along with one glycan debranching enzyme, a glycosyl hydrolase, an UDP-Glc 4-epimerase, and enzymes active on (1→4)-α-glucans, along with a glucosamine-fructose 6-phosphate amidotransferase (GFAT).

The last member of this proposed subfamily was isolated in 2021 from an Algerian sewage sample. Fadolivirus has an icosahedral shape, with a diameter of about 300 nm covered with short fibrils. Its genome is ∼1.5 Mb and encodes 1452 predicted proteins, including 66 tRNAs, 23 aaRSs, and a wide range of transcription factors, that collectively surpass the numbers found in *Klosneuvirinae* and other giant viruses.^71^ Regarding the number of predicted glycogenes, analysis of the annotated genome returns 30 GTs, three genes involved in Kdo (3-deoxy-d-*manno*-oct-2-ulosonic acid, see section 3.1.2.4) synthesis, and six other enzymes able to manipulate sugar nucleotides at different levels: dTDP-d-Glc 4,6-dehydratase, UDP-Glc 6-dehydrogenase, NDP-sugar epimerase, UDP-GlcNAc 2-epimerase WecB-like protein, NDP-sugar epimerase, NAD-dependent epimerase/dehydratase, along with a chitin synthase and a chitinase.

#### 2.3.2 Proposed Subfamily *Megavirinae*

##### 2.3.2.1 Genus *Cafeteriavirus*

Related viruses belonging to the *Megavirinae* subfamily infecting unicellular eukaryotes other than amoeba have been described. Of note, the Cafeteria roenbergensis virus (CroV, Table 1, Figure 1f), the representative of the *Cafeteriavirus* genus, is a marine virus infecting the widespread heterotrophic flagellate *Cafeteria roenbergensis*.^59^ Its icosahedral 300 nm in diameter capsid is devoid of fibrils, it has an internal lipid-containing membrane,^37^ and it contains a 618 kb dsDNA AT-rich genome, with about a third of the genes in common with its closest relative APMV, including an isoleucyl-tRNA synthetase.

Analysis of the CroV genome identified a 38 kb cluster encoding several glycogenes. It is predicted to encode the complete pathway for Kdo biosynthesis (section 3.1.2.4), along with five putative GTs, one UDP-Glc 6-dehydrogenase, and additional sugar modifying enzymes, such as sugar methyltransferases.^72^

Currently, the CroV mode of infection is not completely understood, but a nucleoid structure was reported in the cytoplasm as well as extracellular empty capsids, supporting an external opening of the capsids followed by fusion of the internal membrane with the cell plasma membrane, allowing the transfer of the nucleoid to the cytoplasm. Virions contain about 150 proteins, including several DNA repair enzymes and an ion channel protein that could protect the genome from damages.^72^ The transcription machinery packaged in the virion initiates the early formation of the VF using the same stringent early promoter as APMV.^59^ Neo-synthesized virions assemble during the late stage of infection and are released by cell lysis.

CroV was isolated in association with the virophage Mavirus (Figure 1f).^49^ Mavirus uses the late VF for gene expression and replication, interfering with the CroV infectious cycle. Mavirus is also able to enter the host cell and integrate its genome into the host genome, like lysogenic or temperate viruses. Mavirus remains silent until the cell becomes infected by CroV, which leads to VF formation and Mavirus replication. As a result, Mavirus virion production has a protecting effect on the algal cell population against CroV replication.^73^

##### 2.3.2.2 Genus *Mimivirus*

Since the discovery of mimivirus APMV in 2003 (Figure 1e,j),^41,42^ several dozen members of the *Mimivirus* genus have been isolated worldwide that belong to five clades (Table 1).^46,74−76^ They have large complex AT-rich genomes up to 1.5 Mb (Table 1), and they all have genes involved in translation initiation, elongation, and termination and can encode up to 20 aaRS.^42,59,64,67,68^ They have icosahedral capsids of about 500 nm, decorated by a layer of glycosylated fibrils of various lengths. In *tupanviruses* (Figure 1b,c, clade D), there is a long tail linked to the capsid also with long fibrils.^42,65,68,76^

The virions carry an RNA polymerase and transcript maturation machinery into the host, and they have exclusively cytoplasmic replication cycles.^45,59,68,77^ They infect their amoeba host by triggering phagocytosis after adhesion to the cell membrane by the glycosylated fibrils. Once in the vacuole, a specific structure, the stargate (Figure 1j), located at one vertex of the icosahedron capsid opens, pulling the internal membrane outside the capsid to fuse with the vacuole membrane.^69^ A compartment, coined nucleoid, is transferred into the host cytoplasm,^46,72,78^ where early transcription begins using the virally encoded transcription machinery, which remains first confined in the nucleoid.^44^ The VF in which nucleic acids accumulate due to active transcription and replication increases in size and neosynthesized virions start budding at its periphery, recycling host cell membranes derived from the ER,^44,79,80^ or Golgi compartments, as recently described for Cotonvirus (clade E).^81^ The last step in virion maturation, after genome loading into the nucleoid, is the addition to the capsid of the fibril layer, which comprises many proteins and two different polysaccharides (section 3.2.2).^80,82^ Hundreds of neosynthesized virions are released after cell lysis.

*Megavirinae* members can be infected by two kinds of virophages, Sputnik^48,51^ and Zamilon.^51^ They can also be found associated with transpovirons, which are 7 kb linear episomes.^51,86^

With regard to glyco-related genes, all of the members of this genus encode for several GTs and other enzymes involved in sugar metabolism, as discussed in section 3.1.2.5.^87^

### 2.4 Proposed *Molliviridae* Family

The first member of the proposed *Molliviridae* family, Mollivirus sibericum (Figure 1k), was isolated from the same 30 000 year old Siberian permafrost sample as Pithovirus sibericum (Figure 1i).^88^ Currently, there are only two members in this family, Mollivirus sibericum and Mollivirus kamchatka, both with some affinity with the *Pandoraviridae* family (Figure 1d), including the ability to create genes *de novo*.^89^ The capsid, haloed by fibrils, appears almost spherical with a closed ostiole-like depression, a thick tegument made of at least two layers and an internal lipidic membrane. The capsid encloses linear dsDNA, GC-rich genomes up to 650 kb, encoding ∼520 putative proteins. Two-thirds of the genes are ORFans and 16% have their closest homologues in *Pandoraviridae* members.^88^ The infectious cycle is similar to that of *Pandoraviridae*; it starts with the viral entry by phagocytosis, membrane fusion, and delivery of the compacted genome into the host cytoplasm. The genome then travels to the nucleus and uncompacts, giving access to the host RNA polymerase for early transcription until the viral encoded enzyme is produced.^88^ VF formation takes place in the cytoplasm, and there virion assembly is initiated by a membrane precursor resembling a cisterna, which later is coated with the thick tegument.^90^ DNA-associated filaments fill the nascent viral particles in the late VF, and a late stage of maturation involves a change in morphology of the virions to produce the ostiole-like depression at the initiation pole. Neosynthesized virions can be released by either exocytosis or by cell lysis.^88,90^ Finally, M. sibericum is the only member of this proposed family to contain glyco-related genes, in particular its genome harbors two putative GTs, with one predicted as specific for glucosamine.

### 2.5 Proposed Family *Pandoraviridae*

Members of this family have been isolated from all over the world in all kinds of environments (fresh water, ocean,^6^ soil,^91^ waste,^92^ patients infected with *Acanthamoeba* sp.^93^). Currently, this family includes 15 species (source NCBI/viruses)^38^ divided into two clades (Table 1).^6,91−98^ They are the most complex viruses known, endowed with linear GC-rich dsDNA genomes up to 2.5 Mb. Pandoravirus genome size can in part be explained by their ability to create new genes de novo from intergenic regions.^91,98^ Pandoraviruses genomes encode up to 1500 proteins, and again, like in other NCLDV families, the vast majority of the genes encode ORFans.^98^ However, they do encode a few putative carbohydrate enzymes: P. salinus, the prototype of clade A encodes a glucosamine transferase and the incomplete domain of a 6-phospho-glucosidase, while P. neocaledonia, a representative of the B clade, has only a partial domain of a glucosamine transferase. Interestingly, the glucosamine transferase is encoded in all of the other sequenced pandoraviruses, together with one or two other glyco-related genes, as glycosidases or hydrolases.

The virions are amphora shaped, about 1 μm long and 500 nm wide, with an ostiole-like depression closed by a plug-like structure at one apex (Figure 1d). There is at least one lipidic membrane lining a thick tegument made of three layers, including one made of cellulose.^99^

The infectious cycle starts, like all amoeba infecting viruses, by phagocytosis of the particles followed by opening of the plugged depression to allow fusion of the internal membrane with the phagosome membrane, which allows transfer of the genome and necessary proteins into the host cytoplasm. However, unlike many NCLDV members, pandoraviruses build their VF in the host cell nucleus. They encode an RNA polymerase that is not packaged in the capsids, and so they rely on the host cell for their early transcription until the virally encoded enzyme is translated. New virions start to assemble from the apex, and the neosynthesized virions are released either by exocytosis through membrane fusion with the plasma membrane when they are in vacuoles or by cell lysis.^6,92^

### 2.6 Family Phycodnaviridae

The family *Phycodnaviridae* is in the order of *Algavirales*, which includes many other viruses that infect eukaryotic algae. The phycodnaviruses consist of a large genetically and morphologically diverse group of viruses with eukaryotic algal hosts from both inland and marine waters. The six genera in this family include *Chlorovirus*, *Coccolithovirus*, *Prasinovirus*, *Prymnesiovirus*, *Phaeovirus*, and *Raphidovirus*, all with genomes that range in size from ∼160 kb to ∼450 kb, with some exceptions for viruses tentatively assigned to the genus *Prymnesiovirus*. All six of these genera are reported by gene annotation to have enzymes involved in various aspects of carbohydrate metabolism,^100,101^ although experimental proofs that these genes encode functional enzymes have only been provided for a few members of the *Chloroviruses*.

Moreover, the *Phycodnaviridae* family includes many viruses that are currently not classified into any of the genera mentioned above. Indeed, the taxonomical position of Chrysochromulina ericina and parva viruses (CeV and CpV, respectively),^54,66,102^ Aureococcus anaphagefferens virus (AaV),^102^ and Tetraselmis virus (TetV),^103^ is currently being debated, and the alignment of their genome sequences along with those of recognized members of the *Prymnesiovirus* genus, suggests that all these viruses should be placed in a new subfamily, called *Mesomimivirinae*, belonging to the *Mimiviridae* family.^66^ In this review, all these viruses will be discussed together with the other *Prymnesioviruses*.

#### 2.6.1 Genus *Chlorovirus*

As elaborated later on in this review, the chloroviruses provide the most information about virus-encoded genes involved in carbohydrate metabolism. Chloroviruses are plaque-forming, large icosahedral (190 nm in diameter), dsDNA-containing viruses (genomes of 290–370 kb) with an internal lipid-containing membrane (Figure 1a).^104^ They exist in inland waters throughout the world, with titers occasionally reaching thousands of plaque-forming units (PFU) per mL of indigenous water. Known chlorovirus hosts, which are normally endosymbionts and are often referred to as zoochlorellae, are associated either with the protozoan *Paramecium bursaria*, the coelenterate *Hydra viridis*, or the heliozoan *Acanthocystis turfacea*.^101^

Chloroviruses are divided in groups depending on their host specificity; viruses that replicate in *Chlorella variabilis* NC64A are referred to as NC64A viruses, viruses that infect (or replicate in) *Chlorella variabilis* Syngen 2–3 are referred to as Osy viruses, viruses that replicate in *Chlorella heliozoae* SAG 3.83 are referred to as SAG viruses, and viruses that replicate in *Micractinium conductrix* Pbi are referred to as Pbi viruses. The zoochlorellae are resistant to virus infection when they are in their symbiotic relationships because the viruses have no way of reaching their host.

The genomes of 43 chloroviruses infecting these four hosts have been sequenced, assembled, and annotated.^105^ Collectively, the viruses encode 1345 predicted protein families; however, any given chlorovirus only has 330–410 protein-encoding genes (PEGs). Thus, the genetic diversity among these viruses is large, and many of the proteins are unexpected for a virus. Except for homologues in other chlorovirus members, about 66% of their PEGs do not match anything in the databases.

The prototype of this genus, Paramecium bursaria chlorella virus type 1 (PBCV-1, Figure 1a), is an NC64A virus. PBCV-1 has an icosahedral capsid with a spike-like structure at one vertex and a few external fibers that extend from some of the capsomers.^106,107^ The outer capsid layer covers a single lipid bilayered membrane, which is essential for infection. The PBCV-1 MCP (named Vp54, coded by gene *a430l*) is a glycoprotein, and three Vp54s form the trimeric capsomere, which has pseudo-6-fold symmetry (section 3.2.1).^108^ A proteomic analysis of highly purified PBCV-1 virions revealed that the virus contains 148 virus-encoded proteins and at least one host-encoded protein.^109^

The PBCV-1 genome is a linear ∼331 kb, nonpermuted dsDNA molecule with covalently closed hairpin termini. Identical ∼2.2 kb inverted repeats flank each 35-nucleotide-long, incompletely base-paired, covalently closed hairpin loop.^110^ The remainder of the PBCV-1 genome contains primarily single-copy DNA that encodes ∼410 putative proteins and 11 tRNAs.^109^ The G+C content of the PBCV-1 genome is 40%; in contrast, its host nuclear genome is 67% G+C. PBCV-1, and the other chlorovirus genomes contain methylated bases, which occur in specific DNA sequences. The methylated bases are part of chlorovirus-encoded DNA restriction and modification systems.^111^

As elaborated on below, the chloroviruses were the first viruses reported to code for sugar-nucleotide synthesizing enzymes (section 3.1.1), extracellular polysaccharide enzymes that make hyaluronan and chitin (section 3.3.1), and the GTs involved in the synthesis of the glycans attached to the major capsid glycoproteins of the virions (section 3.3.2).^13^

#### 2.6.2 Genus *Coccolithovirus*

This viral genus includes only one species, *Emiliania huxleyi* virus (EhV), an enveloped virus of about 250 nm in size,^112^ further divided into different strains all able to infect unicellular algae.^38^ Of note, the alga *E. huxleyi* is known for its massive seasonal blooms that generally collapse within 5–8 days, and several studies have shown that bloom termination occurs upon viral infection, thus inferring a relevant role to this type of virus in maintaining the balance of the ecosystem.

Regarding the features of EhV, the strain EhV-86 is the one used as the reference,^113^ and it has a circular dsDNA genome of 407 kb length, with a 40.2% G+C nucleotide composition, encoding 472 putative proteins, with about 80% annotated as hypothetical proteins. As for the others with an assigned function, there were five tRNAs, six RNA polymerases, eight proteases, as well as at least four genes that encode proteins involved in sphingolipid biosynthesis, along with four glyco-related genes: two GTs and two GHs.

#### 2.6.3 Genus *Phaeovirus*

Viruses belonging to this genus have an icosahedral capsid of 120–150 nm, with linear dsDNA genomes of 150–350 kb in length. Phaeoviruses differ from the other genera in this family. First, phaeoviruses do not replicate in a unicellular algal host, but in filamentous brown algae, including members of the *Ectocarpus* and *Feldmannia* genera, and second, they are the only giant viruses whose replication occurs via a lysogenic infection cycle instead of the classical lytic mechanism. The phaeoviruses life cycle starts by infecting the free-swimming, wall-less gametes or spores of their hosts before they develop into mature algae. Hence, the viral DNA is first integrated into the spore genome, where it is replicated together with the host DNA and transmitted to the mature algae, where it is later expressed in the nuclei of the reproductive organs. At this stage, the host nucleus becomes disrupted, and the viral assembly continues in the cell until it is densely packed with viral particles. The new virions are then released by cell lysis into the surrounding water, and they are ready to start a new infection cycle.

Collectively, this genus consists of nine species, each of which can infect several algal isolates. The genome of only two phaeovirus species, *Feldmannia* and *Ectocarpus*, have been completely sequenced. Of these two species, only Ectocarpus siliculosus virus 1, which codes for about 230 proteins, has genes related to carbohydrate metabolism. Three such genes are predicted to encode a alginate epimerase, a sugar nucleotide dehydrogenase, and a GT.^114^

#### 2.6.4 Genus *Prasinovirus*

Viruses assigned to this genus infect marine prasinophytes (a group of unicellular green algae) from three genera in the classes *Mamielloophyceae*: *Bathycoccus*, *Micromonas*, and *Ostreococcus*. Members of the *Ostreococcus* genus are the world’s smallest free-living eukaryotes. With a small host size (less than 1 μm in diameter for *Ostreococcus*) and a virus capsid size of around 120 nm, it is estimated that there is physically room for no more than 100 virions at any one time. This is reflected in experimental data that suggest a typical burst size, i.e., number of infectious viral particles produced from one infected host cell, of 6–15 viruses per cell.^115^

Prasinoviruses are ubiquitous in seawater throughout the world and genomic sequences from inland waters suggest that some of them may also infect freshwater algae. Their genomes range in size from 180 to 215 kb, with a G+C content range from 35 to 50%, encoding 203–269 compactly arranged proteins (with an average intergenic distance of about 40 bp), 4–11 tRNAs, and terminal inverted repeats of 250–2150 bp. Prasinovirus particles are icosahedral, with diameters of 100–130 nm. Many of the prasinoviruses are sensitive to chloroform, which suggests that they have a lipid-containing membrane; however, others do not loose infectivity after exposure to chloroform.

Following viral infection, genome replication begins about 2 h PI, virions assemble in the cytoplasm from 6 to 20 h PI, after which cellular lysis occurs. The host cell nucleus, mitochondria and chloroplast remain intact throughout this period. According to the ICTV classification,^15^ this genus includes two species, *Micromonas pusilla* virus and *Ostreococcus tauri* virus, although many others have been proposed to belong to this genus. In fact, more than 30 of these viruses have been completely sequenced.^38^ They have clusters of genes predicted to code for the synthesis of sugar nucleotides and many GTs, suggesting the presence of complex glycosylation pathways in these viruses.^73,116,117^ However, no one has investigated this aspect of their life cycle. In fact, it is not even known if their MCPs are glycosylated, although it is very likely they are.

#### 2.6.5 Genus *Prymnesiovirus*

Members in this genus, such as Chrysochromulina brevifilum virus (CpV) and Phaeocystis globosa virus (PgV),^52^ have all been isolated from marine environments where they infect unicellular eukaryotic algae. They have icosahedral capsids, which are about 150–400 nm in diameter, and dsDNA linear AT-rich genomes of at least 370 kb and up to 1.42 Mb for Prymnesium kappa virus RFO1 (PkV-RFO1).^64^ Up to 45% of the viral encoded genes are ORFans and encode UV-DNA repair machinery and two copies of the RNA polymerase subunit 2. The VF is built in the cytoplasm, but it is unknown if the transcription machinery is packaged in the capsids, leading to an entirely cytoplasmic infectious cycle. They can encode a functional rhodopsin,^52,118−122^ proteins involved in energy production, and up to two aaRSs.^64,68,122^ Virophages have been isolated in association with CpV^54^ and PgV,^52^ and several other virophages were identified in metagenomic data with several members of this genus.^123−127^ PkV-RFO1 has the largest genome and appears distantly related to other members of this subgroup with 40% ORFans. The PkV genome is enriched in putative genes encoding lipid metabolism and membrane biogenesis, carbohydrate transport, and metabolism and energy production and conservation. Interestingly, PkV RF01 encodes 48 putative GTs, the highest number so far predicted in NCLDV, in addition to seven GHs, four carbohydrate esterases, and one PL.^64^

As for the other members in this genus, based on genome annotation, CeV^54^ encodes for 13 GTs, while AaV^102^ codes for sugar degradation and sugar binding proteins, and 10 GTs, while TetV^103^ possesses a saccharide degradation enzyme α-galactosidase, a mannitol metabolism enzyme mannitol 1-phosphate dehydrogenase, and two key genes in algal fermentation pathways: pyruvate formate-lyase and pyruvate formate-lyase activating enzyme.^103^ The presence of these latter two genes suggests that TetV has the unprecedented capacity to manipulate its host’s fermentation pathway, which has intriguing implications for the ecology of the virus, such as its potential to spread in hypoxic/anoxic environments and/or the ocean biochemistry given that TetV infections may alter the production of fermentation products such as ethanol.^103^

#### 2.6.6 Genus *Raphidovirus*

Like Coccolithovirus, this genus includes only one species, *Heterosigma akashiwo* virus (HaV), named for its host, *Heterosigma akashiwo*,^128^ a marine microalgae responsible for harmful algal blooms, and for this reason named with the equivalent of the Japanese word *akashivo*, “red tides”. The HaV particle is icosahedral, with a diameter of about 200 nm, with a linear dsDNA of about 275 kb encoding about 250 proteins. HaV does not encode a DNA-directed RNA polymerase or polyA RNA polymerase, indicating that this virus depends entirely on the host’s transcription machinery that it manipulates by using its own transcription initiation factors, mRNA capping enzyme subunits, and ribonuclease III.^129^ Regarding the presence of glycogenes, HaV-1 encodes for four putative GTs, in addition to a putative GDP-Man dehydratase and an epimerase, that taken together collectively demonstrate that this virus has the ability to manipulate sugars at different levels.

### 2.7 Proposed *Pithocedratviridae* Family

Since the isolation of the first Pithovirus from a 30 000 years old permafrost sample,^5^ many additional members have been isolated and can be grouped into two distantly related genera *Pithovirus*,^130^ and *Cedratvirus*,^85,131,132^ here discussed together. These viruses have large amphora shaped capsids that can be up to 2 μm long and 600 nm wide. They are closed by one cork for Pithovirus (Figure 1i)^5,130^ and two corks for Cedratvirus (Figure 1l),^85^ made by proteins organized in a honeycomb array. Even though the virion’s overall morphology resembles that of *pandoraviruses* (Figure 1d), the external tegument is different because they do not have cellulose and the tegument appears to be made of parallel strips, coated with short sparse fibrils.^5,133^ The pithovirus and cedratvirus AT-rich genomes are circular or circularly permutated and ∼610 kb and ∼460–589 kb, respectively. Of note, members of Pithovirus and *Cedratvirus* genera have been defined as “empty vessels” because despite being the largest giant viruses in terms of virion size (Figures 1d,f), their genomes are smaller compared to those of other giant viruses such as pandoraviruses (2.5 Mb, amphora-shaped virion of 1000 nm × 500 nm) and Yasminevirus (2.1 Mb, icosahedron capsid of 330 nm, Table 1).

*Pithovirus* and *Cedratvirus* share 80% of their genes and encode up to 450 proteins, including some putative carbohydrate active enzymes.^85^ For example, the Brazilian cedratvirus IHUMI strain IHUMI-277 encodes seven putative enzymes involved in carbohydrate metabolism, including three GTs and a protein with a rhamnan synthesis domain. Similarly, Cedratvirus A11 encodes for five GTs, with one predicted as a *N*-acetylglucosaminyltransferase, a dTDP-d-Glc 4,6-dehydratase, and a NAD dependent epimerase/dehydratase. Pithovirus sibericum, the only species completely sequenced in the genus,^38^ encodes four putative GTs, along with a NAD-dependent epimerase/dehydratase.

The infectious cycle of these two proposed virus genera proceeds like other amoeba infecting viruses, namely by phagocytosis followed by capsid opening and membrane fusion with the phagosome.^47^ For the pithoviruses and cedratviruses, the viral encoded RNA polymerase is packaged in the virion, which initiates early transcription in the cytoplasm and the host nucleus remains intact throughout the replication cycle. The virions are assembled starting from one apex of the future virion, leading to a rectangular uncoated virion, then the tegument is built by patches from a reservoir in the cytoplasm and the virus morphology changes to amphora-shaped. The neosynthesized virions can exit by exocytosis and by cell lysis.^5,85^

## 3 Biosynthetic Machinery and the Glycans Produced by Giant Viruses

Before the discovery of giant viruses, the presence of glycan biosynthetic enzymes in the viral world was limited to a few eukaryotic viruses that encode for GTs as a strategy to increase their survival and propagation rate.^12^ The first report dates back to 1960, with the discovery that bacteriophages T2, T4, and T6 of *Escherichia coli* encode a GT able to transfer a Glc residue to the hydroxymethylcytosines of their DNA to protect it from digestion by host restriction endonucleases.^12^ Baculoviruses infecting *Lepidoptera* larvae are another example: these viruses express a GT that attaches Glc to the larval hormone ecdysone. Glucosylation inactivates the hormone, leading to the larva remaining in the pupation phase. This strategy allows baculoviruses to complete their infection cycle.^12^ Bovine herpesvirus and Mixoma virus are two other examples; the former encodes a β-(1→6)-GlcNAc transferase,^12^ while the latter encodes a α-(2→3)-sialyltransferase.^134^ The viruses mentioned above were considered to be exceptions to the rule that viruses infecting eukaryotes exploit the host machinery to produce glycans.^8^ However, this belief must be reconsidered as it does not apply to giant viruses.

Some giant viruses are predicted to encode most, if not all, of the machinery required for the glycosylation of their structural proteins.^14^ However, we only have hard evidence to support this claim for a handful of systems (Table 2). As noted in the first part of this review, giant viruses encode many putative glyco-related genes, including those responsible for the synthesis of sugar nucleotides, sugars modification, and sugar assembly, along with other sugar or polysaccharide manipulating enzymes. Currently, the most studied carbohydrate manipulating enzymes are from chloroviruses and mimiviruses (Table 2), where the function of some of their glycogenes and the structure of their glycans have been determined. Accordingly, this section will describe first the works that demonstrate that these viruses do indeed encode proteins used in the production of their nucleotide sugars, then it will describe the structures of their glycans, and finally the GTs involved in the process. The last part of this section will examine other viral enzymes that instead of acting on the viral glycans seem to play a role in remodeling the host glycans.

**Table 2:** Principal Features of the Glyco-Related Enzymes from Giant Viruses Whose Activity Is Based on Experimental Data^a^,^b^

genus	species	protein	protein ID	aa	domains	activity	homology	PDB	section	ref
*Chlorovirus*	ATCV-1	Z544R	YP_001427025	350	1	UGD	E		3.1.1.2	(58)
		Z804L	YP_001427285	352	1	GMD	B		3.1.1.1	(135)

	CVK2	vAL-1	BAB19127	349	1	PL	E	3GNE^c^	3.4.4	(136, 137)
		vChta-1	BAA20342	328	1	GH	B		3.4.1.2	(138)
		vChti-1	BAA78554	836	3	D1: GH	B		3.4.1.2	(139)
						D2: GH	B			
						D3: ND	ND			

	CVN1	CL2	BAA83789	333	1	PL	E		3.4.3	(140)

	PBCV-1	A061L	NP_048409	209	1	methyl-transferase	B		3.3.2.2	(141)
		A064R	NP_048412	638	3	D1: Rha-transferase	B/E	1LL2	3.3.2.2	(141−143)
						D2: Rha-transferase	B			
						D3: methyl-transferase	B			
		A094L	NP_048442	364	1	GH	B		3.4.2	(144)
		A098R	NP_048446	568	1	HAS	E	7SP6^c^	3.3.1	(11, 145)
		A100R	NP_048448	595	1	GFAT	B		3.3.1	(146)
		A111/114R	NP_048459	860	3	D1: Gal-transferase	B		3.3.2.3	(147)
						D2: Xyl-transferase	B			
						D3: Fuc-transferase	B			
		A118R	NP_048466	345	1	GMD	B		3.1.1.1	(135, 148)
		A181/182R	NP_048529	830	3	D1: CBD	B		3.4.1.1	(149)
						D2: GH	B			
						D3: NCR	B			
		A260R	NP_048613	505	1	GH	E		3.4.1.1	(149)
		A292L	NP_048646	328	1	GH	B		3.4.1.1	(149)
		A295L	NP_048649	317	1	GMER			3.1.1.1	(148)
		A561L	NP_048917	649	4	D1: NCR	B		3.4.5	(150)
						D2: NCR	E			
						D3: NCR	E			
						D4: PL^d^	E			
		A609L	NP_048965	389	1	UGDH	B		3.3.1	(146)

*Mimivirus*	APMV	L136	YP_003986628	352	1	aminotransferase	B	7MFQ	3.1.2.1	(62, 151)
		R141	YP_003986633	323	1	UGD	E	6VLO	3.1.2.1	(58, 152)
		L142	YP_003986634	490	2	D1: GT			3.1.2.1	(60)
						D2: acetyl-transferase	B			
		L230	YP_003986726	895	2	D1: Glc-transferase	E		3.3.3	(24, 153)
						D2: NCR	E	6AX6		
		L316	YP_003986819	148	1	GNAT	A		3.1.2.2	(154)
		L619	YP_003987136	606	2	GFAT	E		3.1.2.2	(154)
		R689	YP_003987216	255	1	UAP	B		3.1.2.2	(154)
		R707	YP_003987235	281	1	Glc-transferase	E		3.3.3	(25)
		L780	YP_003987312	289	1	UGER	E	7JID	3.1.2.1	(58, 155)

	Ma	Ma458	AVL94844	324	1	UGD and epimerase	ND^e^		3.1.2.5	(87)
		Ma459	AVL94845	270	1	reductase	ND^e^		3.1.2.5	(87)
		Ma460	AVL94846	376	1	epimerase	ND^e^		3.1.2.5	(87)
		Ma465	AVL94851	384	1	aminotransferase	B		3.1.2.5	(87)
		Ma466	AVL94852	209	1	acetyl-transferase	B		3.1.2.5	(87)
		Ma467	AVL94853	278	1	UGD	B		3.1.2.5	(87)

	Mg	Mg534	YP_004894585	323	1	UGD and epimerase	B	4TQG	3.1.2.3; 3.1.2.5	(61)
		Mg535	YP_004894586	270	1	epimerase and reductase	B		3.1.2.3; 3.1.2.5	(61)
		Mg536	YP_004894587	370	1	epimerase	B		3.1.2.3; 3.1.2.5	(61)

	Mm	Mm419	QGR53988	278	1	reductase	B		3.1.2.5	(87)
		Mm421	QGR53990	321	1	reductase	B		3.1.2.5	(87)
		Mm422	QGR53991	279	1	UGD	B		3.1.2.5	(87)

a Proteins are named as in the original publication, and their ID number is provided for a quick retrieval in the NCBI database. The homology column refers to the organism (all but viruses) whose protein had the best match with the viral protein: bacterium (B), archaea (A), or eukaryote (E). Moumouvirus maliensis, Mm; Moumouvirus australiensis, Ma; Megavirus chilensis, Mg.

b NCR, not related to carbohydrate/glycan manipulation; ND, not determined or reported in the publication.

c One of the crystallographic coordinates available in the publication, others PDBs are the same protein in complex with other molecules and/or at different pH.

d Putative activity.

e Assignment based on homology with proteins from other giant viruses.

### 3.1 Biosynthesis of Sugar Nucleotides

In 1997, chlorovirus PBCV-1 was the first virus reported to encode enzymes involved in the synthesis of sugar nucleotides and polysaccharides.^11^ Indeed, PBCV-1 codes for a hyaluronate synthase (section 3.3.1) and for the two enzymes involved in the synthesis of the monosaccharides that comprise the polysaccharide (section 3.1.1, Table 2). The glycan biosynthetic ability of PBCV-1 was again recognized in 2003, when the pathways the virus uses to synthesize GDP-β-l-Fuc and GDP-α-d-Rha were identified and experimentally validated (section 3.1.1.1, Table 2).^148^ This work advanced the hypothesis that PBCV-1 encoded a glycosylation machinery that was, at least in part, independent of the host. The pathways for other activated sugars that comprise the *N*-linked glycans of the PBCV-1 major capsid protein, Vp54 (l-Ara, d-Gal, d-Glc, d-Man, l-Rha, d-Xyl), are not present; we assume that the virus uses the sugar nucleotides produced from the host to build these glycans.

Years later, the biosynthetic pathways for sugar nucleotides were identified and experimentally verified for some members of the genus *Mimivirus* (section 3.1.2, Table 2).^58,60−62,154^ Interestingly, *Mimivirus* clusters the genes encoding the sugar nucleotides enzymes (section 3.1.2.5) and these clusters are clade specific, while chloroviruses do not have this gene organization.

Finally, in silico analyses suggest that CroV and Fadolivirus encode the Kdo biosynthetic pathway, described later in this review although not supported by experimental evidence due to the relevance that this monosaccharide has for plants and bacteria (section 3.1.2.4).^59^

**Figure 2:**
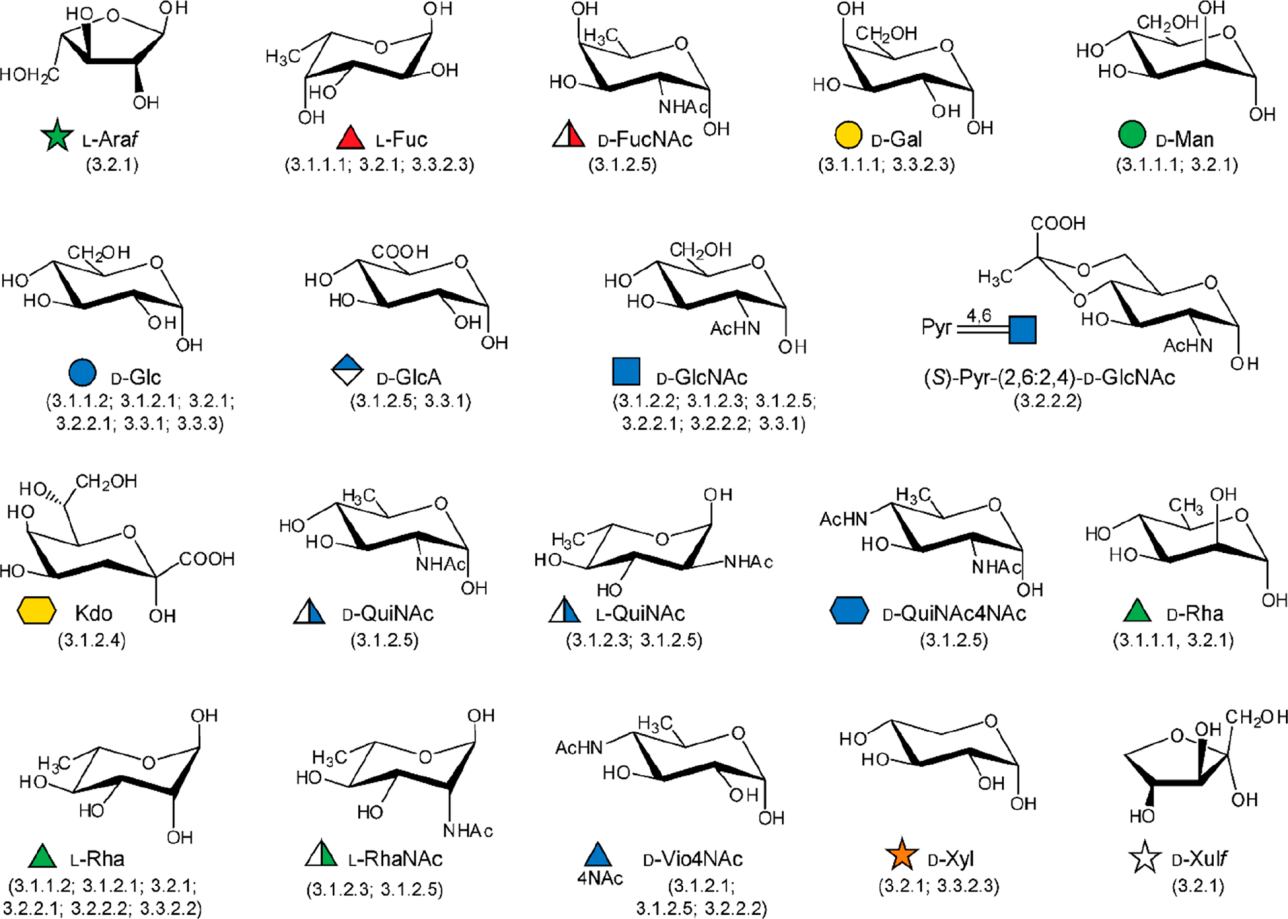
Monosaccharides diversity in giant viruses. Structure of the monosaccharides detected in the glycans of giant viruses or synthesized by them as discussed in this review (the corresponding sections are enclosed in brackets). All monosaccharide units are in the pyranose form, unless explicitly indicated (“*f*” indicates the furanose form), and α configured at the anomeric center. Each structure is accompanied by the abbreviated name of the residue and by its symbol as defined by the rules of the Symbol Nomenclature of Glycans.^156^

The type of monosaccharides found in giant viruses, either involved in the construction of the glycans or for which the biosynthetic pathway has been elucidated, are summarized in Figure 2, along with the section(s) where they are discussed.

#### 3.1.1 Chloroviruses

##### 3.1.1.1 GDP-β-l-Fuc and GDP-α-d-Rha Biosynthesis in PBCV-1

**Figure 3:**
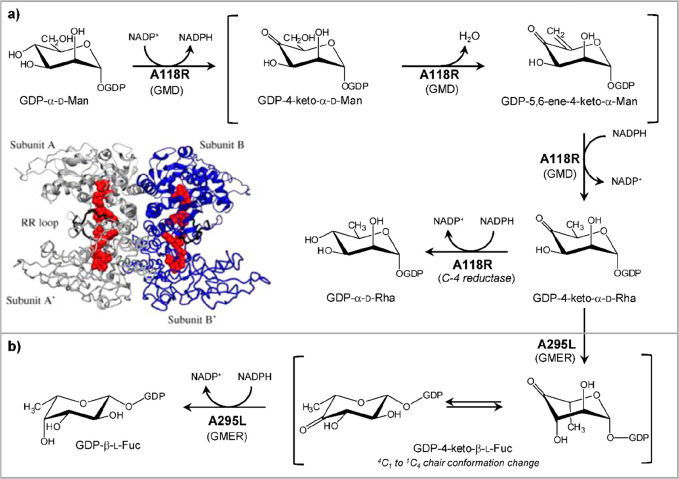
Biosynthesis of sugar nucleotides encoded by chlorovirus PBCV-1. GDP-α-d-Rha and GDP-β-l-Fuc are produced by the chlorovirus PBCV-1 by using GDP-α-d-Man from the host. The PBCV-1 genome encodes both enzymes necessary to produce these two sugar nucleotides. A118R has the GMD activity, and A295L is the GMER protein. (a) Transformation of GDP-α-d-Man into GDP-α-d-Rha catalyzed by A118R. This enzyme is unique in its class because it has two activities.^148^ In the first step, A118R transforms GDP-Man into its 6-deoxy-4-keto derivative. This transformation requires NADP^+^ cofactor that is regenerated upon formation of the 6-deoxy-4-keto sugar nucleotide intermediate. This molecule is reduced by A118R in the presence of NADPH to give GDP-α-d-Rha. The A118R crystallographic structure is reported in the left lower part of the panel. Reproduced with permission from ref (160). Copyright 2006 Elsevier. A118R has a Rossman fold, and it crystallizes as a tetramer with NADP^+^ (or NADPH) cofactors (red), promoting the association between two dimers. (b) Transformation of GDP-6-deoxy-4-keto-α-d-Man produced by A118R into GDP-β-l-Fuc by the NADPH-dependent reaction with A295L, a GDP-6-deoxy-4-keto-d-Man epimerase/reductase (GMER). The activities of the enzymes are indicated in parentheses.

Chlorovirus PBCV-1 encodes two enzymes, A118R and A295L, which are identified as a GDP-α-d-Man-4,6-dehydratase (GMD) and GDP-6-deoxy-4-keto-α-d-Man epimerase/reductase (GMER). These proteins are involved in the biosynthesis of GDP-α-d-Rha and GDP-β-l-Fuc, respectively (Figure 3).^148^ The A118R protein (345 aa) catalyzes the two-step dehydration of GDP-α-d-Man at both C-4 and C-6 carbons, producing the GDP-6-deoxy-4-keto-α-d-Man product as an intermediate (Figure 3a), which is then used by the GMER enzyme, A295L (317 aa), to make GDP-β-l-Fuc (Figure 3b). Both A118R and A295L proteins are similar to those found in bacteria and eukaryotes (Table 2),^157,158^ although the viral A118R has an additional NADPH-dependent activity.^148^ Indeed, when the recombinant A118R was incubated with GDP-α-d-Man and both NADP^+^ and NADPH, instead of NADP^+^ alone, it produced GDP-α-d-Rha (Figure 3a), indicating that PBCV-1 GMD has an additional activity that occurs only when additional NADPH is present (Figure 3a). This double function is rare, and it has only been reported in *Aneurinibacillus thermoarophilus*,^159^ whose recombinant protein produces only a small amount of GDP-α-d-Rha, suggesting that this process may not occur in vivo.

The crystal structure of A118R was solved at 3.8 Å resolution (Figure 3a), and this protein belongs to the short-chain dehydrogenase/reductase (SDR) superfamily, a group of proteins involved in sugar nucleotide biosynthesis. PBCV-1 GMD consists of two domains: an N-terminal domain binding the NADP(H) cofactor and a smaller C-terminal domain containing the sequence Gly-X-X-Gly-X-X-Gly typical of the SDR protein family, responsible for the binding of the sugar nucleotide substrate.^160^ The N-terminal domain has a modified Rossman fold structure consisting of a seven-stranded parallel β-sheet sandwiched between six α-helices, that create the binding pocket used to accommodate NADP(H) next to the residues ^125^Thr, ^149^Tyr, ^153^Lys, and the catalytic triad responsible for the activity of the enzyme. A118R can complex both NADP^+^ and NADPH, although the interaction with NADPH is stronger and is essential to stabilize the protein structure as a dimer. In contrast, A118R interaction with NADP^+^ is weaker, and this cofactor dissociates from the complex once GDP-α-d-Man is transformed into GDP-6-deoxy-4-keto-α-d-Man. The result is that the A118R dimer turns into the monomeric form that is enzymatically inactive.^160^ NADPH maintains GMD in its dimeric form, and it enables the protein to transform the GDP-6-deoxy-4-keto-α-d-Man intermediate into GDP-α-d-Rha.^135^ As for the quaternary structure (Figure 3a), A118R exists in solution as a dimer like other SDRs; however, two GMD dimers associate to give a tetrameric structure through crystallographic symmetry (Figure 3a).^160^

Hence, PBCV-1 encodes the genes necessary to synthesize the activated precursor for two of the sugars necessary to synthesize the *N*-linked glycans attached to its major capsid proteins (section 3.2.1), l-Fuc and d-Rha. In this process, the virus relies on the host for the GDP-α-d-Man substrate. Similar genes encoding putative GMD and GMER enzymes, named Z804L and Z282L, respectively, are also found in Acanthocystis turfacea chlorovirus 1 (ATCV-1).^135^ Phylogenetic analyses indicated that PBCV-1 and ATCV-1 GMERs were more similar to one another than to 20 other taxa from all forms of life and one virus.^135^

Regarding the similarities in GMD between the two chloroviruses, the sequences from ATCV-1 and PBCV-1 only had 53% identity, suggesting that the two enzymes diverged early during viral evolution and that they might have different properties. Indeed, ATCV-1 GMD biochemical characterization confirmed the expected dehydratase activity, but the protein lacked the reductase activity characteristic of PBCV-1 GMD.^135^ Moreover, by analyzing the cofactor content of the two GMD proteins when expressed in *E. coli*, ATCV-1 GMD had a high affinity for NADP^+^ rather than NADPH, as reported for the PBCV-1 enzyme. ATCV-1 GMD had both NADP^+^ and NADPH in variable ratios, while PBCV-1 protein only had NADPH.

##### 3.1.1.2 Biosynthesis of the UDP-β-l-Rha Precursor in ATCV-1

**Figure 4:**
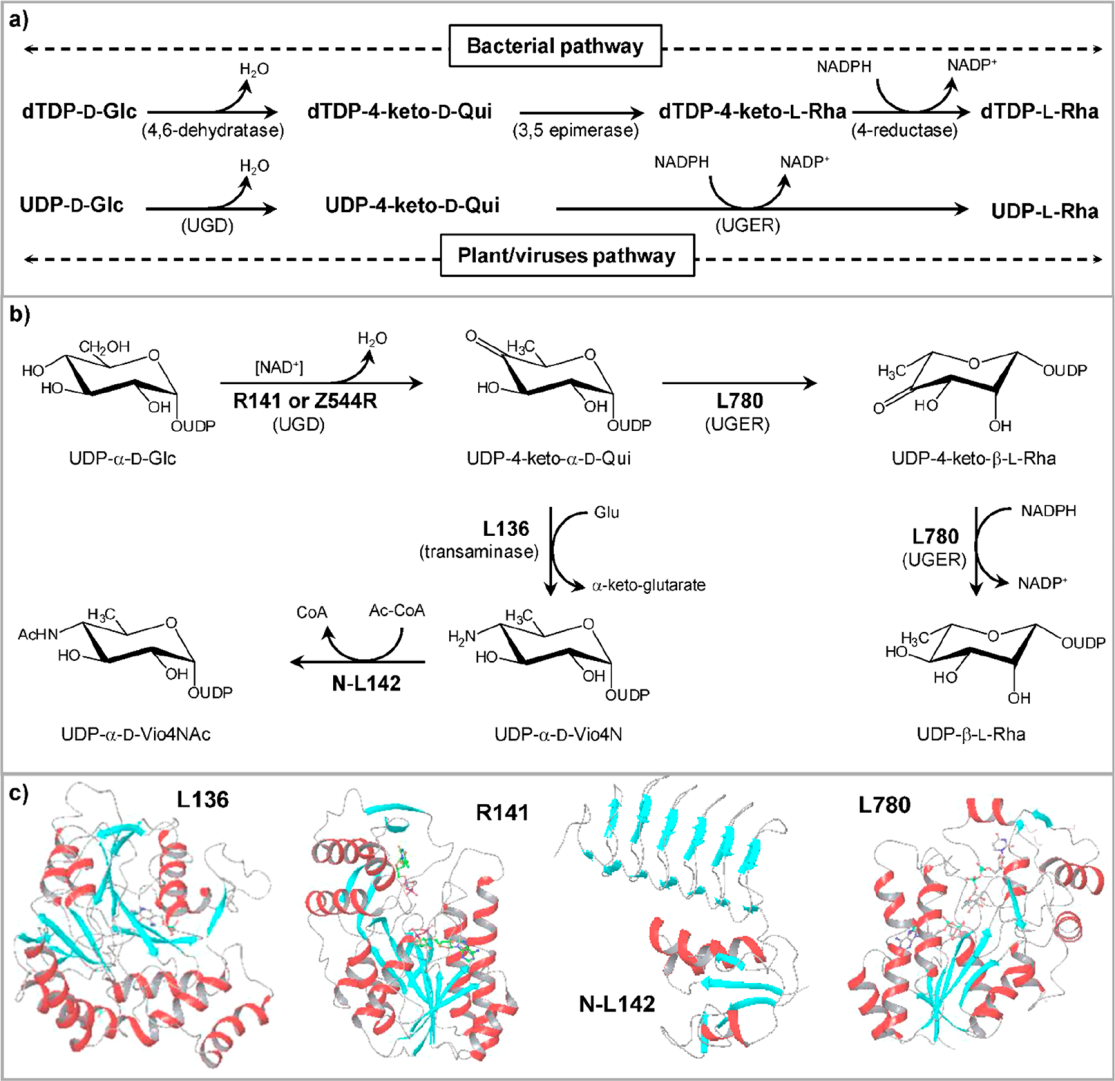
Biosynthetic pathway for UDP-β-l-Rha and UDP-α-d-Vio4NAc. (a) Comparison of NDP-β-l-Rha biosynthesis in bacteria and plants/viruses. The two pathways differ by the nucleotide used and the number of enzymes involved: three for bacteria and two in APMV. Plants have only one enzyme because UGD and UGER are part of the same protein. (b) Reaction of APMV R141 (or ATCV-1 Z544R) with UDP-α-d-Glc to give UDP-4-keto-α-d-Glc; this substrate is used by two APMV proteins L780 and L136 to synthesize UDP-β-l-Rha and UDP-α-d-Vio4N, respectively. This last product is *N*-acetylated by N-L142, leading to the formation of UDP-α-dVio4NAc. (c) Crystallographic structures of: L136 (PDB 7MFO) in complex with TDP-Vio4N linked as an external aldimine, R141 (PDB 6VL0) in complex with TDP and NAD, and L780 (PDB 7JID) with UDP-β-l-Rha bound; α-helices are colored in red, β-strands in cyan, and loops in gray. The N-L142 model was obtained by homology modeling using *Neisseria gonorrheae* PglB enzyme as a reference. The activities of the enzymes are indicated as acronyms in brackets.

Analysis of the ATCV-1 genome identified the Z544R protein as homologous to UDP-α-d-Glc 4,6-dehydratase (UGD, Table 2),^58^ the first enzyme involved in the production of UDP-β-l-Rha, according to the plant biosynthetic pathway (Figure 4a). Indeed, the ATCV-1 UGD protein preferred UDP-α-d-Glc as a substrate over dTDP-α-d-Glc and converted it to UDP-6-deoxy-4-keto-α-d-Glc, the intermediate transformed into UDP-β-l-Rha by the second enzyme in the pathway, an epimerase and reductase termed UGER (Figure 4a).^161^

ATCV-1 does not encode the second enzyme (UGER) needed to complete the UDP-β-l-Rha synthesis; therefore, the current hypothesis is that the virus encodes its own UGD enzyme to increase Rha production during infection that is brought to completion by using the host encoded UGER enzyme. Furthermore, Z544R is expressed as a late gene product during virus replication. This could be due to the fact that high UGD activity during the initial phases of virus replication is avoided to prevent a reduction of UDP-α-d-Glc availability because this sugar nucleotide serves both as a donor for glucosyltransferases and as a precursor for other sugar nucleotides.^58^

Biochemical characterization of Z544R demonstrated that the protein was cation independent and that its molecular weight was unaffected by the addition of NAD or NADP, in either oxidized or reduced forms, suggesting that the recombinant chlorovirus protein strongly bound the coenzyme (NAD^+^) in the binding pocket when expressed in *E. coli*.^58^ ATCV-1 UGD associated as a dimer, and it had a specific activity of 23 nmol/min/mg of protein and a *K*_m_ for UDP-α-d-Glc of 18.4 ± 3.3 μM at the pH optimum of 7.5. Finally, incubation of the ATCV-1 UGD enzyme at temperatures higher than 20 °C resulted in a progressive loss of activity, and its complete inactivation occurred at 30 °C in 30 min, indicating that the enzyme is thermolabile.^58^

#### 3.1.2 Megavirinae

Looking at the distribution of the glycogenes in the genomes of giant viruses, it appears that *Chlorovirus* and *Mimivirus* genera have a different organization. In PBCV-1, the prototype of the *Chloroviruses*, the glycogenes are mostly scattered throughout the genome, with a majority in the first 100 ORFs, making it difficult to define a specific region responsible for the glycosylation of the viral capsid protein. In contrast, glycogenes in the *Megavirinae* subfamily occur in specific regions of the genome, defining complex clusters of six to as many as 33 genes.^87^ The next sections will describe the sugar nucleotide biosynthetic pathways that have been experimentally validated for *Megavirinae*, together with a few inferred from in silico analysis.

##### 3.1.2.1 APMV Production of UDP-β-l-Rha and UDP-α-d-Vio4NAc

Unlike the chlorovirus ATCV-1, the mimivirus APMV encodes both the UGD and UGER enzymes necessary for UDP-β-l-Rha production (Figure 4b, Table 2).^58^ Bioinformatic analysis of the APMV genome identified R141 as a UGD enzyme, and biochemical assays performed on the recombinant protein expressed in *E. coli* confirmed this prediction.^58^

Compared to ATCV-1 UGD (Z544R, section 3.1.1.1), the binding of the cofactor NADH to R141 appeared less efficient. Indeed, recombinant Z544R copurified with its NADH cofactor, as demonstrated by the fact that it was insensitive to its addition in solution. Recombinant R141 instead did not copurify with the cofactor, as demonstrated by measuring its MW by size exclusion chromatography. With no addition of NADH or NAD^+^, the protein produced three forms at different MWs. However, upon incubation with either NADH or NAD^+^, the lower MW peaks were converted into the major one at ∼100 kDa. Moreover, this experiment established that the protein had a 10-fold better affinity for NADH compared to NAD^+^, and none for NADP^+^ or NADPH. Notably, the apparent MW of 100 kDa exceeded that expected for the dimeric form of the enzyme (2 × 37 kDa), and thus it appears to adopt a higher degree of oligomerization, which was not determined.

Regarding its activity, R141 had maximal activity at pH between 7.5 and 8.5, and it required 100 μM NAD^+^ to transform UDP-α-d-Glc into UDP-6-deoxy-4-keto-α-d-Glc, with a specific activity of 4.2 nmol/min/mg of protein; a 60% feedback inhibition occurred with UDP-β-l-Rha.^58^ Finally, R141 was cation independent, and loss of activity occurred at 30 °C in 30 min, like the analogous enzyme from ATCV-1.

As for UGER, the second enzyme in the UDP-β-l-Rha pathway, the APMV protein L780 had such activity; it had 40% amino acid identity with the C-terminal region of *Arabidopsis thaliana* RHM2 protein, the plant enzyme with the epimerase/reductase activity required to transform UDP-6-deoxy-4-keto-α-d-Glc produced from UGD into UDP-β-l-Rha. Biochemical studies on the recombinant APMV L780 protein revealed that (1) it needed NADPH (and not NADH) as a cofactor with a specific activity for UDP-6-deoxy-4-keto-α-d-Glc of 11 ± 2.5 nmol/min/mg and a *K*_m_ of 183 ± 51 μM; (2) it had maximum activity at pH 7.5, (3) it was cation independent, and (4) it did not lose activity after 30 min incubation at 42 °C. Size exclusion chromatography indicated that L780 MW was 47 kDa, a value that did not fit with the monomeric form of the protein (33 kDa) or its dimer, leaving this as an open point for further studies.

Recent studies have indicated that both R141 and L780 can use either UDP or dTDP-sugars as substrates, in contrast to their bacterial counterparts that specifically require dTDP-sugars. R141 can use either UDP-α-d-Glc or dTDP-α-d-Glc with the same catalytic efficiency;^152^ similarly, L780 used both UDP- and dTDP-6-deoxy-4-keto-α-d-Glc nucleotides, albeit it had a higher catalytic efficiency with the UDP-linked substrate.^155^

Both R141 (PDB 6VLO) and L780 (PDB 7JID) belong to the SDR superfamily, and their structures have been defined by crystallographic studies (Figure 4c).^152,155^ In R141, the residues typically involved in the 4,6-dehydratation reaction are conserved and correspond to ^125^Asp, ^126^Gly, and ^149^Tyr. The overall fold of R141 is comparable to that of the corresponding bacterial enzymes, with some differences: the catalytic ^149^Tyr is ∼12 Å away from the catalytic site, suggesting that the protein undergoes a large conformational change during substrate binding, which is unusual for SDR proteins.

The L780 crystallographic structure had a good match with the *A. thaliana* RHM2 protein, in agreement with the fact that the viral enzyme is bifunctional and has both 3,5-epimerase and 4-reductase activities, different from those from bacteria and fungi, where the same transformation requires two different enzymes. Concerning the catalytic mechanism of L780, comparison of this enzyme with a different sugar nucleotide synthase, the GDP-β-l-fucose synthase from *E. coli*,^162^ led to the identification of ^108^Cys and ^175^Lys as key catalytic residues, as also confirmed by mutagenesis experiments.^155^

The UDP-β-l-Rha and the UDP-α-d-Vio4NAc pathways are interconnected;^58,62^ the intermediate UDP-6-deoxy-4-keto-α-d-glucose produced by R141 and used by L780 to make UDP-β-l-Rha, is also used by L136, a pyridoxal phosphate (PLP) dependent-aminotransferase or transaminase (Table 2), to synthesize UDP-α-d-Vio4N (Figure 4b). The last enzyme in the UDP-α-d-Vio4NAc pathway, is an *N*-acetyl transferase corresponding to the N-terminal domain of L142 (N-L142), a protein with two domains (Table 2).^60^

The two enzymes involved in the UDP-α-d-Vio4NAc biosynthetic pathway, L136 and N-L142, were characterized biochemically, and the three-dimensional structure of L136 was determined by X-ray crystallography (PDB 7MFQ),^151^ while the N-L142 structure was derived by homology modeling studies using the *Neisseria gonorrheae* PglB enzyme as a reference, a transferase able to acetylate the amino function at C-4 of bacillosamine.^60^

L136 is dimeric in the crystal structure, and has the same typical type I fold of the superfamily of aminotransferases (Figure 4c).^151^ L136, like both R141 and L780, accepts both UDP and dTDP-sugars, with similar catalytic efficiencies.^151^ Recombinant L136 maintains its cofactor, PLP, during all the purification procedures, and it uses glutamate as the amino donor for the transaminase reaction, that is not reversible. Glutamine, the amino acid used as donor in bacterial enzymes,^62^ instead inhibits L136 activity.

The reaction product of L136 is UDP-α-d-Vio4N, that in turn is acetylated on the amino group by L142. The N-terminal domain has the acetyltransferase activity, and it uses acetyl-CoA as the donor of the acetyl group.^60^ N-L142 activity was only tested with UDP-α-d-Vio4N, although we speculate that it might also accept a dTDP-nucleotide as found for L136, the previous enzyme in the pathway. N-L142 was predicted to be a homotrimer, with ^136^His playing a key role in the catalysis, as shown by mutagenesis experiments.^60^ In silico analysis of the full length L142, predicted that the second domain (C-L142) was a GT, leading to speculation that it could be involved in the transfer of the viosamine onto its acceptor during the biosynthesis of poly_2, one of the two polysaccharides decorating the fibrils of APMV (section 3.2.2.2).^60^

In conclusion, the existence of UDP-β-l-Rha and UDP-α-d-Vio4NAc biosynthetic pathways seem essential for APMV to build the polysaccharides of its fibrils because these activated sugars are not encoded by the amoeba host. However, it should be noted that the prevalent viosamine form in poly_2 is methylated at *O*-2, implying the existence of a methyltransferase specific for this sugar. Currently, the identity of such an enzyme has only been predicted (section 3.1.2.5)^87^ and awaits experimental verification.

##### 3.1.2.2 APMV UDP-α-d-GlcNAc Pathway

*N*-acetyl-d-glucosamine is present in all domains of life. It is essential for the synthesis of the peptidoglycan layer in bacteria,^163^ of the lipid A moiety of lipopolysaccharides in Gram-negative bacteria,^164^ for the assembly of glycosaminoglycans in eukaryotes,^165^ for the regulation of cellular functions by the reversible *O*-GlcNAc glycosylation of eukaryotic proteins,^166^ and because it is the monosaccharide directly attached to proteins in most of the *N*-linked glycans.^167^

**Figure 5:**
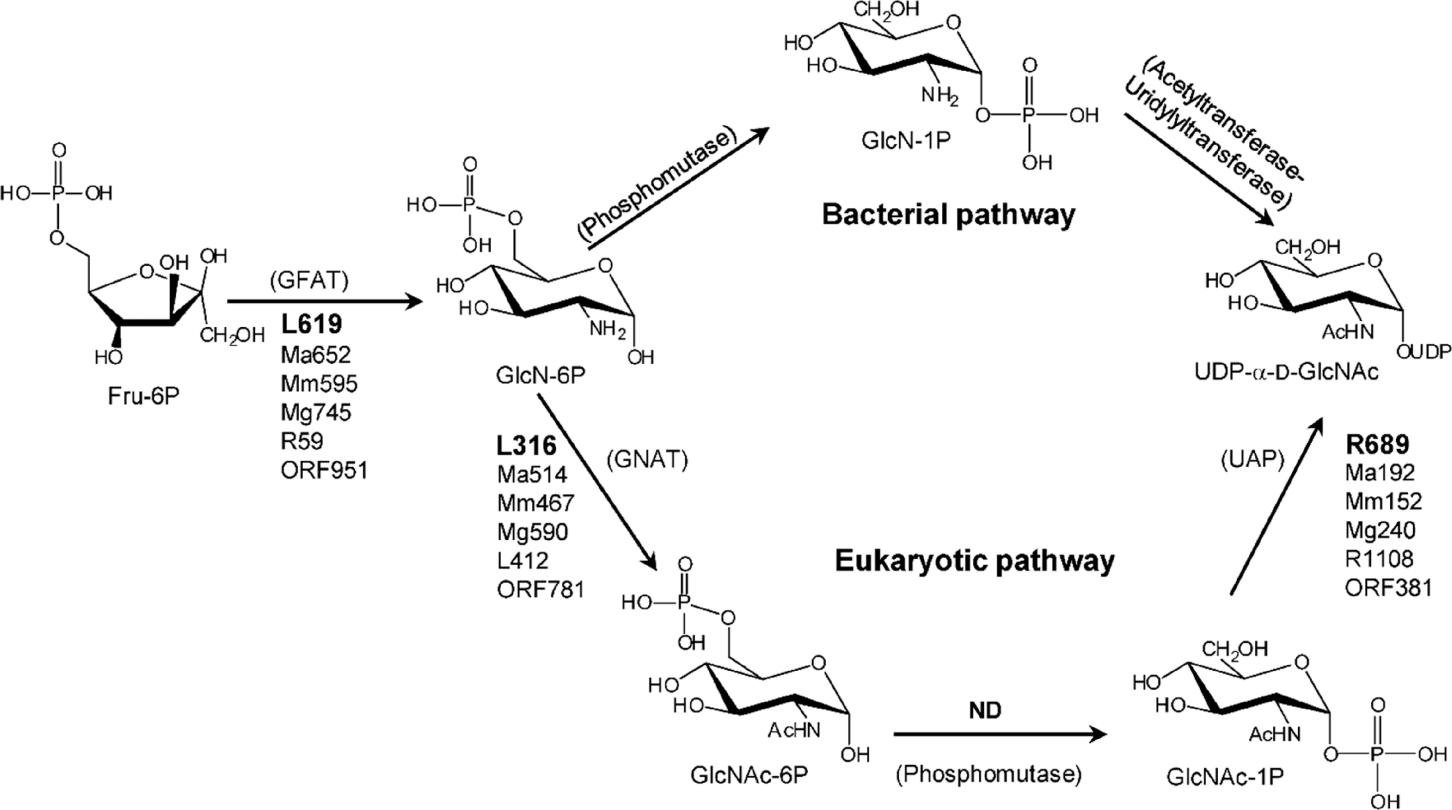
UDP-α-d-GlcNAc biosynthetic pathway. The biosynthesis of UDP-α-d-GlcNAc occurs by different routes, with that from bacteria requiring fewer enzymatic steps. Both pathways start from Fru-6P. Members of the five clades of the *Mimivirus* genus encode all the enzymes in the eukaryotic biosynthetic pattern, except for the GlcNAc-6P to GlcNAc-1P phosphomutase, which is likely encoded by an unknown viral enzyme or by the host (ND: not defined). APMV (prototype of A clade) is the only virus for which the activity of each enzyme (annotated in bold) has been experimentally validated:^61^ L619, L316, and R689. L619 and L316 are close to their eukaryotic counterpart, while R689 appears to be of prokaryotic origin. The activities of the enzymes are indicated in parentheses. As for the representatives of the other clades, the genes involved in this pathway have been assigned by in silico analysis,^87^ and they are listed in the following order: clade B, Moumouvirus australiensis (Ma) and Moumouvirus maliensis (Mm); clade C, Megavirus chilensis (Mg); clade D, Tupanvirus deep ocean (L or R depending on the coding sense of the gene); clade E, Cotonvirus japonicus (ORF).

The biosynthesis of d-GlcNAc varies between organisms and the APMV pathway has a combination of prokaryotic and eukaryotic traits. Three of the four enzymes required for UDP-α-d-GlcNAc production were identified and experimentally characterized in APMV (Table 2).^154^ The phospho-mutase that converts GlcNAc-6P into GlcNAc-1P is the only enzyme not encoded in the genome. Therefore, it is possible that either APMV encodes an unidentified enzyme or it uses a host enzyme to complete the synthesis of GlcNAc-1P (Figure 5).

The first enzyme in the pathway is L619 (606 aa), a glutamine-fructose 6-phosphate transaminase (GFAT) that converts the Fru-6P to GlcN-6P, using glutamine as an amine donor (Figure 5). This enzyme has a N-terminal domain with glutaminase activity, while the C-terminal domain is an isomerase. The conversion of Fru-6P to GlcN-6P occurs in two independent steps. The first step involves the catalytic activity of the C-terminal domain that converts Fru-6P into Glc-6P, which in turn, is transformed into GlcN-6P by the transaminase activity located at the N-terminal domain of the enzyme. This enzyme is well conserved among other members of the *Mimivirus* genus; it has 61% identity with a Moumouvirus species GFAT (AEX62494), and 59% identity with Megavirus chilensis Mg745 protein, while it has some limited similarity (∼37%) with those from some protista, including its host *Acanthamoeba castellani*. In contrast to the eukaryotic enzyme, the viral enzyme is shorter and lacks an insertion of 40–70 aa between the N- and C-terminal regions, as also happens for the bacterial enzymes.^168^ However, L619 does not exhibit a feedback inhibition by the final product UDP-α-d-GlcNAc, a trait common with the bacterial enzymes. Of note, GFAT has also been found in several chloroviruses (section 3.3.1), where this enzyme seems necessary for some chloroviruses to increase the GlcNAc supply required for the biosynthesis of hyaluronic acid and/or chitin during the infection process (section 3.3.1).^11,146^

The second reaction in the pathway to synthesize UDP-α-d-GlcNAc is catalyzed by L316 (148 aa), a GlcN-6P *N*-acetyltransferase (GNAT) that adds an acetyl group from the acetyl-CoA to the free amino group, leading to GlcNAc-6P (Figure 5). L316 sequence aligns with putative acetyl transferases encoded by other *Mimivirinae* members, it has 57% identical residues with a Moumouvirus species GNAT (YP_007354484) and 56% with the Megavirus chilensis Mg590 (Figure 5). Its closest homologues in cellular organisms are the GNAT proteins from *Trichomonas vaginalis* G3 (41% identity, XP_001303745) and from the archaean *Candidatus nitrosopumilus* (50% identity, ZP_1039775). L316 acts on both GlcN-1P and GlcN-6P with a marked preference (5-fold) for GlcN-6P, in agreement with the results reported for GNAT proteins of eukaryotic origin and contrary to bacterial enzymes that only act on GlcN-1P due to a different biosynthetic pathway (Figure 5).^169^

The last step in the pathway is the addition of a uridyl moiety from UTP to the phosphorylated sugar to generate UDP-α-d-GlcNAc, carried out by R689 (255 aa). This enzyme is a UDP-α-d-GlcNAc pyrophosphorylase (UAP) that belongs to the GTA-type GT superfamily with some resemblance to the N-terminal domain of *E. coli* GlmU. Its closest homologues are in other members of the *Megavirinae* subfamily, with 66% identity with the Megavirus chilensis Mg240 protein and 68% identity with the Moumouvirus protein (YP_007354151), albeit here the comparison was limited to the 172 amino acids of this smaller protein. The closest homologues to R689 are the N-terminal domains of Cyano- and Proteobacteria GlmU proteins, with 36 and 38% identical residues, respectively. Biochemical assays indicated that R689 had both uridyltransferase and pyrophosphorylase activities and that it was Mg^2+^ dependent. The highest efficiency was observed when using UTP as donor nucleotide and GlcNAc-1P as the substrate both for the forward and reverse reaction. The reaction occurred with lower efficiency when Glc-1P was used as a substrate or when dTTP and GTP were used as nucleotide donors, while no reaction was observed with ATP and CTP.

The identification of three of the enzymes involved in the UDP-α-d-GlcNAc pathway in APMV was unexpected, as this monosaccharide is already synthesized by the host, contrary to Rha and Vio4NAc (section 3.1.2.1). We hypothesize that APMV synthesizes this sugar nucleotide to be independent of the host supply, especially during the replication cycle when large amounts are required for the synthesis of the glycans that decorate the fibrils (section 3.2.2).

Regarding the strategy adopted by the virus, it should be noted that it follows the eukaryote route,^170^ although it uses enzymes that have different origins as denoted by the fact that the closest match to R689 is a bacterial protein, while the other two seem to be of eukaryotic origin.^169^ These results lead to an intriguing question on the evolutionary history of this pathway in relation to that of the cellular world.

Homologues to the APMV enzymes used to produce UDP-α-d-GlcNAc have been identified in members of the other clades (Figure 5), during investigation of the clustering of their glycogenes (section 3.1.2.5). Indeed, GFAT is encoded by Moumouvirus australiensis (Ma652), Moumouvirus maliensis (Mm595), Tupanvirus deep ocean (R59), and *Cotonvirus japonicum* (ORF951); GNAT occurs in *Moumouvirus australiensis* (Ma514), Moumouvirus maliensis (Mm467), Tupanvirus deep ocean (L412), and Cotonvirus japonicum (ORF781); UAP is found in Moumouvirus australiensis (Ma192), Moumouvirus maliensis (Mm152), Tupanvirus deep ocean (L1108), and Cotonvirus japonicum (ORF381).

Notably, three of the four genes involved in the UDP-α-d-GlcNAc pathway are conserved in all members of the *Mimivirus* genus. These genes are scattered along the genome and not clustered together as found for those involved in fibril synthesis and their glycosylation in clades A–C or clades D and E, for which experimental data are not available yet (section 3.1.2.5).^87^

##### 3.1.2.3 UDP-6-deoxy-β-l-HexNAc Pathway in Megavirus chilensis

**Figure 6:**
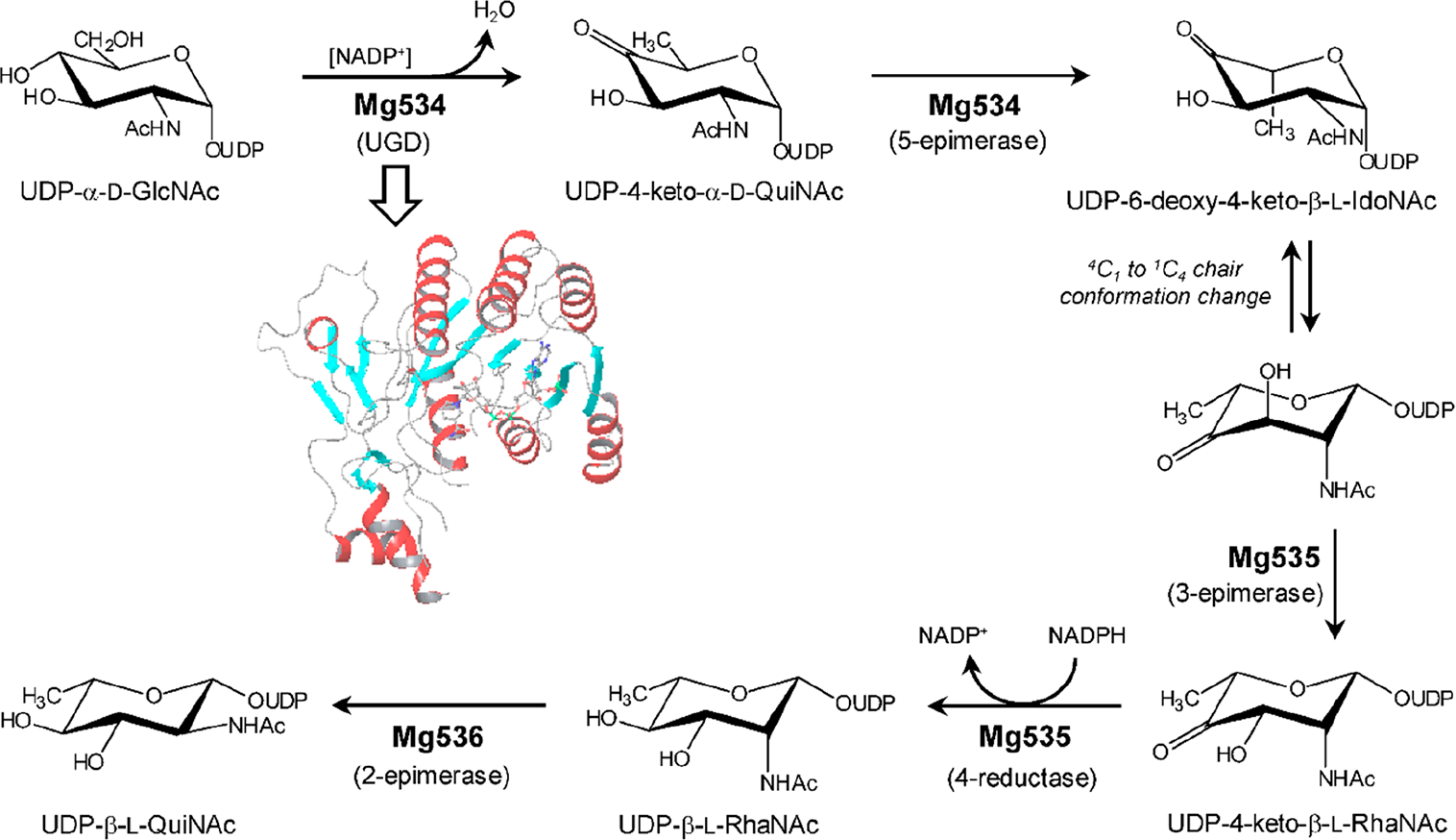
Biosynthetic pathway encoded by Megavirus chilensis to produce two UDP-2,6-dideoxy-β-l-HexNAc. UDP-β-l-RhaNAc is produced by four reactions carried out by two bifunctional enzymes, Mg534 and Mg535, both resembling prokaryotic enzymes. The crystallographic structure of Mg534 is reported and colored according to its secondary structure elements (α-helices in red, β-sheets in cyan, and loops in gray). UDP-β-l-QuiNAc is produced from UDP-β-l-RhaNAc by C-2 epimerization promoted by Mg536. The activities of Mg534 and Mg535 have been experimentally validated, while that of Mg536 was predicted in silico, and it is supported by the monosaccharide analysis of Megavirus chilensis fibrils.^87^

Analysis of the Megavirus chilensis genome revealed the presence of a gene cluster with several ORFs potentially involved in glycosylation, with three of them (*mg534*, *mg535*, and *mg536*) involved in the production of two different sugar nucleotides (Figure 6, Table 2). The protein products of two of them have been characterized (Mg534 and Mg535), and they encode a functional pathway for UDP-β-l-RhaNAc synthesis.^61^ Mg536 was predicted to produce UDP-β-l-QuiNAc, and its activity was later demonstrated by the finding of QuiNAc when the monosaccharide composition of the virion was determined (section 3.1.2.5).^87^ Notably, both RhaNAc and QuiNAc were sugars previously found only in the bacterial world, and their production begins with UDP-α-d-GlcNAc, in turn also synthesized by Megavirus chilensis (section 3.1.2.2).

The first enzyme in the UDP-6-deoxy-HexNAc pathway, Mg534 (323 aa), has two catalytic activities. Mg534 first performs the 4,6-dehydration of UDP-α-d-GlcNAc using a NADP^+^ cofactor like UGD-type enzymes, then the enzyme epimerizes C-5 in the intermediate to give UDP-6-deoxy-4-keto-β-l-IdoNAc (Figure 6). The three-dimensional structure of Mg534 (PDB 4TQG) revealed two subdomains, a Rossman-like fold binding NADP and a smaller domain for the substrate binding. The protein is found as a hexamer made of a trimer of dimers, and it belongs to the SDR superfamily, in agreement with the presence of a catalytic triad containing the motif ^140^Tyr-X-X-X-^144^Lys (with X any amino acid), close to a disordered loop (aa 173–195), and for this reason is not visible in the crystallographic structure.^61^

The first reaction product of Mg534, UDP-4-keto-α-d-QuiNAc is epimerized at C-5 to give UDP-6-deoxy-4-keto-β-l-IdoNAc, the substrate of Mg535 (270 aa), a NADPH dependent enzyme that yields UDP-β-l-RhaNAc following a two-step mechanism. First, UDP-6-deoxy-4-keto-β-l-IdoNAc is epimerized at C-3, then the carbonyl function is reduced to produce the final product (Figure 6).

Regarding the Mg535 reaction mechanism, it can be compared to enzymes involved in NDP-β-l-Rha synthesis. In plants and viruses, the RmlD reductase domain and the 3,5 double epimerase are in the same protein, while in bacteria, these are different proteins (Figure 4a). At odds with plant and other giant viruses enzymes such as L780 (section 3.1.2.1), Mg535 maintains the reductase Rmld-like domain and only the C-3 epimerase activity. This enzyme lacks the C-5 epimerase activity as the C-5 stereochemistry is already inverted by the previous enzyme in the pathway, Mg534 (Figure 6). Interestingly, studies on a different sugar-nucleotide manipulating enzyme, a GDP-β-l-Fuc synthase, enabled the identification of the catalytic residue involved in the C-3 epimerization of the intermediate, a cysteine conserved in Mg535, despite the low sequence homology between the two proteins.^162^

Regarding Mg536, it is annotated as a UDP-α-d-GlcNAc 2-epimerase, but it also displays 50% and 47% identity, respectively, with WbvD from *Vibrio cholerae* O37,^171^ and WbjD from *Pseudomonas aeruginosa*,^172^ two proteins involved in C-2 epimerization of 2-acetamido-2,6-dideoxy-l-hexoses. This finding suggested that Mg536 could act on UDP-β-l-RhaNAc produced by Mg535 to transform it into UDP-β-l-QuiNAc (Figure 6). This hypothesis has been confirmed by analyzing the monosaccharide content of the virion,^87^ validating the tentative bioinformatic prediction of the original study (section 3.1.2.5).^61^

##### 3.1.2.4 Putative Kdo Biosynthetic Pathway in Cafeteria Roenbergensis Virus and Fadolivirus

**Figure 7:**
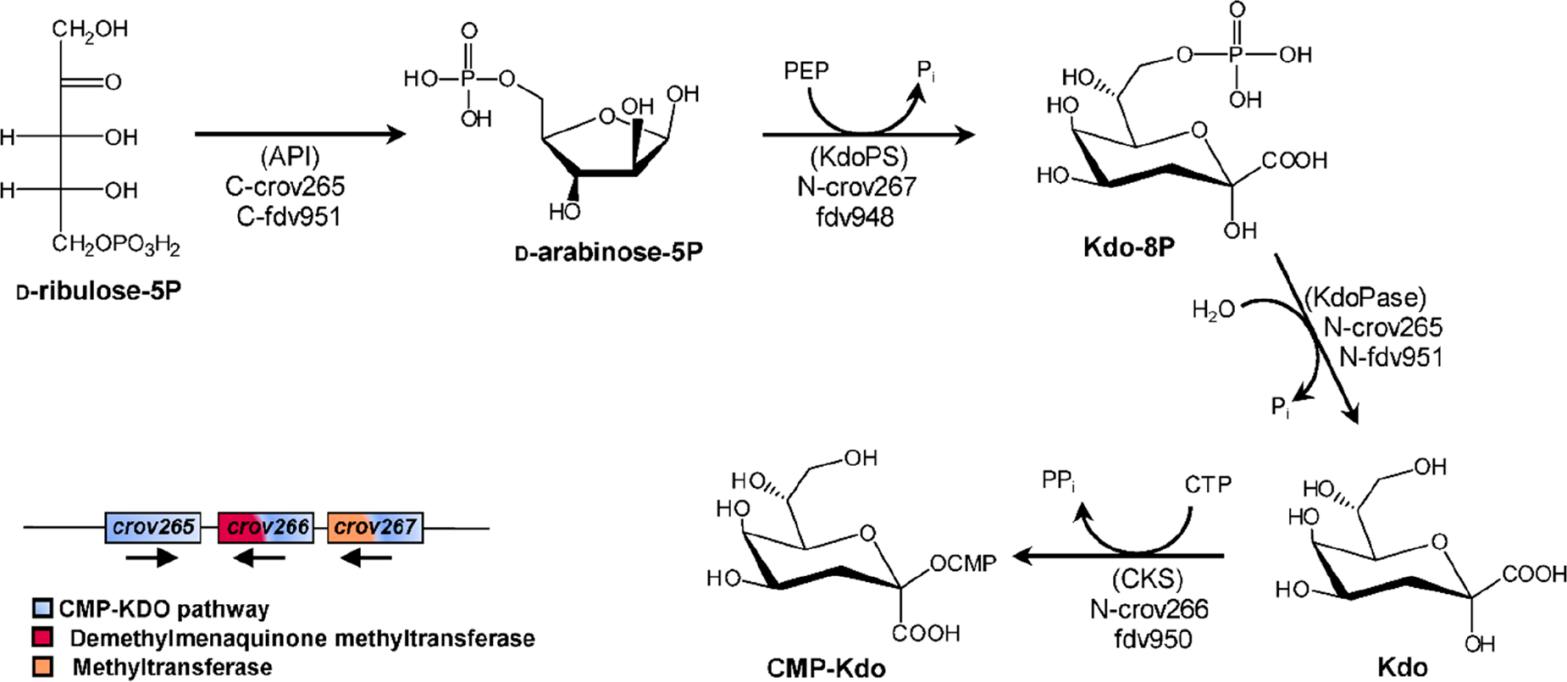
Putative Kdo biosynthetic pathway in CroV and Fadolivirus (*Mimiviridae*family). The Kdo pathway follows the same path in bacteria and plants, and in silico analysis suggests that these two giant viruses encode all the enzymes required to produce this monosaccharide. The arrangement of the genes is given for CroV only (lower left part of the figure), and the arrows (in black) indicate the direction of the coding strand.

Fischer and coauthors have studied the genomic features of CroV, and described a 38 kb gene cluster involved in carbohydrate metabolism.^59^ This cluster has 34 ORFs (crov242–275), of which 14 show significant homology with bacterial enzymes; seven are clearly related to carbohydrate metabolism. Interestingly, this cluster is predicted to encode proteins involved in the biosynthesis of Kdo (Figure 7), an eight carbon atoms sugar that is essential for the construction of the LPS in Gram-negative bacteria and which also occurs in plant pectins.^164^ The Kdo pathway is well conserved in bacteria and plants (Figure 7).^173,174^ First, d-ribulose-5-phosphate (Ru5P) is isomerized to d-arabinose-5-phosphate (A5P) via an isomerase (API). Then a synthase (KdoPS) condenses A5P with phosphoenolpyruvate (PEP) to give Kdo-8P, which is hydrolyzed to free Kdo by a phosphatase (KdoPase). Finally, the free Kdo reacts with CTP to produce CMP-β-Kdo, the activated form used by GTs, in a reaction catalyzed by CMP-Kdo synthetase (CKS). The identity of the four bacterial enzymes, together with their plant counterparts, is known, albeit the nature of the phosphatase involved in the production of free Kdo in plants is still unknown.

The predicted Kdo biosynthetic pathway in CroV involves three proteins: crov265, crov266, and crov267. The C-terminal domain of crov265 matched with API and catalyzed the first biosynthetic step of Kdo synthesis; the second enzyme in the pathway was assigned to the N-terminal domain of crov267 due to its resemblance to the KDO-8P synthase (KdoPS). The third reaction is catalyzed by N-terminal domain of crov265, which has the Kdo-8P phosphatase (KdoPase) activity. Finally, the fourth step is the activation of Kdo as a CMP nucleotide, and this activity was tentatively assigned to the N-terminal domain of crov266, although phylogenetic analysis associated this putative protein to a *N*-acylneuraminate cytidylyltransferase (CMP-NeuAS) and not to CKS (Figure 7). Notably, all of the genes involved in the Kdo pathway encode bifunctional enzymes. Both domains of crov265 are involved in different steps in the synthesis of the Kdo. On the other hand, the C-terminal of crov267 is predicted to encode a dTDP-6-deoxy-l-hexose 3-*O*-methyltransferase, while the C-terminal domain of crov266 is annotated as a demethylmenaquinone methyltransferase.^59^ Currently, none of the enzymatic activities of this putative Kdo pathway have been validated, nor have the structure of the CroV glycans been determined, which would support the in silico prediction.

Interestingly, CroV is not the only giant virus that has been predicted to encode the Kdo pathway. Fadolivirus, a member of the *Mimiviridae* family but in a different subfamily (Table 1), has all the genes necessary to produce this monosaccharide. Indeed, the protein with ID QKF94409 (Fadolivirus_1_951, or fdv951 for simplicity) is annotated as a bifunctional protein with 3-deoxy-d-*manno*-octulosonate 8P phosphatase/sugar isomerase activity at the N- and C-terminal domains, respectively; assuming that the sugar isomerase domain performs the API function, this gene would have the same predicted function as crov265 (Figure 7). The protein ID QKF94408.1 (fdv950) is annotated as a 3-deoxy-d-*manno*-octulosonate cytidylyltransferase (CKS), while protein ID QKF94406 (fdv948) is annotated as a 3-deoxy-8-phosphooctulonate synthase (KdoPS). Regarding Fadolivirus, information about the putative Kdo pathway does not go beyond in silico analysis,^38^ while the analysis of CroV enzymes have examined the potential evolutionary source of the pathway, i.e., from plants or bacteria. However, the authors could not reach a definitive conclusion about the origin of this gene cluster. They speculated that it could have been acquired from a bacterium, although they noted that some of these proteins (crov267 and crov265) have features common to both plants and bacteria. Thus, the prediction that CroV and Fadolivirus encode Kdo biosynthetic enzymes leads to many questions about how the viruses use this monosaccharide and about the origin of the enzymes.

##### 3.1.2.5 Mimivirus Glycogenes Are Organized in Clade-Specific Clusters

**Figure 8:**
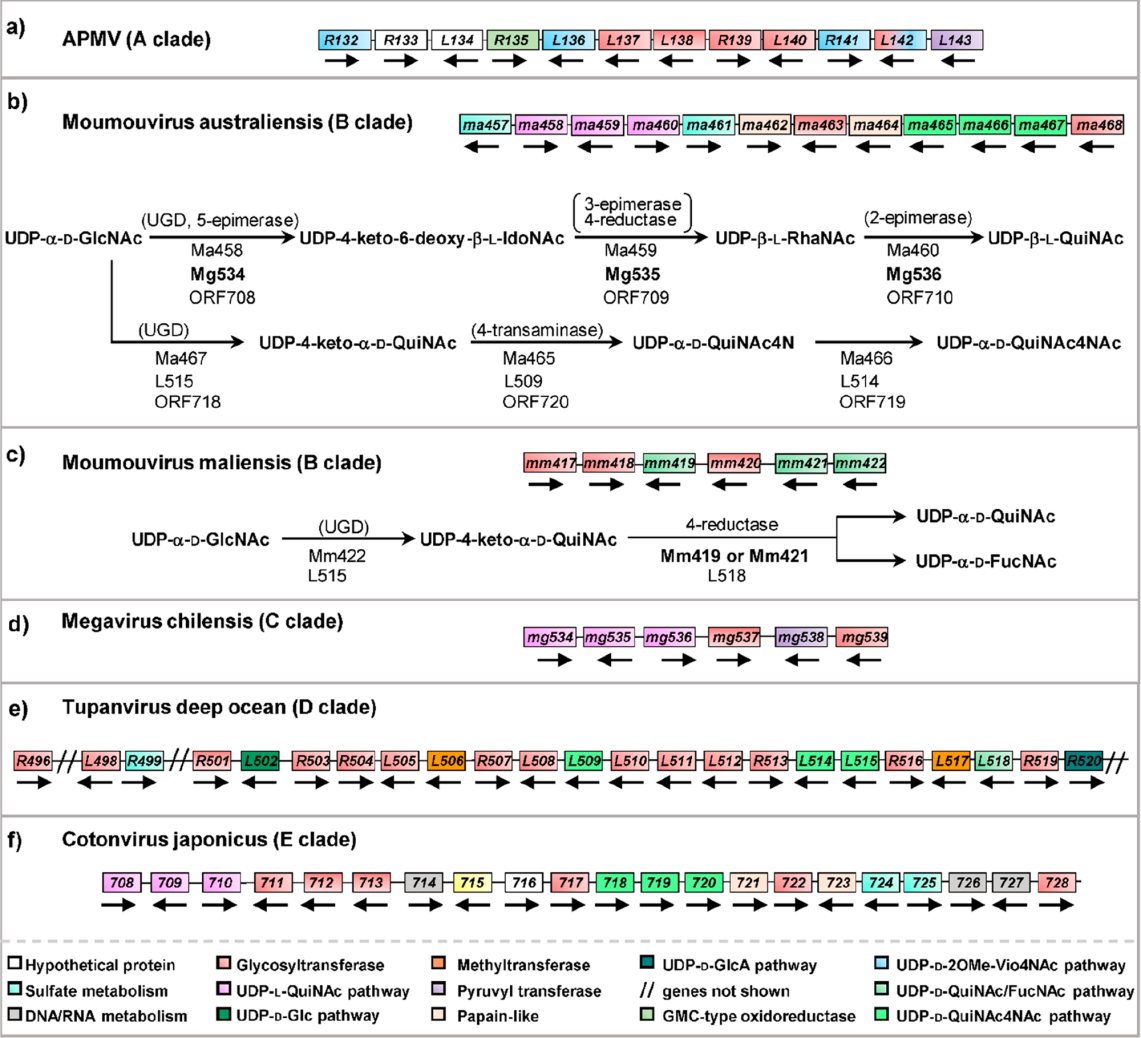
Organization of glycosylation genes in the *Mimivirus* genus. Genes in bold indicate that their activity has been experimentally validated, otherwise they are written in plain text. The activity of each gene is color coded as reported in the legend or is explicitly mentioned (in brackets) in the schemes of the reactions. The arrows below the gene names indicate the direction of the coding strand. Importantly, sugar fibril composition has only been determined experimentally for the viruses in the A, B, and C clades; for the other clades (D and E), sugar composition is predicted from in silico analysis. (a) Clade A: APMV glycogene cluster (12 genes) corresponds to UDP-β-l-Rha and UDP-α-d-Vio4NAc production, along with methyl-, pyruvyl-, and glycosyltransferases and other enzymes either manipulating sugars or involved in the construction of the fibrils. (b) Clade B: Moumouvirus australiensis glycogene cluster (12 genes) corresponds to the biosynthetic pathway for l-QuiNAc and d-QuiNAc4NAc, and several GTs and other sugar manipulating enzymes including those of sulfate metabolism. (c) Clade B: Moumouvirus maliensis cluster (six genes) corresponds to d-QuiNAc and d-FucNAc production: notably, Mm419 and Mm421 are two 4-reductases, and it is not clear which one produces d-QuiNAc or d-FucNAc. (d) Clade C: Megavirus chilensis cluster (six glycogenes) corresponds to l-QuiNAc and l-RhaNAc production; notably the activity of Mg534 and Mg535 has been determined experimentally, while that of Mg536 was only predicted. (e) Clade D: Tupanvirus deep ocean cluster (33 genes in total, only the 25 related to carbohydrate metabolism are displayed) is predicted to correspond to the biosynthesis of d-QuiNAc4NAc and to that of a yet unidentified 6-deoxy-d-HexNAc (either d-QuiNAc or d-FucNAc) along with several other glycogenes. (f) Clade E: Cotonvirus japonicus cluster (21 genes) is predicted to correspond to l-QuiNAc and d-QuiNAc4NAc production, along with enzymes involved in sulfate metabolism and others.

A recent study on several members of the *Mimivirus* genus (Table 3) found that most of the glycogenes were organized in clusters whose size is clade-dependent.^87^ The study also reported that each clade uses specific sugars, with some clades having few pathways in common (Table 3, Figure 8). This study also analyzed the genome of 55 fully sequenced viruses in five clades and combined the results of the bioinformatic predictions with the experimental data, either acquired in the same work or taken from previous studies.^87^ The protein sequences of well-annotated glycogenes were used as input to search for homologous proteins in the genome of each virus. Multiple alignments were performed to assess the conservation level of the active sites and consequently the functionality of the enzymes. The analytical results for each clade are summarized at the end of this section, while the main conclusions of the study follow hereafter.

**Table 3:** Number of Viruses in the *Mimivirus* Genus Used to Define the Glycogene Clusters of Each Clade, Along with Information Regarding the Type of Sugars Encoded and the Size of the Clusters^a^,^87^

clade	no. of viruses	prototype	cluster (no. of genes)	monosaccharide marker
**A**	28	APMV	12	l-Rha, d-Vio4NAc
**B**	7	Moumouvirus australiensis^b^	12	l-RhaNAc, l-QuiNAc, d-QuiNAc4NAc
		Moumouvirus maliensis	6	d-QuiNAc, d-FucNAc
**C**	17	Megavirus chilensis	6	l-RhaNAc, l-QuiNAc
**D**	2	Tupanvirus deep ocean	33	d-QuiNAc or d-FucNAc, d-QuiNAc4NAc, d-GlcA
**E**	1	Cotonvirus japonicus	21	l-RhaNAc, l-QuiNAc, d-QuiNAc4NAc

a Monosaccharide composition is based on experimental data, except for clades D and E, where it is provided by *in silico* analysis.

b Moumouvirus australiensis is considered as an outlier of the clade based on the characteristics of its glycogene cluster. See text for discussion.

Overall, the data suggest that sugar composition in the genus *Mimivirus* is clade-specific, and it is governed by complex gene clusters (Figure 8) responsible for the synthesis of sugars mostly restricted to the bacterial world. How these gene clusters originated is an open question. Notaro and coauthors proposed that the cluster organization of the glycogenes resembles the operon system in the bacterial world. However, in these giant viruses, most of the glycogenes have their own promoter, thus placing them between bacteria and eukaryotes. Moreover, the GC content of these glycogenes and the sequence of the promoter regions are similar to the core genes (genes conserved in members of the family), suggesting that giant viruses might have acquired their own glycosylation machinery before the evolution of the first eukaryotes. Finally, the existence of this great variability in the glycosylation machinery of the different clades suggests that glycans contribute to the fitness of the viruses in the environment, where they have to compete with bacteria and other giant viruses for the same host.^87^

###### Clade A

Analysis of this clade involved 28 different strains, with APMV as the prototype. This choice was made as it is the virus for which the function of many of the gene products have been experimentally demonstrated^58,60,62^ and found to be consistent with the fibril glycans (section 3.2.2.2).

Originally, the APMV gene cluster included nine genes:^60^ those involved in the biosynthesis of UDP-α-d-Vio4NAc and UDP-β-l-Rha (*R141*, *L136*, *N-L142*, sections 3.1.2.1), a putative pyruvyl transferase (*L143*), five putative GTs (*L137*, *L138*, *R139*, *L140*, and C-*L142*), and *R135*, a GMC-type oxidoreductase known to be a component of the fibrils, that in this virus are glycosylated (section 3.2.2.2).^45^ The inclusion of three other genes in the cluster was prompted by the finding that Vio4NAc, a component of the polysaccharide of the fibrils, was partially methylated at *O*-2. Accordingly, *R132* was identified as a SAM-dependent methyltransferase and suggested to be responsible for this modification (Figure 8a), thus expanding the original cluster from 9 to 12 genes by including *L133* and *L134*, both corresponding to hypothetical proteins (Figure 8a).^87^

Importantly, the *Mimivirus* 12 gene-cluster was preserved inside the A clade, and it was not shared by members of other clades, suggesting that Rha and Vio4NAc are the markers for this clade (Table 3, Figure 8a). Other glyco-related genes were scattered elsewhere in the genome: GlcNAc biosynthesis (section 3.1.2.2) and *L780*, which completes UDP-β-l-Rha production that is started by *R141* (section 3.1.2.1).

###### Clade B

Analysis of this clade included seven viruses, and the composition of their fibrils was determined for two, Moumouvirus australiensis (Figure 1g) and Moumouvirus maliensis (Figure 1h). These viruses have different fibril morphology (thick and compact for maliensis and sparse for australiensis) and differences in their respective glyco-related clusters.

Moumouvirus australiensis fibrils have d-GlcNAc, l-QuiNAc, and d-QuiNAc4NAc and the biosynthetic pathways for the corresponding sugar nucleotides were assigned in silico. As for UDP-β-l-QuiNAc (Figure 8b), UDP-α-d-GlcNAc is converted to 6-deoxy-4-keto-β-l-IdoNAc by the bifunctional enzyme Ma458 (4,6-dehydratase, 5-epimerase), and the intermediate is epimerized at C-3 and reduced at the 4-keto function by Ma459. The resulting UDP-β-l-RhaNAc is finally epimerized at C-2 by Ma460 to produce UDP-β-l-QuiNAc. For UDP-α-d-QuiNAc4NAc, the predicted biosynthesis starts from UDP-α-d-GlcNAc, with C-4 oxidation catalyzed by Ma467, followed by transamination of the C-4 keto function promoted by Ma465. Finally, Ma466 attaches the acetyl group to the newly generated amino group at C-4 (enzymes characteristics are reported in Table 2).

The Moumouvirus australiensis gene cluster includes 12 genes, with 10 having glyco-related activity. Two of the encoded enzymes (Ma457 and Ma461) are involved in sulfate metabolism, suggesting that the sugars could be decorated with a sulfate group. Others include genes encoding for single (Ma463) or multidomain GTs (Ma468) and yet more related to sugar nucleotide biosynthesis (Ma458, Ma459, Ma460, Ma655, Ma466, and Ma467), whose functions are yet to be identified. The other two genes included in the cluster (Ma462 and Ma464) have papain-like domains (Figure 8b). The glycogenes defined for Moumouvirus australiensis are not shared by the other members of the clade, making this virus an outlier of the B clade. Moreover, the Moumouvirus australiensis glycosylation gene cluster had features common to those of clades C, D, and E (Table 3). Indeed, Moumouvirus australiensis has pathways to make the same sugars as members of clade E (l-RhaNAc, l-QuiNAc, and d-QuiNAc4NAc) along with the enzymes involved in sulfate metabolism, while it has l-RhaNAc and l-QuiNAc in common with clade C and d-QuiNAc4NAc with clade D (Table 3, Figure 8b).

In Moumouvirus maliensis (Figure 8c), the fibrils have d-QuiNAc and d-FucNAc, and only traces of d-GlcNAc. The synthesis of UDP-α-d-QuiNAc and UDP-α-d-FucNAc are interconnected, as they begin with the conversion of the UDP-α-d-GlcNAc into UDP-4-keto-α-d-QuiNAc by Mm422, a 4,6-dehydratase enzyme (UGD). This intermediate is converted to UDP-α-d-QuiNAc or to its C-4 epimer UDP-α-d-FucNAc, depending on the stereospecificity of the 4-reductase enzymes involved. The cluster has two enzymes with this predicted activity, Mm419 and Mm421, but in silico analyses could not attribute the precise activity to either of them due to their strong homology (enzymes characteristics are reported in Table 2). Next to the genes involved in the production of d-QuiNAc and d-FucNAc, the cluster includes three GTs (Mm417, Mm418, and Mm420), for a total number of six genes (Figure 8c). Importantly, the Moumouvirus maliensis six-gene cluster is conserved in all the members of this clade (except Moumouvirus australiensis) so that d-QuiNAc and d-FucNAc can be considered as the markers of clade B, with one of them also being encoded by *Tupanvirus* (clade D, Figure 8e).

###### Clade C

Analysis of this clade included 17 viruses and identified a cluster of six well-conserved genes (Figure 8d). Megavirus chilensis served as prototype for the clade, and analysis of the fibrils detected d-GlcNAc, l-RhaNAc, 4OMe-l-RhaNAc, and l-QuiNAc. The identification of RhaNAc was in agreement with the pathway previously described for the nucleotide, UDP-β-l-RhaNAc (section 3.1.2.3, Figures 5 and 7d),^154^ while that of l-QuiNAc corroborated the hypothesis that the gene *mg536* could encode a 2-epimerase (section 3.1.2.3, Figures 5 and 7d).^61^ The region next to the genes encoding UDP-β-l-RhaNAc and UDP-β-l-QuiNAc (*mg534*, *mg535*, and *mg536*) contained three other genes involved in sugar manipulation: one putative single GT domain (*mg537*), one with three GT domains (*mg539*), and a putative pyruvyl transferase (*mg538*). Therefore, the glycosylation gene cluster of Megavirus chilensis consists of six genes (from *mg534* to *mg539*) as for Moumouvirus maliensis, although it lacks the transferase that methylates *O*-4 of RhaNAc (Figure 8d). This methyltransferase is yet to be identified. l-RhaNAc and l-QuiNAc are considered to be markers of clade C.

###### Clade D

Analysis of this clade identified a cluster of 33 conserved genes for the two members analyzed (Table 3). Tupanvirus deep ocean was used as a reference, and the cluster corresponds to a 49 kb DNA region made of 25 genes involved in carbohydrate metabolism (Figure 8e). Within this cluster, the biosynthetic pathway of UDP-β-d-QuiNAc4NAc (*L515*, *L509*, and *L514*, Figure 7b,e) was identified along with that for a UDP-6-deoxy-α-d-HexNAc (*L515* and *L518*, Figures 7c,e), either UDP-α-d-QuiNAc or UDP-α-d-FucNAc. The identity of the HexNAc could not be predicted because L518, an enzyme predicted as a 4-reductase, had a tight match with both Moumouvirus maliensis Mm419 and Mm421 (Figure 8c), whose stereospecificity is undetermined.

Interestingly, Tupanvirus encodes a putative UDP-α-d-Glc-dehydrogenase (UGDH) able to convert UDP-α-d-Glc to UDP-α-d-GlcA (R520), and Glc-1P-uridyltransferase (N-terminal domain of L502) that produces UDP-α-d-Glc from Glc-1P, an additional tool to manipulate the host cell glycosylation machinery. Regarding the GTs, this virus encodes 11 single-domain GTs (L498, R501, R503, R504, L505, R507, L511, L512, R513, R516, R519), and two others that are predicted to be multidomain proteins (R496 and L510), with four and two GT domains, respectively (Figure 8e). Of the remaining genes, there were methyltransferases (*L506* and *L517*), a SAT/APS kinase involved in sulfate metabolism (*R499*), six ORFans (*R497*, *R500*, *R522*, *R524*, *L525*, *L526*), a putative bifunctional glutamate/proline tRNA-synthetase (*L521*), and a histidine tRNA synthetase (*L523*).

Notably, Tupanvirus deep ocean encoded the highest number of GTs within the *Megavirinae* subfamily, suggesting a complicated glycosylation pattern for its fibrils. Indeed, an intriguing question is whether the fibrils in the tail and in the capsid (Figure 1b,c) have the same glycans or if these are different depending on which part of the virion they decorate. Finally, the monosaccharide markers of clade D, based on in silico analyses are d-QuiNAc4NAc, d-QuiNAc (or d-FucNAc), and d-GlcA (Table 3).

###### Clade E

This clade has only one member, Cotonvirus japonicus, for which no experimental data are available on its carbohydrate composition. In silico analyses identified a cluster of 21 genes (Figure 8f) having some analogies with that of Moumouvirus australiensis (Figure 8b). Indeed, it encodes a putative biosynthetic pathway for UDP-β-l-QuiNAc (ORFs *708*, *709*, and *710*) and UDP-α-d-QuiNAc4NAc (ORFs *718*, *720*, and *719*), along with enzymes involved in sulfate metabolism (ORFs *724* and *725*) and two papain-like proteins (ORFs *721* and *723*) that are not related to glycosylation. Contrary to Moumouvirus australiensis, Cotonvirus encodes a higher number of GTs, six in total: ORFs *711*, *712*, *713*, *717*, *722*, and *728*. Finally, this cluster also has genes involved in DNA/RNA metabolism (ORFs *714*, *726*, and *727*) and one hypothetical protein (ORF *716*). Regarding the sugars that can be considered as markers of the clade, the assignment of l-RhaNAc, l-QuiNAc, and d-QuiNAc4NAc are tentative because they rely on a unique strain (Table 3).

### 3.2 Glycans Structures

#### 3.2.1 Chloroviruses

In 1993, the MCP (called Vp54) of the prototype NC64A chlorovirus PBCV-1 was isolated,^175^ and the initial chemical data suggested that it was glycosylated with an unusual glycan due to the presence of different types of sugars, namely Ara, Gal, Glc, Rha, Man, and Xyl. This was confirmed by the fact that classic procedures used to cleave *N*-linked glycans from the glycoproteins, such as the use of endoglycosidase PNGase F, did not remove the glycan from Vp54. This assumption was later supported in 2002 by crystallographic studies^176^ that detected four *N*-linked oligosaccharides attached to asparagine residues located in atypical sequons, none were the typical sequence NX(T/S) (with X being any amino acid but proline) used in all forms of life. Regarding the structure of the Vp54 glycans from the crystallographic study, the information was not complete and was limited to the sugar units next to the protein backbone. However, at that time, the interpretation of the data only considered the structure typical for the eukaryotic *N*-linked glycans because the structure of these glycans was still unknown.

**Figure 9:**
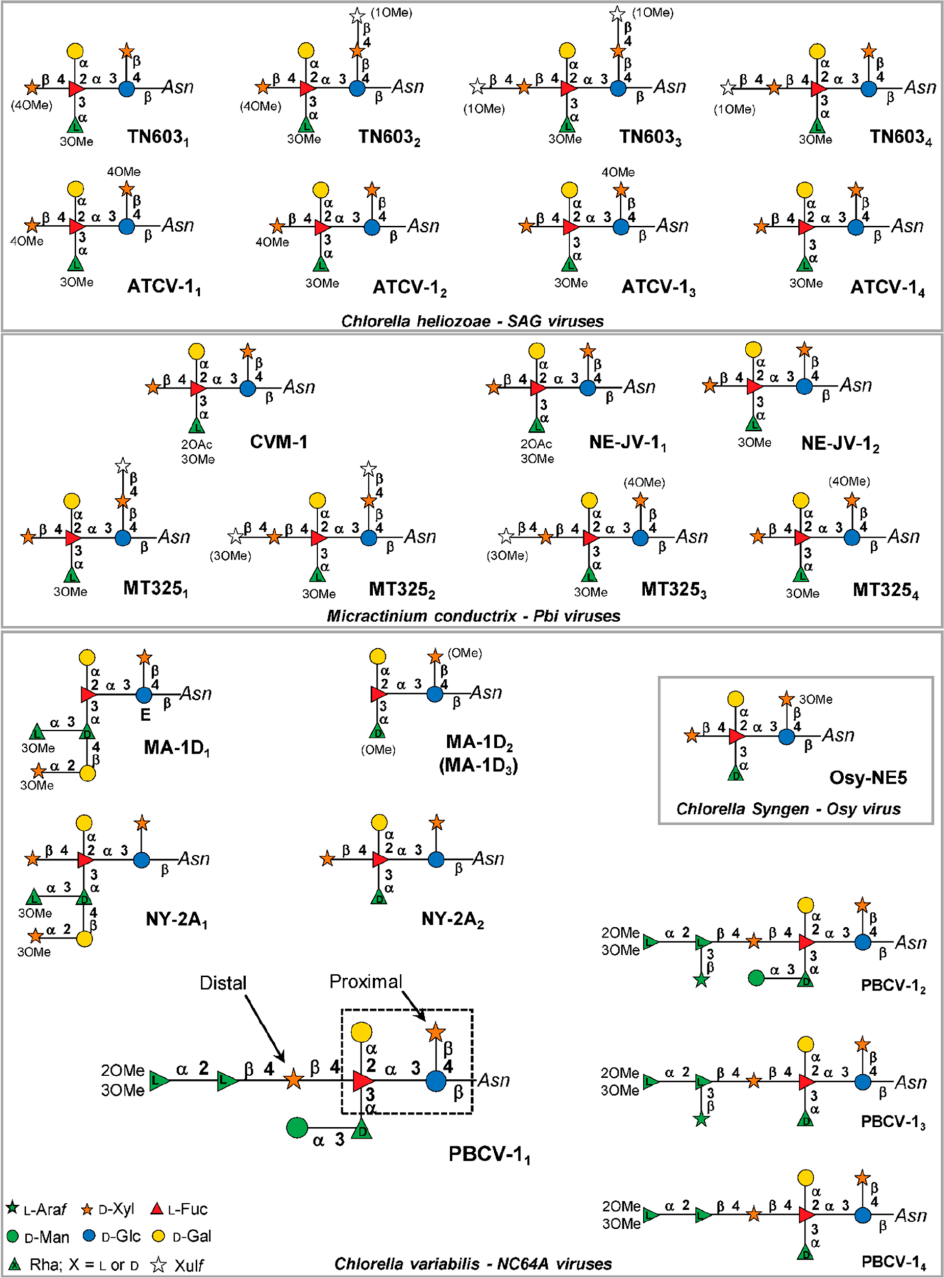
Structure of the *N*-glycans from all chloroviruses characterized to date. The structures from the same virus are arranged in a row, and the different viruses are grouped according to their host specificity, indicated at the bottom margin of the grouping boxes. Methyl groups enclosed in brackets denote a nonstoichiometric substitution. All of these *N*-glycans share a common core oligosaccharide that is indicated as a dashed box in the structure of PBCV-1. All sugars are in the pyranose form except where specified.

Determination of the structure of the PBCV-1 *N*-glycans^177^ occurred 20 years after their initial identification,^175^ when approaches commonly used for bacterial glycans were adapted to PBCV-1.^178^ Purified PBCV-1 particles were disrupted by heating to 60 °C, and the capsid protein recovered from the soluble fraction by precipitation with ice-cold acetone, which produced ∼90% purity. The chemical characterization of the monosaccharide constituents was either performed directly on the glycoprotein or on the glycopeptide blend released by enzymatic digestion with a protease. Notably, the *N*-glycan has Rha units with opposite configurations (Figure 9), and the evaluation of which residue was in a certain position was possible by incorporating the information on the linkage pattern into the method used to determine their absolute configuration.^177^

NMR spectroscopic studies were only conducted on the glycopeptides, because when the glycans were linked to the MCP, the NMR signals of the glycans were broad due to the low mobility of the macromolecule, which in solution spontaneously associates with two other copies to form trimers, termed capsomers.^176^ Accordingly, for NMR studies, Vp54 was digested with proteinase K, a highly efficient but unspecific protease that releases short glycopeptide fragments, sometimes consisting of only one (or two) amino acid(s). To determine the site of *N*-glycosylation, the enzymatic treatment has to release glycopeptides of sufficient length to identify their location in the sequence of the protein. Thus, for PBCV-1, thermolysin, which provided longer protein fragments, was used,^177^ while for other chloroviruses, the glycopeptide mixture produced by proteinase K was sufficient to identify most, if not all, of the glycosylation sites.^179^ Regardless of the protease used, the digestion products were a mixture of *N*-glycopeptides and peptides, and the glycopeptide components were purified via size exclusion chromatography (generally Bio-Gel P10). The fraction enriched in glycopeptides was suitable for both NMR spectroscopic and mass spectrometric analyses.^177,180^

This protocol led to the identification of four *N*-linked glycoforms of PBCV-1 (denoted as **PBCV-1**_**1–4**_, Figure 9), which have several remarkable features. First, the attachment of the glycans to the protein occurs via a β-Glc-Asn linkage, which is rare although it has been found in the three forms of life: the surface glycopeptides and flagellins of the halophilic archaea *Halobacterium halobium*(181) and *Halobacterium volcanii*,^182^ in the glycoproteins from *Haemophilus influenzae*(183,184) and *Actinobacillus pleuropneumoniae*,^185^ and in the B2 chain of rat kidney laminin, the only known example of this type of linkage in eukaryotes.^186^ Another feature that makes this type of *N*-glycosylation unique is the location of the Asn units linked to PBCV-1 glycans: three of the four sites share the sequence NTXT (the sequons are ^302^NTGT, ^399^NTET, and ^406^NTAT), whereas the fourth is ^280^NIPG. None are in the typical eukaryotic sequon NX(T/S), and collectively they denote a new pattern of *N*-glycosylation with implications and open questions about their biosynthesis.

Regarding the PBCV-1 Vp54 glycan, the main glycoform is a nonasaccharide (**PBCV-1**_**1**_, Figure 9) with a Fuc unit fully substituted at all available positions, not terminal as typically occurs in most *N*-linked glycans.^187^ Notably, a hyperbranched Fuc has only been reported in a complex phosphoglycan from *Trypanosoma cruzi*,^188^ but with a substitution pattern different from that found in PBCV-1. The Vp54 *N*-glycan contains both d- and l-Rha residues, a feature that is rare and so far only found in bacteria, along with a terminal l-Rha unit at the nonreducing end of the main chain capped with two methyl groups. This is an uncommon type of termination and so far only reported for glycolipids produced from *Mycobacterium hemophilum*(189) and *Mycobacterium leprae*.^190^

The four glycoforms of Vp54 glycans occur because Man and Ara*f* are not stoichiometric substituents. The two glycoforms, **PBCV-1**_**3**_ and **PBCV-1**_**4**_ (Figure 9), both devoid of Man and differing by the presence or absence of the Ara*f* unit, were present in traces, and their existence was only inferred by MALDI spectrometry studies. **PBCV-1**_**1**_ and **PBCV-1**_**2**_ were the main constituents of the glycopeptide mixture, and they were identified by NMR spectroscopy. MALDI mass spectrometry confirmed the NMR data and added further details. Apart from detecting the two minor glycoforms mentioned above, MALDI found that the *N*-glycosylation pattern was not the same at the different glycosylation sites. Indeed, ^280^Asn was glycosylated almost exclusively by **PBCV-1**_**2**_, while the glycosylation pattern at the other three Asn units was more heterogeneous, with **PBCV-1**_**1**_ as the main glycan, along with variable proportions of the other three.^177^

The purification protocol used for PBCV-1 enabled the identification of the glycan structures of other chloroviruses (Figure 9), classified either in the same group of viruses as PBCV-1 or in one of the other three chlorovirus groups (section 2.6.1).^179,191−193^ Of note, the chlorovirus NY-2A was predicted to have two MCPs with a high level of identity between them,^177^ and when this method was applied to NY-2A, both glycoproteins were extracted and the *N*-glycans derived from both proteins were studied together. Collectively, these structural studies established that the chlorovirus MCPs are always *N*-glycosylated and also revealed other interesting features. First, all chloroviruses glycans include the same oligosaccharide motif and, for this reason, termed conserved core oligosaccharide, marked in **PBCV-1**_**1**_ with a dashed box (Figure 9). Initially, this conserved oligosaccharide core consisted of five monosaccharide units:^179^ the four highlighted in Figure 9 together with a so-called distal Xyl residue. However, this view has recently changed after determining the glycan structure of chlorovirus MA-1D,^193^ a NC64A virus that produces three different *N*-glycans, all missing the distal Xyl unit. Accordingly, the conserved core region for all chloroviruses now consists of four sugar units (Figure 9).^193^

All chlorovirus *N*-glycans have a Rha unit linked at the third position of the hyperbranched Fuc, which is a semiconserved element because its absolute configuration is d for all the viruses within the NC64A and Osy groups, while it is l and always methylated at the *O*-3 position in the Pbi and SAG groups.^179^ The substitution pattern of the conserved core region inclusive of the semiconserved Rha unit appears to be virus-specific, and it can be considered as a glycan-based molecular signature for each virus. Indeed, several neutral monosaccharides can be part of this variable region of the *N*-glycan, such as xylulofuranose (Xul*f*), Ara*f*, or Rha and Xyl units substituted with various methyl or acetyl groups (Figure 9). Finally, these *N*-glycans are always branched, and their size varies from five, as **MA-1D**_**2**_, to 10 monosaccharide units, like the **PBCV-1**_**2**_ (Figure 9).

Regarding the position of the glycosylated Asn, MALDI MS investigation identified the *N*-glycosylation sites for some of them (NY-2A, CVM-1, and MA-1D).^179,193^ For all these viruses, the location of the glycosylated asparagine residue was in an atypical sequon when compared to that from all forms of life.

In chlorovirus NY-2A, the capsomer consists of two similar MCPs, B585L and B617L, both sharing a high level of homology with PBCV-1 Vp54. During the study of the two NY-2A *N*-glycans, the two MCPs could not be separated; therefore, five of the six glycosylation sites found (^54^NKVS, ^280^NIPG, ^302^NTGT, ^399^NTET, ^406^NTAT, and ^291^NVAT), could not be assigned exclusively to one of the two MCPs, while the sequon ^291^NVAT was assigned to B617L because it was not conserved in B585L.

With regard to MA-1D, another chlorovirus in the NC64A group, like NY-2A and PBCV-1, in silico analysis suggested the presence of two MCPs (named 609L and 635L),^193^ again with a very high level of homology between them and with the MCPs of both PBCV-1 and NY-2A. The two MA-1D MCPs were predicted to have the same, in terms of sequence and position, glycosylation sites as the other two viruses, and MALDI MS analysis confirmed three of them: ^280^NIPG, ^302^NTGT, and ^406^NTAT. The fourth position predicted to be glycosylated, ^399^NTET, was not detected; therefore, its glycosylation status is unknown.

Four glycosylation sites were detected in chlorovirus CVM-1, ^47^NGSV, ^279^NLTA, ^285^NVGY, and ^293^NTAV, making this virus the only one with a *N*-glycan attached to the canonical NX(T/S) consensus sequon. Of note: ^47^Asn is conserved in PBCV-1 and other MCPs, but it was only glycosylated in CVM-1.^179^

PBCV-1 is the only chlorovirus for which the structure of the capsid, along with the structure of the glycans has been determined.^36,108,176^ The PBCV-1 capsid has been thoroughly characterized by crystallographic and cryo-EM studies to 3.5 Å resolution, which revealed that it is composed of a set of 15 different proteins, with Vp54 as the most abundant and estimated to be present in ∼5040 copies per virion. Accordingly, the capsid includes 60 copies of the penton protein which is also a glycoprotein, presumably glycosylated by the same *N*-glycans as Vp54, while the collective number of the remaining 13 minor capsid proteins reaches ∼1800 copies.^36^ One of the 12 vertices of the capsid has a spike structure attached to it, and the proteins involved in the spike structure are currently unknown.

**Figure 10:**
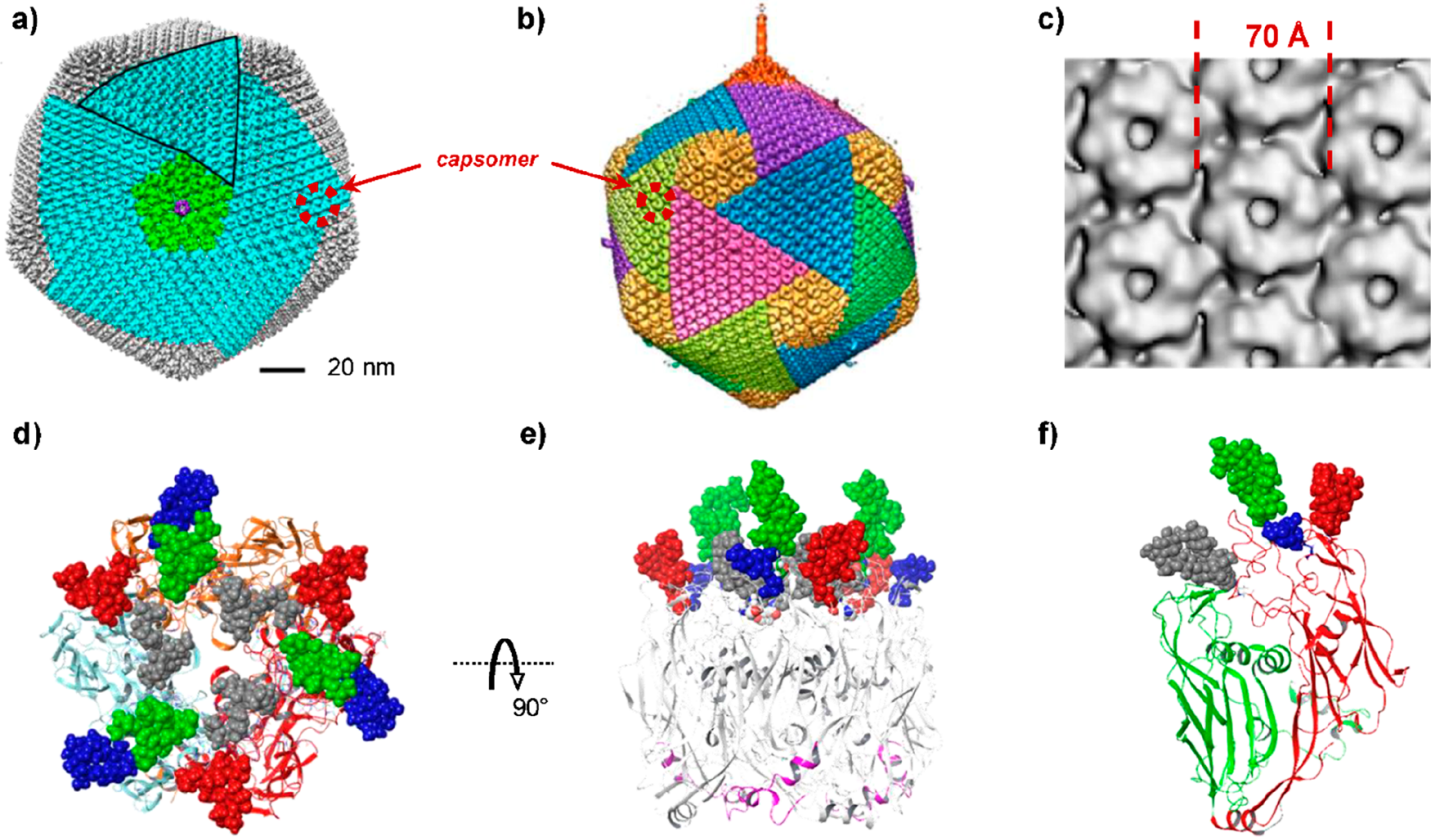
PBCV-1 capsid organization. (a) Top view of the cryo-EM reconstruction of the virus PBCV-1 capsid showing the trisymmetron (cyan, one explicitly contoured with a black line) and pentasymmetron (green) structures. Adapted with permission from ref (36). Copyright 2019 Springer Nature. Eleven of the 12 pentasymmetron have at their centers a pentamer made of the penton protein, drawn in violet. The twelfth pentasymmetron has a spike structure at the vertex (b). Both pentasymmetron and trisymmetron (a total of 20 in the capsid) structures are built by 66 and 30 capsomer units (c,d), respectively, with one highlighted by a red dotted circle, and expanded in (c–e). (b) different visualization of PBCV-1 capsid. Here each trisymmetrons is represented with a different color; pentasymmetrons are always in yellow, except that with the spike protein at its center, drawn in orange, and placed at the apical vertex of the capsid. Reproduced with permission from ref (101). Copyright 2020 Multidisciplinary Digital Publishing Institute. (c) Cryo-EM reconstruction of the capsomers. Reproduced with permission from ref (101). Copyright 2020 Multidisciplinary Digital Publishing Institute. (d) Top view of the capsomer structure from X-ray crystallography and molecular modeling: the complex is made by three units of the major capsid protein (Vp54). The peptide backbone of the three glycoproteins is presented in ribbon representation, each with a different color. The peptide part is partially hidden by the *N*-linked glycans, represented in the space filling-mode with their atoms colored according to the residue they are attached to (Asn-280, green; Asn-302, gray; Asn-399, red; Asn-406, blue). (e) Lateral view of the capsomer, with the first 24 amino acids of each MCP drawn in pink to highlight their location on the lower face of the complex. (f) Lateral view of Vp54 as a single monomer, with its two jelly roll domains, D1 (aa 2–212) and D2 (225–437) colored in green and red, respectively; the *N*-glycans follow the same notation as in (b), and this figure reports only those detected by X-ray crystallography. (d–f) Reproduced with permission from ref (108). Copyright 2018 Proceedings of the National Academy of Sciences of the United States of America.

This complex set of proteins is organized into two main motifs, the trisymmetrons and the pentasymmetrons (Figures 10a,b),^107,176^ present in 20 and 11 number of copies, respectively. Each of these is composed of many copies of the capsomer (Figure 10b–d): 66 for each trisymmetron and 30 for each pentasymmetron. Each of the 11 pentasymmetrons has a pentamer made of the penton protein in its center (Figure 10a). An additional pentasymmetron, the twelfth, differs from the others by having the spike structure in its center (Figure 10b). Each capsomer is composed of three copies of the MCP (Figures 10d,e), arranged with a pseudohexagonal symmetry resembling the shape of a doughnut (Figure 10c). In addition, one capsomer in each trisymmetron has a fiber that extends from the particle (Figure 10b, note the small violet fiber on the left side). The fiber is always located in a capsomer that is in the middle of the second row of capsomers.^106^

As for the Vp54 glycoprotein, the crystallographic experiments established that it consists of two consecutive domains held together by an α-helix, each of about 200 aa, with a jelly roll fold (D_1_, 2–212 aa; D_2_, 225–437 aa; Figure 10f). The first 24 amino acids are organized in two α-helices connected by a linker, and they are interconnected in the lower face of the capsomer (Figure 10e). In this structure, the N-terminal region of one Vp54 unit of the capsomer makes contacts with the nearby unit, thus stabilizing the trimeric complex. This interaction occurs on the side of the capsomer that is not glycosylated and that points toward the inside of the viral particle.

Domain D_2_ is glycosylated at positions ^280^Asn, ^302^Asn, ^399^Asn, and ^406^Asn, and the crystallographic data revealed only some of the sugar units (Figure 10f), namely those less flexible and located next to their respective glycosylation sites; the missing sugars were reconstructed by molecular modeling.^108^ This approach revealed that all glycans were located on the same side of the protein and that they pointed outward from the viral particle, with their outermost part free to move and able to assume multiple conformations.^108^ The role of the glycans in the interactions with the external environment or in the packaging process is an open question, and further experiments are needed to establish their importance. We deem that the oligosaccharide common core is essential for the stability of the particles, as no chlorovirus lacking glycans has been found to date.

#### 3.2.2 APMV Glycans Structures

The hallmark of the *Mimivirus* genus is the presence of a fibril layer around the icosahedral capsid (Figure 1e,j), including the tail in Tupanviruses (Figure 1b,c). Since the discovery of the first mimivirus APMV,^41^ the prototype of the clade A group (section 2.3.2.2, Table 1), it has been postulated that the fibrils are highly glycosylated due to the positive response of the viral particles to Gram staining. This first hypothesis has been experimentally validated for some of the viruses in three of the five clades by analyzing the monosaccharide composition of their fibrils (Table 3, section 3.1.2.5).^62,65,87^ APMV is the most studied virus of this genus, and recently the structures of its glycans have been resolved, as reported in the next two sections.^65,194^

##### 3.2.2.1 APMV Surface Proteins Are Decorated with *O*-Glycans

The occurrence of sugars associated with the fibrils was first confirmed in 2012,^62^ and this discovery prompted a study of the surface of the APMV particles, assuming that these sugars were linked to one or more proteins by *N*- and/or *O*-type linkages. In the first study by Hülsmeier and Hennet,^194^ the surface glycoproteins were extracted by applying a periodate oxidation method for the carbohydrates followed by biotinylation to isolate the glycosylated proteins and to establish their identity. This protocol led to the enrichment of 40 proteins, most of which were annotated as ORFans. These results were in agreement with those of others,^45,195^ except that other work predicted that the L425 protein was glycosylated,^196^ but it was poorly enriched by this procedure.

A sugar compositional analysis carried out on the pool of the extracted proteins revealed the presence of Rha, GlcNAc, Vio4NAc, and a methylated form of Vio4NAc, in agreement with previous data from Piacente et al.,^62^ along with traces of Xyl and Fuc. Additional structural information was obtained by performing a β-elimination reaction on the extracted proteins under reductive conditions, so that the *O*-glycans were converted to the corresponding reducing end alditols, which were isolated and investigated via chemical analysis and MALDI MS.

MALDI analysis of the methylated or of the perdeuteriomethylated oligosaccharide alditols detected 26 *O*-glycans, divided into six structural groups based on the nature of the first sugar attached to the protein. Using the compositional analysis data, the monosaccharides that composed these *O*-glycans were assumed to be Glc, GlcNAc, GlcN, Vio4N, and Vio4NAc, along with their methylated forms, but discussion of the MALDI structures will make use of the generic names (dHex, dHexN, Hex, HexN, and HexNAc) like in the original publication.^194^

**Figure 11:**
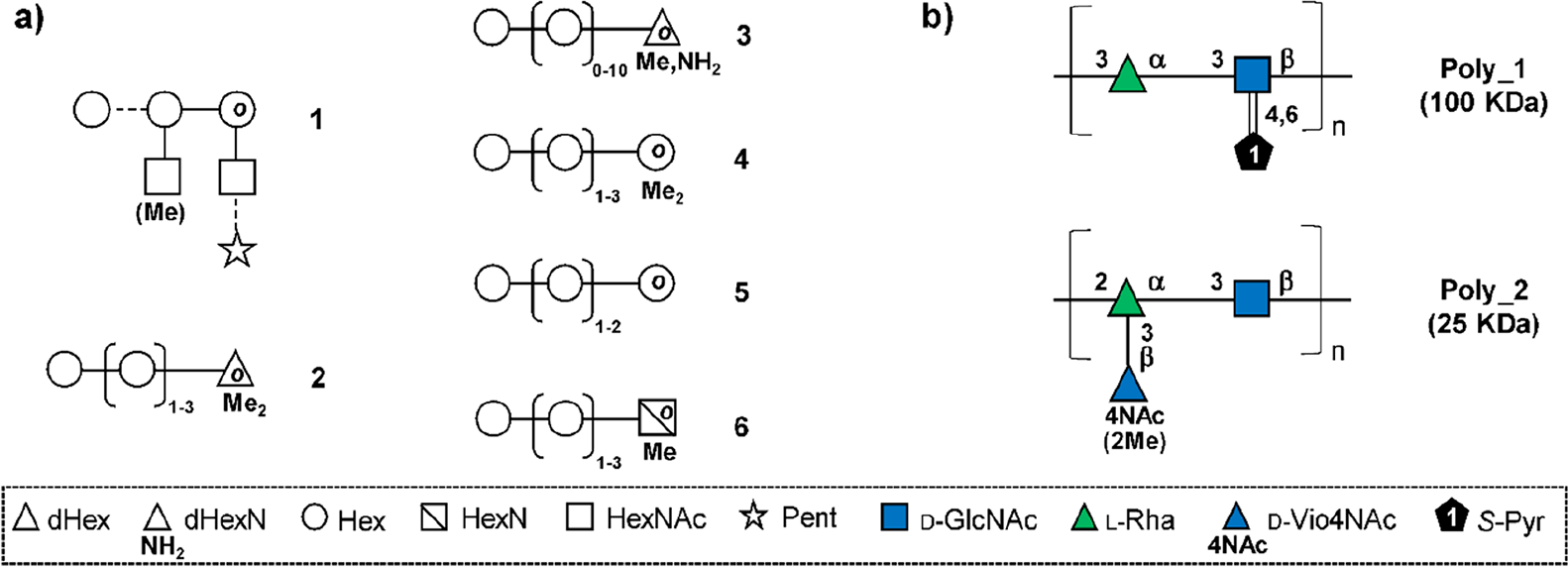
Glycan structures from APMV, the prototype of the clade A of the *Mimivirus*genus. (a) Structures of the APMV *O*-glycans. The numbers from 1 to 6 indicate the structural group to which the *O*-glycan belongs. The first group is the only one with a branched architecture, and it has a hexose (Hex) at the reducing end. The other groups are linear polymers of a hexose and differ in the nature of the monosaccharide at the reducing end: group 2 has a dimethylated dHex, group 3 has a methylated dHexN, group 4 has a dimethylated hexose, group 5 has a hexose, and group 6 has a HexN. On the basis of the carbohydrate chemical composition, the Hex is assigned to Glc, the dHex to Rha, dHexN to Vio4N, and HexN to GlcN. (b) The APMV fibrils contain two distinct polysaccharides, poly_1 and poly_2. The first has anionic features due to the presence of pyruvic acid linked as a ketal. The other, poly_2, is neutral, and the viosamine unit is always *N*-acetylated and partially methylated at position 2. All monosaccharides are represented according to the SNFG system and the “*o*” inside the symbol indicates that unit is an alditol. Nonstoichiometric substituents are indicated with the linkages as a broken line (a), or in parentheses (the methyl group in b).

The first group encompassed four branched *O*-glycans, made of 4–7 monosaccharide units, all sharing a methylated HexNAc at the nonreducing end, and a substituted Hex at the reducing end. Moreover, the two largest species had a terminal pentose (probably Xyl) linked to *O*-3 (or *O*-4) of the HexNAc unit attached to the Hex at the reducing end (Figure 11a). The glycans of all the other groups had a linear architecture, with a variable number of hexose units (Figure 11a).

Group 2 included three linear *O*-glycans all with a double methylated dHex at the reducing end. MALDI analysis of the perdeuteromethylated products suggested that the terminal Hex unit was attached to *O*-4 (or to *O*-6) of the tetrasaccharide (*n* = 2, Figure 11a) or at *O*-3 (or *O*-4) of the pentasaccharide (*n* = 3, Figure 11a).

The third group was the most abundant and encompassed 11 glycans of 2–12 monosaccharide units, all with a methylated dHexN at the reducing end. In this case, analysis of the MALDI spectra of the perdeuteromethylated alditols of the three oligosaccharides with *n* = 1 or 2 (Figure 11a) suggested a 4- or a 6-linkage between the Hex–Hex units, while the mode of attachment of the Hex to the dHexN could not be determined.

The fourth and fifth groups contained three and two glycans, respectively, and a double or a unmethylated hexose at the reducing end, respectively (Figure 11a). Regarding the nature of the glycosidic linkage between two consecutive Hex units, MALDI analysis of the perdeuteromethylated oligosaccharide alditols of group 4, suggested that the terminal nonreducing Hex was attached to *O*-3 or *O*-4 of the penultimate Hex, while the other glycosidic connections could also involve *O*-6. As for group 5, the same spectrometric approach suggested that the Hex–Hex glycosidic linkages involved either *O*-4 or *O*-6 of the next unit. Finally, the sixth group included three *O*-glycans with a methylated HexN at the reducing end. In addition, the MALDI-TOF-MS analysis suggested that the glycans of this group had 4-linked consecutive Hex units.

The authors also performed linkage analysis on the whole oligosaccharide mixture. They found 4-linked and terminal non substituted Glc units as major components, which supported the MALDI results and led to the hypothesis that some *O*-glycans had an amylose-like arrangement of the Glc units. To prove this hypothesis, the APMV particles were treated with α-amylase and their infectivity compared to that of the untreated virions, but no difference was observed between the two groups, suggesting that these glycans were not essential for viral infection.

Regarding their biosynthetic mechanisms, at the time of this study information about the GTs encoded by APMV were limited to the discovery of a bifunctional enzyme, L230, that hydroxylates the lysine residues of a collagen-like viral protein and transfers a Glc unit to it (see section 3.3.3).^24^ Therefore, the authors speculated that APMV highjacked the host enzymes to add the pentose unit onto the HexNAc unit and/or to methylate some of the monosaccharide units based on the information that some *Acanthamoeba* species have *N*-glycans with terminal sugars methylated or pentosylated. There was no information provided about the GTs involved in the assembly of the other glycosidic linkages.^197^ This study was extremely important because it was the first to provide clear insight into how complicated the structure of the APMV fibrils are.

##### 3.2.2.2 APMV Fibrils Are Covered with a Glycocalyx Consisting of Two Polysaccharides

Notaro et al.^65^ recently analyzed APMV glycan fibrils using an experimental approach different from that of Hülsmeier and Hennet.^194^ The fibrils were released from the virions by heat treatment under denaturing conditions realized with dithiothreitol. The NMR spectroscopic data collected on the crude product revealed the presence of two distinct polysaccharides (Figure 11b). Polysaccharide 1 (poly_1) was an acidic polysaccharide with a linear repeating unit made of 3)-α-l-Rha-(1→3)-β-d-GlcNAc-(1→, with GlcNAc having a pyruvic acid linked at positions 4,6 as a ketal with the *S* configuration at its stereocenter. Poly_2 instead (Figure 11b) was a neutral polysaccharide with a branched repeating unit: the linear backbone consisted of a disaccharide repeating unit with the sequence (2)-α-l-Rha-(1→3)-β-d-GlcNAc-(1→, with Rha further substituted at *O*-3 with β-d-2OMe-Vio4NAc (80%) or with β-d-Vio4NAc (20%). From a structural point of view, these two polysaccharides are unique but have some resemblance to those of bacterial origin. In a particular, poly_1 resembles those produced by some strains of *Klebsiella pneumoniae*,^198^*Serratia marcescen*s,^198^ and *Agrobacterium tumefaciens*,^199^ albeit these all lack the pyruvic acid moiety.

The two polysaccharides were purified by anion exchange chromatography, and their molecular weights (MW) were 100 and 25 kDa for poly_1 and poly_2, respectively. Importantly, the enrichment of the two polysaccharides in pure form was possible only by digesting the fibrils with proteinase K, indicating that they were attached to the same polypeptide backbone. In agreement with this hypothesis, the MW of the intact fibrils was about 400 kDa, a value much higher than that of the single polysaccharides. After protease treatment, the mass of the recovered material dropped considerably, leading to the conclusion that the two polysaccharides constituted about 13% (w/w) of the intact fibrils, also allowing their respective MW determination.

The authors collected preliminary information about the identity of the protein/s acting as carriers, albeit the nature of the glycosylation sites could not be determined due to the difficulties in applying consolidated proteomics methods to glycoproteins with high MW glycans. However, this approach defined the proteome of the fibrils, and this ensemble of proteins was refined through a bioinformatic screening that identified L894/L893 as the most promising candidate to carry one or more copies of the two polysaccharides. This protein addressed different requirements: it was highly conserved among all the members of the A–C clades of the *Mimivirus* genus, its PI expression time matched that of other proteins known to be involved in the sugar-nucleotide metabolism, and it had several serine residues predicted to be glycosylated.

The finding that polysaccharides form a glycocalyx around APMV has changed the view that viruses of any type (giant or not) are only covered with oligosaccharides of discrete size. This discovery officially marks the entry of polysaccharides into the viral world, illustrating that these macromolecules are not limited to the cellular world. Moreover, these glycans are attached to one or more carrier proteins, thus placing the phenotype of APMV glycocalyx at the interface between the microbial and eukaryotic world.

On the basis of the information available for the M4 isolate, a mutant of APMV devoid of the external fibrils,^195^ it appears that APMV mimics bacteria with its polysaccharide coating. The authors hypothesize that this fibril architecture provides an advantage both during phagocytosis and in the competition with bacteria and other viruses on which the amoebae feed. This hypothesis is supported by the finding that uptake of M4 by the host is less efficient compared to that of the wild-type virus, even though the lack of fibrils does not impair the ability of the virus to replicate once it infects the host.^200^ To demonstrate the role of the glycans of the fibrils in the infection process, the authors performed competition experiments by using the wild-type virus, APMV, together with some monosaccharides. The authors found that GlcNAc and Man reduced the ability of APMV to adhere to the amoeba and observed a decreased infection rate. Finally, the authors found that APMV exploits the glycans of its fibrils to adhere to other organisms, like fungi and insects, without infecting them. This finding prompted us to hypothesize that these organisms act as dispersers and contribute to the spread of the virus in the environment.^200^

These studies with APMV have clearly expanded the scope of viral glycobiology studies and have demonstrated that there is still much to learn about giant viruses and their glycans. By looking at the fibril architecture, it appears that the viruses possess a complex glycosylation pattern that consists of oligosaccharides of discrete length (Figure 11a) and polysaccharides (Figure 11b). The way these glycans are linked to their protein carriers is an unanswered question, as are other challenging questions such as: How do these glycans contribute to the fitness of the virus in the environment? How do they promote the ability of the virus to infect the host? How do they shield the virus against virophages.

### 3.3 Glycosyltransferases

As mentioned in section 2, giant viruses encode many glyco-related genes, with many identified as GTs. How these viruses use the GTs has long been an intriguing question, and currently it is possible to give an answer for only some representatives of the chloroviruses and mimiviruses.

The first experimental results indicated that some chloroviruses remodel the cell wall of the host during infection (section 3.3.1). Then, determination of the glycan structures attached to the MCP of the chlorovirus PBCV-1 advanced the research on its GTs (section 3.2.2). Other information has been obtained about two GTs encoded by mimivirusAPMV (section 3.3.3). The next subsections will discuss what is known about the viral-encoded GTs and also mention some open questions.

#### 3.3.1 Chloroviruses Remodel the Host Cell Surface

**Figure 12:**
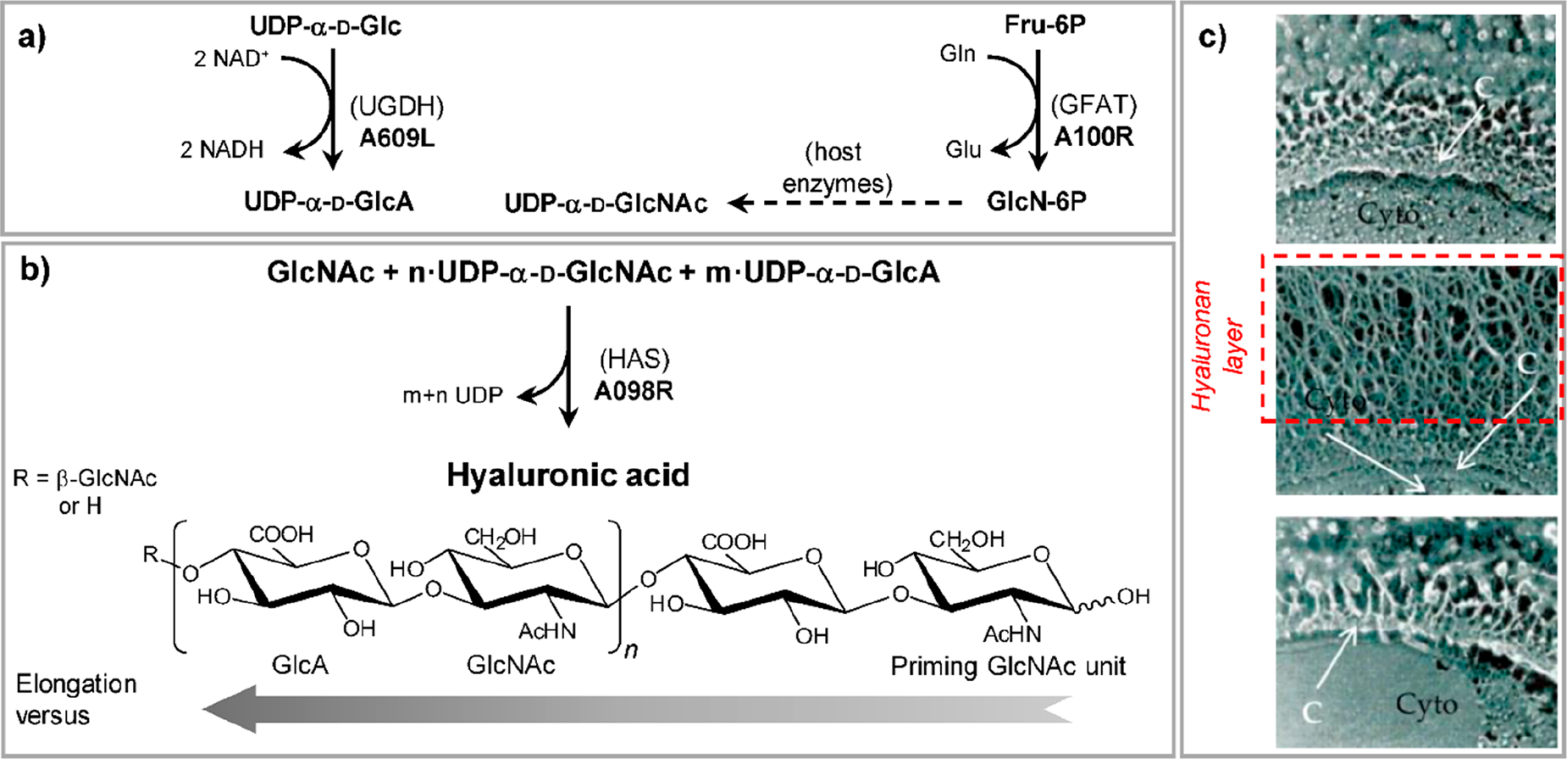
PBCV-1 hyaluronic acid biosynthesis. (a) Activities of the viral enzymes A609L and A100R, involved in the production of the two nucleotide sugars, UDP-α-d-GlcA and UDP-α-d-GlcNAc, respectively. (b) Synthesis of hyaluronic acid catalyzed by A098R; notably, this enzyme requires GlcNAc as primer to add the other monosaccharide units, thus elongating the polysaccharide chain from the nonreducing end. (c) Electron microscopy images of the *Chlorella* cell wall: top, uninfected algae; middle, image of the cell wall taken 240 min PI; bottom, cell wall of the infected algae treated with hyaluronidase. “Cyto” indicates the cytoplasm of the cell, while “C” the cell wall. (c) Adapted with permission from ref (205). Copyright 1999 Elsevier.

The first connection between giant viruses and glycobiology occurred in the late 1990s, when it was discovered that PBCV-1 encoded a functional hyaluronan synthase (HAS, gene *a098r*).^11^ At that time, HAS had already raised the interest of enzymologists due to the fact that it had broken the dogma that one enzyme could have only one GT function. On the contrary, HAS had two GT specificities, one for each of the two sugars, glucuronic acid and *N*-acetyl glucosamine, that are required for hyaluronan synthesis. PBCV-1 added to the story in that a virus encoded such an enzyme.^11^ Moreover, the activity of the PBCV-1 A098R protein was supported by two other virally encoded enzymes necessary to produce the sugars composing hyaluronan (Figure 12a): UDP-Glc dehydrogenase (UGDH, 389 aa, gene *a609l*) synthesizes UDP-α-d-GlcA, and glutamine:Fru-6P aminotransferase (GFAT, 595 aa, gene *a100r*) is involved in GlcNAc synthesis (Figure 12a, Table 2). The closest homologues of A100R and A609L enzymes are from bacteria, *Aquifex aeolicus* (41% identity over 602 aa) and *Vibrio cholerae* (56% identity over 390 aa).^146^

HAS enzymes are divided into two classes, I and II, with class II having HAS from the bacterium *Pasteurella multocida* as a unique member.^201^ PBCV-1 HAS belongs to class I based on its amino acid sequence and structure, it is closer to eukaryotic enzymes (45% sequence similarity with the human HAS2)^145^ than to the bacterial counterparts,^146^ and its structure was recently determined by a combination of cryo-EM, molecular modeling, and functional studies.^145^

The PBCV-1 HAS enzyme was unexpected because, until then, hyaluronan had only been found as a component of the extracellular matrix of vertebrates and of the extracellular capsule of some pathogenic bacteria.^202^ Hyaluronan is a linear polysaccharide belonging to the family of glycosaminoglycans^203^ composed of alternating 4-linked β-d-GlcA and 3-linked β-d-GlcNAc residues, with MWs up 10^6^ Da (Figure 12b). As a fundamental component of the extracellular matrix in vertebrates, it interacts with several proteins such as CD44, RHAMM, and fibrinogen. Thus, it is involved in several cellular processes such as angiogenesis, cancer, cell motility, wound healing, and cell adhesion.^204^

PBCV-1 HAS is 568 aa long with high similarity to both vertebrate and bacterial HASs.^11,205^ The gene was expressed in *E. coli*, and biochemical assays demonstrated that the recombinant protein requires manganese for activity,^11,145^ opposite to the streptococcal and mammalian HASs that prefer magnesium.^201^ The structure consists of six transmembrane helix and three interface helix domains which enclose a cytoplasmatic region endowed with a GT-A fold and that possesses the catalytic activities necessary to produce hyaluronan. To this end, A098R requires GlcNAc monosaccharide as a primer for the polymerization reaction, which occurs by the sequential addition of GlcA and GlcNAc, by using the corresponding UDP nucleotide sugars as activated donors of the reaction. Thus, the polysaccharide chain elongation occurs on the nonreducing end of the polymer, like HAS class II enzymes, and opposite to what was reported in an earlier study (Figure 12b).^204^ Interestingly, the high sequence similarity (about 45%) between the viral HAS and the human HAS2,^145^ one of three human encoded enzymes and the only one so far proved as essential, suggests that the same mechanism applies to HAS2, if not to many other class I enzymes.

Expression of the three viral genes, *a098r*, *a100r*, and *a609l*, has major consequences on the morphology of the host *Chlorella* (Figure 12c). The three genes are expressed early in viral infection, and hyaluronan fibers appear on the infected cell surface within 30 min PI. The fibers continue to accumulate on the chlorella cell wall surface for at least 4 h and cover the entire surface of the infected algae, as revealed by quick-freeze, deep etch microscopy analysis (Figure 12c). The fibers can be removed by treatment with hyaluronidase, restoring the phenotype of the cell wall back to that of the uninfected algae (Figure 12c).^205^

Because PBCV-1 produces three enzymes involved in hyaluronan biosynthesis, this polysaccharide would seem to have an important function in the PBCV-1 life cycle. However, this hypothesis was soon challenged as the *a098r* gene is not present in all chloroviruses that infect *Chlorella* NC64A,^205^ indicating that this enzyme is not always essential for replication. One explanation is that the *has*-lacking viruses might encode an enzyme(s) that produces an alternative polysaccharide on the external surface of the infected *Chlorella* cells.

This hypothesis was confirmed by Kawasaki et al.,^206^ who reported that some chloroviruses have a gene encoding a functional chitin synthase (CHS) instead of HAS and that these viruses produced chitin fibers surrounding the external surface of infected *Chlorella* cells. Furthermore, some chloroviruses (e.g., CVIK1 and CVHA1) contained both *has* and *chs* genes. These viruses therefore produce both hyaluronan and chitin polysaccharides simultaneously on the surface of the infected host cells,^206^ suggesting that there is no functional incompatibility between the two genes or their products. Even more interesting is the fact that some chloroviruses lack both *has* and *chs* genes and do not produce any extracellular polysaccharide on the surface of their host after infection.

Like the *has* gene, the *chs* gene is expressed as early as 10 min PI, producing chitin, which is rare in algal cell walls, although it has been reported in some green algae.^207^ Further support for the presence of chitin on the external surface of the infected chlorella cells was provided by the observation that treating the algae with chitinase abolished the dense fibrous network.^206^ The CHS enzyme belongs to the GT2 family of processive polymerizing GTs, which produces chitin, a homopolymer of 4-linked β-d-GlcNAc, that may act both as a structural element and as a signaling molecule, similar to the hyaluronan.

Comparative genomic analysis between viruses CVK2 and PBCV-1 revealed high identity except for a few differences. Among them, the region corresponding to the PBCV-1 *has* gene was absent in CVK2 and replaced by a 5 kb region containing fragmented ORFs, some of which had similarity to *chs* genes from other species, together with *udp-gdh2* (a gene encoding a second UGDH).^206^ Comparison of the predicted amino acid sequence of CVK2 *chs* with those from various organisms indicated that it had a high similarity to the CHS3-type enzyme from yeast and fungi, even though its size was much smaller than the fungal enzymes (516 amino acids compared to ∼1200 amino acids) with the sequence homology restricted to the C-terminal region, where the catalytic site occurs. The smaller size of the CVK2 CHS protein could be explained by its simpler regulatory and processing mechanism or by a different location in the cell.^208^ Kawasaki et al. also demonstrated that the newly synthesized chitin was efficiently secreted across the *Chlorella* membrane and cell wall to the outside of the infected cell, meaning that the CVK2 CHS is probably located in the cell plasma membrane, where the chitin molecules are synthesized and pushed through the cell wall.^206^ The *chs* gene has also been found in NY-2A and AR158 chloroviruses as well as in other large dsDNA viruses, such as Ectocarpus siliculosus virus EsV-1 (*Phaeovirus* genus of the *Phycodnaviridae* family, Table 1),^114^ and Fadolivirus (*Klosneuvirinae* subfamily, in *Mimiviridae* family, Table 1).

Interestingly, all chloroviruses contain the *gfat* gene required for the synthesis of GlcNAc, a sugar common to both hyaluronan and chitin polysaccharides.^13^ In contrast, some chitin-synthesizing viruses loose *ugdh* used for the synthesis of UDP-α-d-GlcA, necessary for hyaluronan synthesis.^13^ Therefore, it could be hypothesized that for viruses that produce chitin, the components of HA synthesis become obsolete and are lost.^208^

The synthesis of hyaluronan and/or chitin requires a huge amount of energy, and the destiny of the host cell at the end of the infection process is lysis to release the progeny viruses. Therefore, why the chloroviruses synthesize hyaluronan and/or chitin is unknown. Another interesting question is how do the hyaluronan and chitin fibers pass through the cell wall. In fact, when the chlorovirus *has* gene was expressed in a higher plant, hyaluronan did not pass through the cell wall but collected between the plasma membrane and the cell wall.^209^ The ability of chloroviruses to manipulate the host by modifying its cell surface with other polysaccharides is remarkable, although the reason for this manipulation and the advantages gained for the virus await to be discovered.

#### 3.3.2 Chloroviruses Encode the GTs that Synthesize Glycans Attached to Proteins

##### 3.3.2.1 Historical Background

It was predicted in 1993 that the chloroviruses might encode most, if not all, of the machinery to glycosylate their MCP(s) and that the process occurred in the cytoplasm.^175^ For the chlorovirus PBCV-1 MCP (Vp54, gene *a430l*), this conclusion originally arose from antibody studies.^175^ Rabbit polyclonal antiserum prepared against intact PBCV-1 particles inhibited virus plaque formation by agglutinating the virions. However, spontaneously derived, antiserum-resistant plaque forming PBCV-1 antigenic variants occurred at a frequency of 10^–5^ to 10^–6^. Polyclonal antiserum prepared against members of each of these antigenic classes reacted primarily with the Vp54 equiv from the viruses used for each immunization. The Vp54 proteins from the antigenic variants migrated faster on SDS-PAGE than that from WT. However, the nucleotide sequence of the *a430l* gene in each of the variants was identical to the WT gene, which verified that the polypeptide portion of Vp54 was not altered in the mutants. Western blot analyses of Vp54 proteins isolated from the variants, before and after removing the glycans with triflic acid or altering the glycan with periodic acid, also supported the notion that the antigenic variants reflected differences in the Vp54 glycans, not the Vp54 polypeptide.^175^ Notably, the antigenic variants were grown in the same host so that the glycan differences were not due to the host but to the genes encoded by the virus, thus supporting the idea that the virus has its own glycosylation machinery. These results indicated that the differences between the different capsid proteins resided in the size of the glycans.

Subsequent DNA sequencing and annotation revealed that the PBCV-1 genome encoded eight putative GTs genes (*a064r*, *a071r*, *a075l*, *a111/114r*, *a219/222/226r*, *a301r*, *a473l*, *a546l*), some of which were predicted to be involved in MCP glycosylation, in addition to the hyaluronate synthase gene *a098r* (section 3.3.1).^13,101^ The cellular protein localization program PSORT predicted that most of these GTs were soluble and located in the cytoplasm, i.e., they lacked an identifiable signal peptide that would target them to the ER and Golgi.^13^ The exceptions were A473L and A219/222/226R, which were predicted to contain six and nine transmembrane domains, respectively.^13^ Moreover, these eight genes are expressed early during PBCV-1 infection,^210^ suggesting they were good candidates for adding sugars to the Vp54 MCP during viral replication.

**Figure 13:**
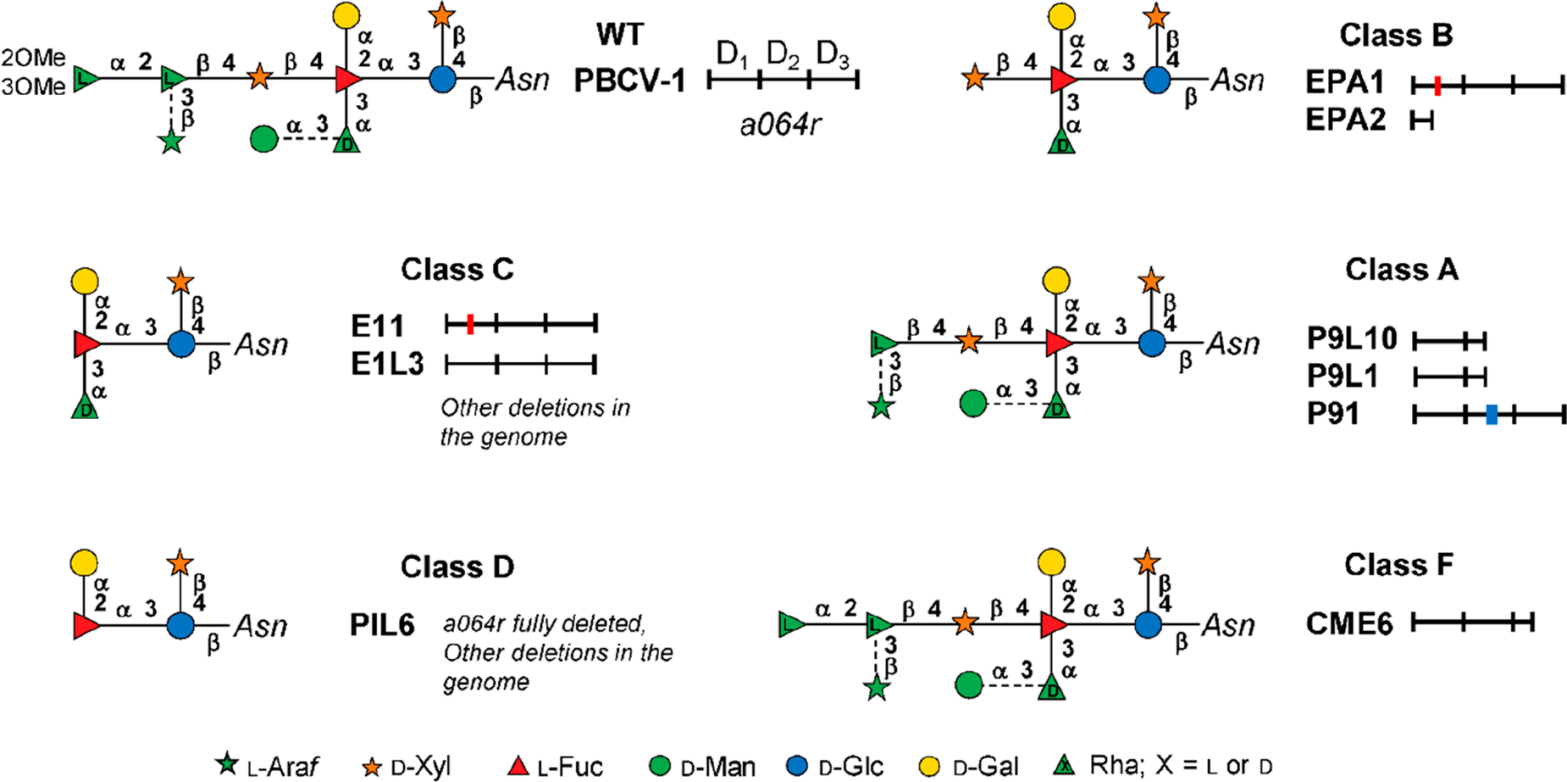
The *N*-glycans of PBCV-1 antigenic variants. Most of the antigenic variants have a mutation in the *a064r* gene that encodes the protein A064R with three domains (D1, D2, and D3), as sketched near the structure of the *N*-glycan representative of each antigenic class, including the wild-type PBCV-1. A red mark indicates a point mutation, a blue mark indicates an insertion, and the length of the segment is proportional to the level of truncation of the translated protein. All sugars are in the pyranose form except where specified; linkages drawn with a broken line denote a nonstoichiometric substitution.

Many of the PBCV-1 antigenic variants had mutations in *a064r*, a gene encoding a protein with three catalytic domains, or had the gene deleted and/or deletions elsewhere in the genome (Figure 13). The variants are organized into six antigenic classes, A to F, based on their different reactions to polyclonal antiserum, and representatives from all the antigenic classes (except E) have been studied. The antigenic class B had a mutation in A064R domain 1; the class A domain 1 was intact but the second domain had mutations. Class C included viruses in which no mutations occurred in the *a064r* gene (except E11), but mutations occurred elsewhere in the genome (Figure 13). Viruses that had large deletions in the region nearby and inclusive of *a064r* were members of the D antigenic class. Finally, class F is characterized by an early truncation of the third domain of the A064R protein and includes only one virus.^13,143^ The structure of the *N*-glycans of some representatives of each antigenic class have been determined and related to the type of mutation in the virus, thus leading to the identification of the potential functions of some of the virus-encoded GTs.

##### 3.3.2.2 The Proteins A064R and A061L (Table 2)

**Figure 14:**
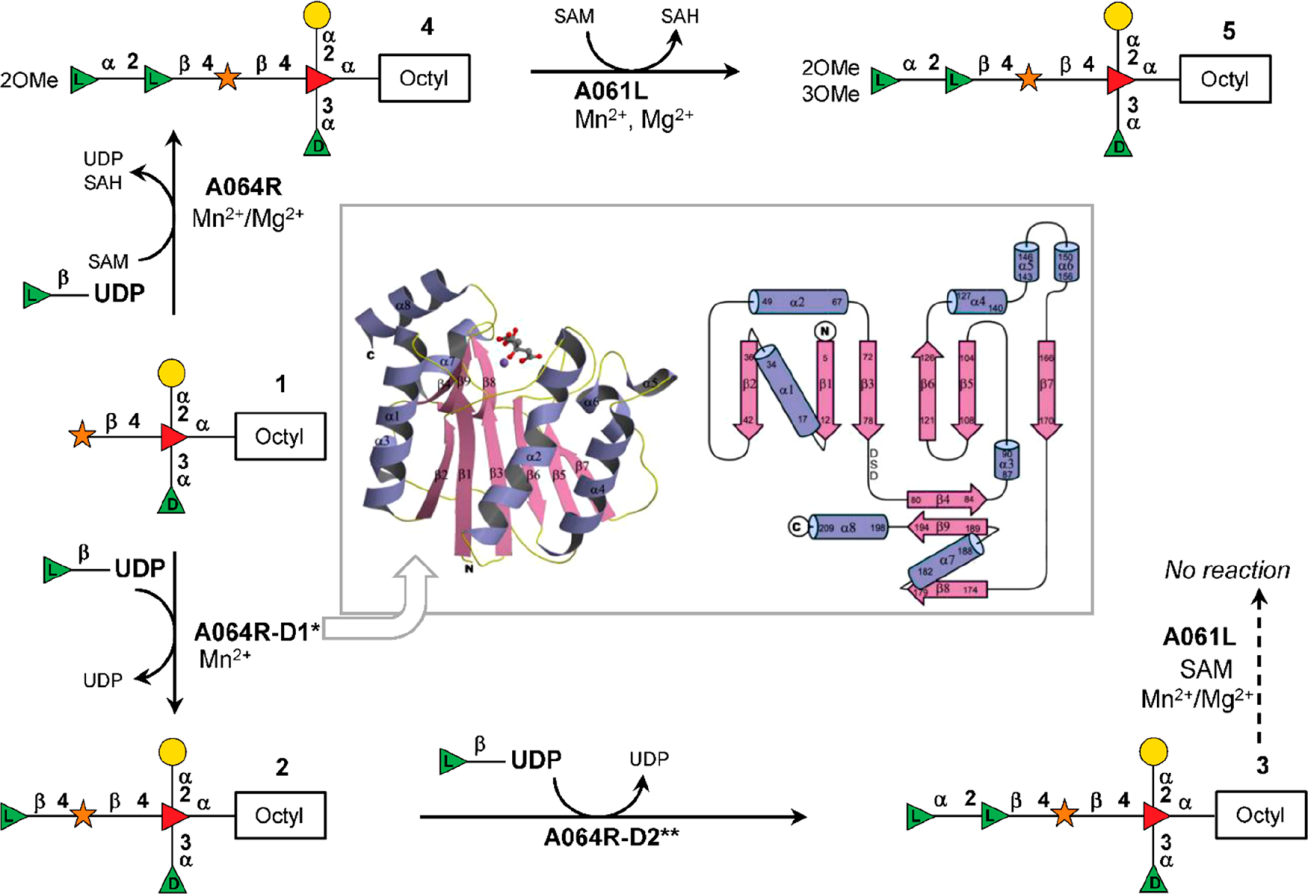
Biochemical assays of A064R and its individual domains. Substrate **1** was used as an analogue of the *N*-glycan from a class B variant (Figure 13), and it was transformed into **2** by A064R-D1 and, in turn, converted into **3** by A064R-D2. Substrate **1** was used to assess the activity of the complete A064R and validated domain 3 as responsible for adding a methyl group to the *O*-2 position of the terminal l-Rha. Product **4** (but not **3**) was the substrate for A061L, which added the methyl to *O*-3 of the terminal l-Rha. The inset at the center displays the crystallographic structure of A064R-D1 and the sequence of the secondary structural motifs. Adapted with permission from ref (142). Copyright 2007 Elsevier. *A064R-D1 could also use Xyl as a substrate. **A064R-D2 could also use Rha as a substrate.

The *a064r* gene encodes a 638 amino acid protein, and in silico analysis predicted that it had three domains of ∼200 amino acids each. The N-terminal domain (A064R-D1, from 1 to 212 aa) was the first to be studied due to its resemblance to a GT, and its crystallographic structure was determined before its GT specificity was known (inset in Figure 14).^142^ This domain has a mixed α/β fold containing a central, six-stranded β-sheet flanked by α-helices and a small, two-stranded β-sheet. The fold is similar to catalytic GT domains in the GT-A group, in subfamily 34, although the amino acid sequence similarity is very low. This GT subfamily has a retaining mechanism and the typical DXD motif that coordinates the sugar nucleotide phosphate together with a divalent cation.^142^ Furthermore, this study established the A064R-D1 specificity for UDP- rather than other sugar nucleotides, recognizing UDP-α-d-Glc as the preferred sugar donor and Mn^2+^ as the bivalent cation.^142^ It took several years to determine that not all of these initial assumptions were correct. Domain 2 (A064R-D2, 213–405 amino acids) resembled a few proteins of unknown function in GenBank, and the C-terminal (A064R-D3, 439–638 aa) was predicted to be a methyltransferase. The activities of each of the A064R domains became clear once the *N*-glycan structures of the antigenic variants were determined (Figure 13).^143^

First, the *a064r* gene of the two representatives in the antigenic class B (EPA1 and EPA2) led to proteins either with a point mutation or a truncation in the first domain (A064R-D1), and the *N*-glycans of the two mutants were identical and truncated at the level of the distal Xyl, with β-l-Rha missing compared to the *N*-glycan of wild-type PBCV-1 (Figure 13).

In contrast, variants belonging to the antigenic class A (P91, P9L1, P9L10) had the first A064R domain intact, and their *N*-glycans were truncated at the level of the β-l-Rha unit, suggesting that the A064R-D1 function was related to the formation of the β-l-Rha-(1→4)-Xyl linkage.^143^ A064R from the CME6 mutant had a truncation in the third domain (Figure 13), and the corresponding *N*-glycan had the terminal α-l-Rha unit, but it was not methylated like wild-type PBCV-1.^143^ These findings indicated that the second domain of A064R was a α-l-Rha transferase, while the third domain was a methyltransferase, although it was not clear if the third domain had a single or a double transferase activity.

All of these hypotheses were validated by expressing both the entire A064R protein and/or each domain individually and testing their activities in vitro with appropriate substrates.^141^ All proteins were expressed in *E. coli* cells as fusion proteins and UDP-β-l-Rha was used as a donor substrate for both A064R-D1 and A064R-D2 (Figure 14). This sugar-nucleotide was prepared by using the APMV enzyme L780 (section 3.1.2.1). The experiments were conducted in the presence of either Mn^2+^ or Mg^2+^; EDTA was used as a chelating agent to investigate the cation dependency of the reaction, and the reaction products were analyzed via NMR spectroscopy (Figure 14).^141^

The synthetic oligosaccharide **1** (Figure 14) was used as a simplified version of the EPA1 *N*-glycan as substrate for A064R-D1. This experiment confirmed that the first domain (amino acids 1–212) was the β-l-rhamnosyltransferase that transferred the Rha unit from the donor onto the fourth position of Xyl in **1** to give product **2**. Notably, this enzyme could also use Xyl as the acceptor monosaccharide, while it could not transfer Glc, the donor hypothesized in the crystallographic study. Moreover, the reaction occurred via a retaining mechanism and in the presence of Mn^2+^ cation, in agreement with the previous work.^142^

A064R-D2 was assayed by using **2** as a substrate (Figure 14); the assay confirmed that D2 was the α-l-rhamnosyltransferase that links the α-l-Rha unit to *O*-2 of the β-l-Rha unit to give **3** via an inverting mechanism without the need for a cation. The cloning of this enzyme also revealed the limits of the in silico analyses. The size of the first construct (amino acids 191–405) matched that of the in silico analyses but was not active; however, the protein with an additional 35 amino acids at the C-terminus was. Of note, A064R-D2 was produced in two versions, D2L (amino acids 191–438) and D2L_2_ (amino acids 213–438); both were active and could also glycosylate free rhamnose in addition to **2**.

The discovery of A064R-D2 activity was significant because this domain has no sequence homology with annotated rhamnosyltransferases (<30% with only 15% coverage), making it an outlier in a phylogenetic analysis. BLASTp investigation revealed that the A064R-D2 domain shares partial homology with many hypothetical and uncharacterized bacterial proteins, including one GT-family protein from *Lacipirellula parvula* (31.4% identity with 95% coverage) and two homologues (a hypothetical protein and a GT10 fucosyltransferase) in *Pithovirus*. Hence, A064R-D2 represents a new GT family.

The activity of the A064R-D3 domain was validated on the entire A064R enzyme together with acceptor **1**, with two equivalents of UDP-β-l-Rha and SAM, in the presence of both Mn^2+^ and Mg^2+^ (Figure 14). The reaction produced product **4**, thus confirming the activities of the first two domains of A064R and demonstrating that the third domain added only one of the two identified methyl groups, that at the *O*-2 position of the terminal α-l-Rha unit. The activity of the third domain was confirmed by a pBLAST search, returning many class-I SAM-dependent methyltransferases from both bacteria and viruses as the best matches, as well as several bacterial hypothetical proteins. This finding also suggested that there was another enzyme involved in the methylation at the *O*-3 position.^141^

PBCV-1 genome screening identified the *a061l* gene as encoding a 209 amino acid protein that shared high sequence similarity with several well-characterized class I SAM-dependent methyltransferases from bacteria and algae.^141^ A061L was expressed as a recombinant protein with a GST-tag, its activity was assayed using SAM as the donor of the methyl group, both Mn^2+^ and Mg^2+^ as bivalent cations, and two different acceptors (Figure 14): one having the terminal α-l-Rha methylated at *O*-2 (**4**) and the other lacking the *O*-2 methylation (**3**). The experiment established that A061L was the methyltransferase that added the second methyl group to the terminal Rha. Moreover, this work showed that it had a specific substrate requirement, attaching the methyl group only if Rha was already methylated at *O*-2. Indeed, **3** was not transformed into a different product, while **4** gave the final product **5** (Figure 14).

**Figure 15:**
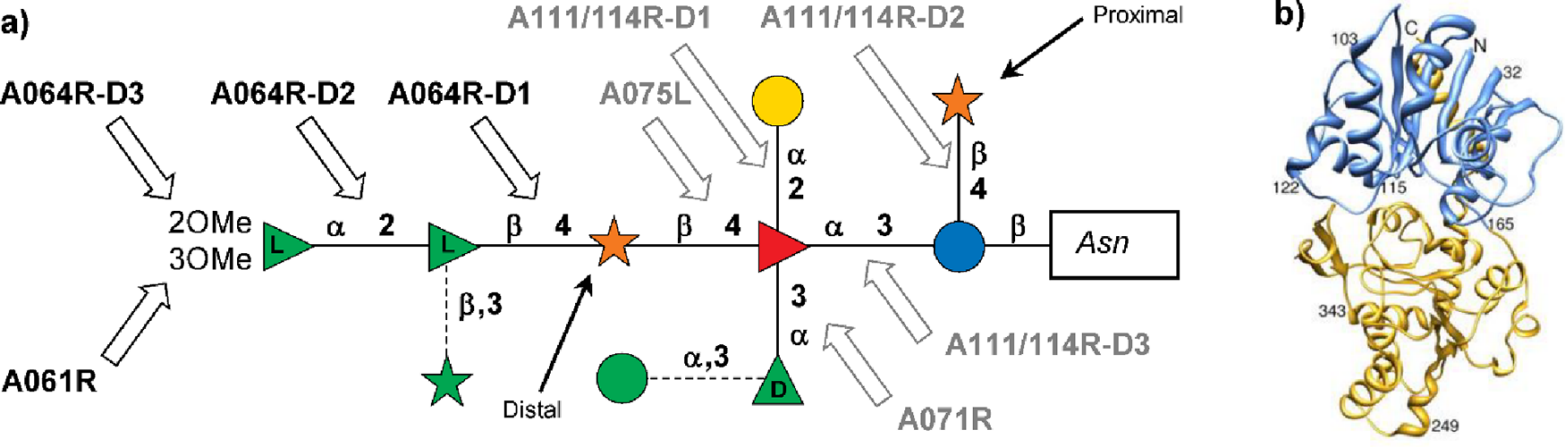
Comprehensive view of PBCV-1 GTs. (a) Structure of PBCV-1 *N*-glycan with known and predicted GTs activities. Text in black indicates that the activity has been experimentally validated, otherwise (text in gray) the activity was deduced by indirect evidence. (b) Crystallographic structure of B736L (PDB 3OY2) from the chlorovirus NY-2A, which shares high conservation with A546L of PBCV-1, whose activity is yet to be uncharacterized.. Reproduced with permission from ref (213). Copyright 2010 American Society for Microbiology.

Hence, these experiments determined a total of four activities by analyzing two different proteins. More importantly, the original predictions of A064R activity were correct and defined the first viral encoded enzyme involved in the synthesis of its own glycan. The three activities of A064R build the terminal part of the PBCV-1 *N*-glycan (Figure 15), and it should be noted that this enzyme is one of the few described to date that has three transferase domains, two of which are GTs.^141,211^ Once A064R has synthesized the terminal part of the *N*-glycan, A061L completes the decoration of the ultimate α-l-Rha with the second methyl group (Figure 15).

##### 3.3.2.3 PBCV-1 Proteins A075L, A071R, and A111/114R

Regarding the other putative GTs mentioned in section 3.3.2.1, there is no experimental evidence to support their role in the synthesis of the Vp54 glycan. However, by combining in silico analyses with the structural determination of other chloroviruses *N*-glycans, initial predictions can be made.

###### Gene *a075l*

The genome of PIL6, a member of the antigenic class D (Figure 13), has a large deletion from gene *a014r* to *a078l*, and its *N*-glycan is restricted to the conserved core structure, suggesting that the gene encoding the enzyme involved in the addition of the distal d-Xyl is in this region. In parallel, the distal Xyl is also missing in all class C antigenic variants (E1L3 and E11, Figure 13), which all have mutations in the *a075l* gene.^143^ These observations suggest that A075L is the xylosyltransferase that links d-Xyl to l-Fuc (Figure 15), although it was previously annotated as exostosin, a glycosyltransferase involved in heparin/heparan biosynthesis.^212^ This hypothesis is further supported by the analysis of the other chloroviruses, whose MCP glycan structures have been determined (Figure 9).^179,191,192^ Indeed, all these chloroviruses encode orthologues of the PBCV-1 A075L protein, and all have the distal d-Xyl attached to the l-Fuc, like PBCV-1. Further evidence suggesting that A075L links d-Xyl to l-Fuc is provided by the recent finding that chlorovirus MA-1D has a mutation in the *a075l* orthologous gene, and its *N*-glycan lacks this residue (Figure 9).^193^

###### Gene *a071R*

The *N*-glycans of the antigenic classes D (Figure 13) also differ in another monosaccharide, d-Rha. Because PIL6 (class D variant) has no mutations in its genome other than the large deletion that extends from gene *a005r* to *a077l*, the gene encoding this d-rhamnosyltransferase must be located within these genes. This gene must be conserved in other NC64A and OsyNE5 chloroviruses, which have a d-Rha attached to the l-Fuc, and it must be absent from SAG and Pbi viruses because both lack d-Rha at that position (Figure 9).^179,191,192^ The only PBCV-1 gene that fulfills all of these prerequisites is *a071r*. This gene encodes a 354 aa protein previously not annotated as a GT, but whose sequence resembles that of A064R-D2, which also escaped from in silico analysis due to a lack of homology with any known GT. Accordingly, A071R should be the GT that attaches d-Rha to *O*-3 of l-Fuc (Figure 15).

###### Gene *a111/114r*

This gene is conserved in all the sequenced chloroviruses and PBCV-1 variants. The gene product is a 860 aa protein that has been proposed to be involved in the synthesis of the conserved four-sugar oligosaccharide core shared by all chloroviruses studied to date. This prediction has been supported by a combination of bioinformatic and preliminary biochemical analysis, which indicated that A111/114R, like A064R, is a three-domain GT likely involved in the attachment of three of the four monosaccharides of the core *N*-glycan.^147^ HHpred analysis combined with the three-dimensional protein models built by Phyre2, suggested that the *N*-terminal domain (from 1 to 260 aa, A111/114R-D1) is a galactosyltransferase, the central domain (261–559 aa, A111/114R-D2) a xylosyltransferase, and the C-terminal domain (560–860 aa, A111/114R-D3) a fucosyltransferase (enzymes characteristics are reported in Table 2). Domains 2 and 3 both have the DXD motif necessary to bind bivalent cations.

The intact PBCV-1 A111/114R protein and each domain were expressed as fusion proteins with a maltose-binding protein (MBP) tag at the N-terminus, and bioluminescent UDP/GDP-Glo assays were performed on the recombinant proteins to detect free UDP/GDP released by GT-mediated hydrolysis of the sugar nucleotides.^147^ These experiments revealed the preferred nucleotide donor for each domain: UDP-α-d-Gal, UDP-α-d-Xyl, and GDP-β-l-Fuc for A111/114R-D1, -D2, and -D3, respectively (Figure 15). These results agree with the HHpred analysis for the function of each domain and also determined their correct boundaries, that, like A064R-D2, were not accurately predicted in silico. Currently, all these preliminary data remain to be experimentally validated.

###### Other PBCV-1 GTs

Not much is known about the other predicted GT proteins: A301L (241 aa), A219/222/226R (677 aa, putative GT2), A473L (517 aa, GT2), and A546L (396 aa, a GT-B type). We can speculate that among them one should be involved in the linkage of Glc to Asn or to a lipid carrier (this is unresolved). In addition, we are still missing the GTs that link Man to d-Rha and Ara*f* to l-Rha.

The PBCV-1 A546L protein presents 81% sequence identity with a predicted GT, B736L from chlorovirus NY-2A (405 aa) whose crystallographic structure is available.^213^ Out of the tested nucleotides, GDP-α-d-Man bound best to the protein, and B736L crystals were obtained both with and without GDP-α-d-Man (PDB 3OY7 and 3OY2, respectively). The structure has two Rossmann-like folds (from 1 to 163 aa and 164 to 355 aa) separated by a cleft, exhibiting the typical GT-B type structure. Residues 356 to 392 formed a long α-helix that spanned both the N-terminal and the C-terminal domains, and no metal ions were visible in the cleft of B736L, despite Mg^2+^ cations being added to the crystallization buffer. Structural comparison of B736L with other GDP-α-d-Man binding GTs indicated that crucial amino acids in the catalytic site were preserved. These results led to the proposal that B736L enzyme is a mannosyltransferase. However, because the *N*-glycans attached to NY-2A MCP lack Man (Figure 9), this suggests that either: (i) the acceptor substrate might not be the virus MCP but some other protein, or (ii) that there is another preferred sugar nucleotide donor, or (iii) the enzyme is not functional.

#### 3.3.3 *Megavirinae* GTs: a Focus on APMV GTs

As mentioned previously, several mimiviruses have been analyzed for glyco-related genes, and these studies revealed that most of them are organized in clusters.^87^ The presence of genes encoding putative GTs next to those encoding sugar nucleotides of the monosaccharides present in the fibrils (section 3.1.2.5, Figure 8) led the authors to speculate that these enzymes were involved in the formation of the glycans that decorate the fibrils. Currently, APMV is the only *Mimiviridae* member for which the glycan structures have been determined (section 3.2.2),^65,194^ and this information has been used to determine the role of the GTs identified in the cluster. Two additional GTs have been characterized, L230 and R707 (enzymes characteristics are reported in Table 2),^24,25^ located outside the glycogene cluster and with no evident relationship with the structure of the fibril glycans. In 2011, Luther et al.^24^ studied the gene *l230*, which encodes a bifunctional enzyme with the N-terminal domain (1–194 aa) bearing a GT activity and the C-terminal domain (537–895 aa) with a lysin-hydroxylase activity, which can modify collagen. Collagen is an animal protein, and homologues have been found in APMV: L71, R196, R239, R240, R241, L668, and L669, all of which contain the Gly–X–Y repeat typical of collagenic proteins, where X and Y are often Pro and Lys, respectively. However, these viral proteins were rather low in proline content in contrast to their mammalian counterparts.

The N-terminal domain of L230 presents 27.7% amino acids identity (61% similarity) over 285 amino acids with a human GT, GLT25D1, which adds Gal to collagen.^214^ The C-terminal part of the protein has 35% identity (68% similarity) over 388 amino acids, with the human lysyl hydroxylase PLOD1 enzyme. Both activities of the viral enzyme were experimentally demonstrated, and mutagenesis experiments showed that each domain activity was independent of the other.

**Figure 16:**
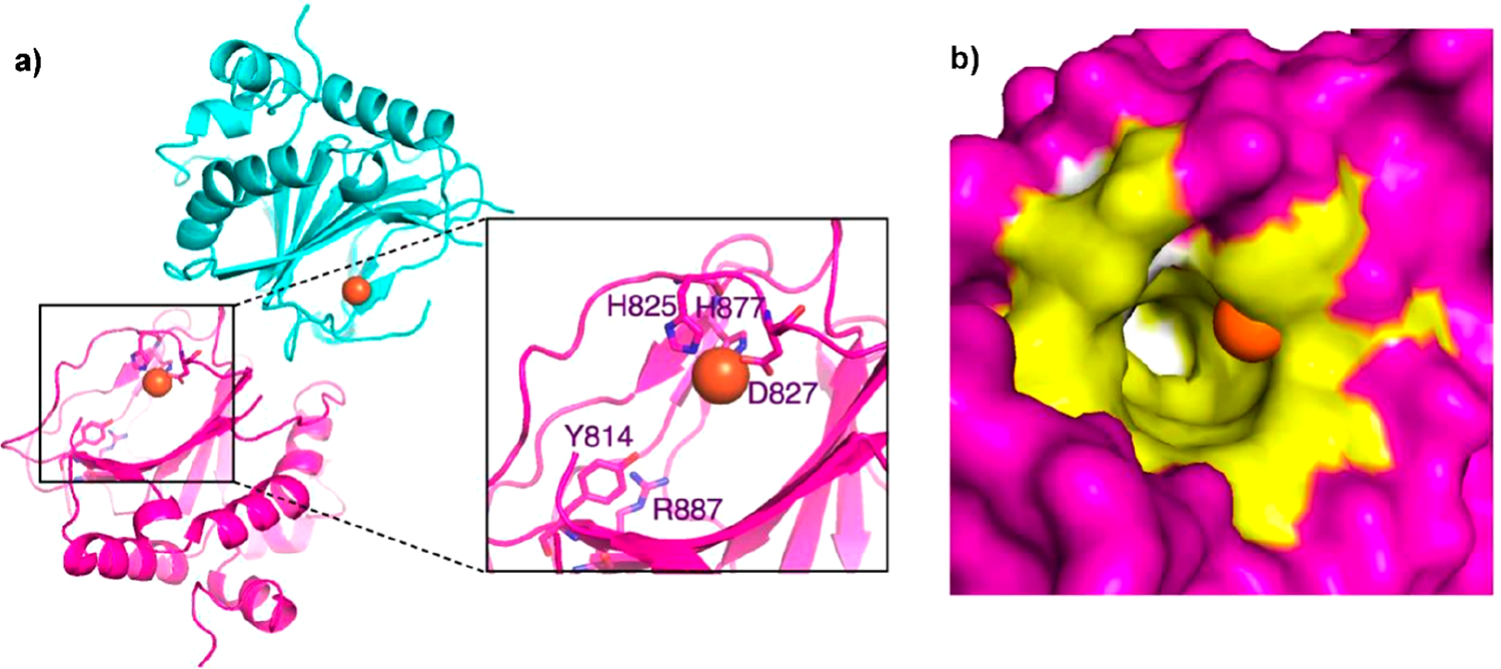
Crystallographic structure of the C-terminal domain of L230. L230 is a bifunctional enzyme with GT and lysyl hydroxylase activities. (a) Representation of the secondary motifs of L230 C-terminal domain and its arrangement as a dimer. The small inset highlights amino acids involved in Fe^2+^ cation (orange) coordination. (b) Surface representation of the L230 active site: the residues inside the binding pocket conserved in the human enzyme LH2 are in yellow and otherwise in white. Residues outside the binding pocket are in purple. Reproduced with permission from ref (153). Copyright Springer Nature.

The residues ^825^Hys (histidine) and ^825^Asp in the C-terminal domain of L230 were essential for lysyl hydroxylase activity. Experiments performed with different peptide substrates showed that this domain could hydroxylate Lys in both human- or viral-like sequences, with the peptide derived from the APMV R240 protein being preferred above the others. The crystallographic structure of this domain (680 to 895 aa) was determined (PDB 6AX6, Figure 16).^153^ It formed a dimer with an overall fold resembling that of Fe^2+^ and 2-oxoglutarate (2-OG) dependent oxygenases, like human LH2, which is also a bifunctional enzyme.^153^ The typical residues of the oxygenase domain involved in Fe^2+^ coordination (^825^Hys, ^827^Asp, and ^877^Hys) and in the 2-OG binding (^814^Thr and ^887^Arg) were conserved and their mutation completely abrogated the protein activity. Their dimer formation involved both ^873^Leu and Fe^2+^ ions, as L873D mutation of the residue coordinating Fe^2+^, or demetalation, prevented dimerization. Dimer formation increased the ability of the enzyme to bind the collagen chains, and it was required to hydroxylate the substrate.

Regarding the GT activity, L230 added Glc instead of Gal to the substrate and it also had a certain permissivity in terms of substrate, as it was active both on human- and viral-like peptides, as observed for the hydroxylase domain. However, mutation experiments meant to define the key amino acids for catalysis, did not produce clear results. Mutation of ^106^Glu, ^107^Asp, ^108^Asp (the putative DXD motif involved in the coordination of the cation with the sugar-nucleotide), ^78^Leu, ^80^His, ^95^Asp, ^99^Asp, and ^131^Asp, all produced unstable proteins that could not be studied.

The activity of full length L230 protein was validated on peptides derived from both viral collagen-like protein L71 and human collagen, with the result that L230 reacted with the Lys residues in all the substrates and added a Glc unit onto the newly formed hydroxylysine units.^24^ Of note, analysis of the APMV particles confirmed the presence of hydroxylated lysine residues in the collagenic proteins, albeit not their glucosylation.^24^

L230 was the first collagen-glycosylating enzyme found outside the animal world, and its GT activity is specific for Glc and not for Gal, the sugar attached to the collagen in all animal sources. The attachment of Glc to viral collagen makes it unique, thus raising the question on the role it could have played in the evolution of collagen biology.

In 2016, a second GT from APMV, R707, was characterized.^25^ A BLAST search revealed that R707 is a homologue of the animal glycogenin-1, with which it shares 26% amino acid sequence identity and 42% similarity, which increased to 58% identity when focusing on the amino acid residues in the GT catalytic pocket, thus suggesting a similar function. Indeed, a model of R707 generated with Phyre using glycogenin as reference confirmed that R707 shared the typical glycogenin DXD motif (^101^Asp, ^102^Leu, and ^103^Asp). Additional experiments demonstrated that the protein coordinated UDP-α-d-Glc in the presence of Mn^2+^. The mutation of ^103^Asp or of ^232^Lys, two residues important for UDP-α-d-Glc recognition, led to an inactive enzyme.

Interestingly, R707 could use both pNP-α-Glc and pNP-α-Xyl as acceptors, although it preferred the first. Using pNP-α-Glc produced disaccharides with Glc linked via α-(1→6), β-(1→6), or α-(1→4) linkages. Thus, R707 showed a limited selectivity in terms of site of attachment (4 or 6) and of configuration of the newly formed glycosidic linkage (α or β), with this last feature being unique among all the GTs studied.

In contrast to glycogenin, R707 cannot self-glucosylate even though it has the same conserved Tyr residue; mutation of this residue (^215^Tyr) had no effect on the stability and activity of the protein. It was therefore speculated that R707 was not involved in glycogen formation as glycogenin self-glycosylates itself at one Tyr residue to prime the synthesis of the polymer. Indeed, APMV uses energy from the host, and it would not be expected that this viral enzyme would be involved in energy storage. Instead, R707 could play a role in protein glycosylation by synthesizing the linear polymers of Glc identified in the *O*-glycans (section 3.2.2.1).^194^

### 3.4 Glycosyl Hydrolases (GHs) and Lyases (PL)

An important part of the infection process is how viruses interact with the host cells; in particular, those hosts that have a carbohydrate-rich cell wall. This interaction is required as the point of entry of the virus to start the infection process as well as during the release of new progeny at the end of it.

**Figure 17:**
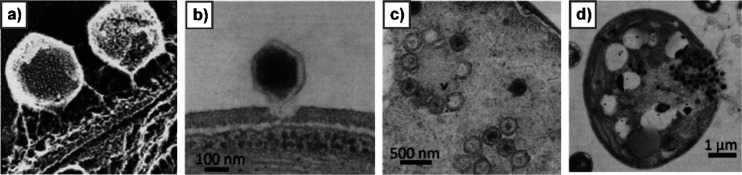
Electron microscopy images detailing some of the phases of PBCV-1 infection. (a) Attachment of the virus on the host cell (infection time zero). Reproduced with permission from ref (216). Copyright 1991 American Society for Microbiology. (b) Degradation of the wall at the site of attachment, typically 1–3 min PI. Reproduced with permission from ref (215). Copyright 1984 Elsevier. (c) PBCV-1 particles in the so-called virus assembly centers or viral factories in the cytoplasm of the host, ∼5 h PI., the DNA containing particles are dark, otherwise the capsids are empty. Reproduced with permission from ref (217). Copyright 1986 Elsevier. (d) Lysis of the host cell plasma membrane and cell wall and release of progeny viruses at ∼7 h PI. Reproduced with permission from (218). Copyright 1981 Elsevier.

As demonstrated for chloroviruses, these two check points target the glycans of the host, which are degraded by the action of some viral enzymes. Indeed, chlorovirus PBCV-1 infects the *Chlorella* NC64A host by (i) attaching to the cell wall (Figure 17a), (ii) degrading the wall at the point of attachment (Figure 17b), (iii) releasing viral DNA into the host cytoplasm while leaving an empty capsid at the top of the cell wall, (iv) replicating in the assembly centers or viral factories (Figure 17c), and (v) breaking the host cell wall to release the neosynthesized virion progeny (Figure 17d).^215,216^

Regarding the degradation of the host cell wall, this process occurs during the chlorovirus entry (Figure 17b), and again during the virion release (Figure 17d). In the first case, the enzyme(s) is already packaged in the capsid to make a breach in the host (Figure 17b); it is subsequently produced at a certain time during the infection process to break the cell wall and to let the particles out of the host (Figure 17d). Accordingly, these cell wall degrading proteins have different roles and are not expressed at the same PI time. Their production is not continuous during the infection process, and it is tightly controlled because if it occurs too early, only incomplete virus particles would be released, which would lead to its demise.

A characteristic of virus-sensitive *Chlorella* microalgae is a rigid cell wall with a complex architecture likely involving different types of glycans.^219^ The overall sugar composition of the cell wall is 7–15% glucosamine and also contains uronic acids and neutral sugars. Chloroviruses penetrate this cell wall, and early hypotheses suggested that enzymes capable of degrading polymers of glucosamine, like chitosan and chitin, were involved in the viral infection process. Overall, in silico analyses of the chloroviruses genomes led to the discovery of both hydrolases and lyases able to degrade glycans, and the studies reported in the next sections focus on those enzymes that have been experimentally characterized.

#### 3.4.1 Chlorovirus Chitinase and Chitosanase

In silico analyses of chloroviruses indicated that chitinase and chitosanase genes are widespread, suggesting that they are important for viral replication. Chitinolytic enzymes are classified in the CAZY database into three different families, namely GH 18, GH 19, and GH 20, depending on their overall fold and mechanism of action.^220,221^ GH families 18 and 19 act on chitin, while GH family 20 includes enzymes that catalyze the cleavage of GlcNAc from the disaccharide chitobiose or glucosamine from glycoconjugates. Regarding GH families 18 and 19, members of the latter are mostly from plants, while those of the former are from different sources (bacteria, fungi, insects, and plants).

As for chitosanases, these enzymes catalyze the endohydrolysis of chitosan and belong to families GH 46, GH 75, and GH 80 according to CAZY database.^221,222^ Members of the GH46 family are the most characterized. They are found in eubacteria and can be divided into five clusters, A to E. Chloroviruses encode chitosanases belonging exclusively to cluster C,^223^ and they all have an additional N-terminal domain of unknown function that is unique to chloroviruses. It can then be assumed their function is closely linked to a particular trait of the biology of these viruses.

##### 3.4.1.1 PBCV-1 Chitin and Chitosan Hydrolases

The PBCV-1 gene *a181/182r* encodes a large protein (A181/182R, 830 aa, Table 2), and in silico analyses found that it was composed of three distinct domains.^149^ An N-terminal domain or domain 1 (aa 6–101) has homology to cellulose-binding domains, with its best homologue in *Cellulomonas fimi* (32% over 74 aa). The closest homologue of domain 2 (aa 101–411) corresponds to an endochitinase from *Aeromonas* sp. (36% identity over 168 aa). Domain 3 (aa 553–825) has 35% identity over 201 amino acids with a protein from *Ewingella americana*. Domains 2 and 3 are separated by a Pro-rich linker of 140 amino acids.

Originally, it was thought the two chitinases were encoded by two separate genes, *a181r* and *a182r*,^224^ but later reevaluation of the genomic sequence demonstrated that they were part of a single gene.^149^ Of the two recombinant proteins, only A181R had detectable chitinase activity suggesting that the two domains could function independently. The two chitinase domains share 40% sequence identity, suggesting that they were the result of a duplication event, and both domains were classified into the chitinase family 18. Biochemical assays on the recombinant full-length protein, showed its optimum pH value was between 4 and 6 and was active up to 50 °C. Transcriptomic analysis revealed that *a181/182r* was expressed early, at 30 min PI and that its transcription was maintained throughout the entire replication cycle, although the protein was not packaged in the virion.

In contrast to A181/182R, A260R is a single domain chitinase within the GH family 18,^221^ with 32–36% amino acid identity with bacterial and fungal chitinases (Table 2). This protein is endowed with both endo- and exochitinase activities and has an optimum pH between 5 and 9, up to 50 °C. Transcription of *a260r* started at 60 min PI, reached its maximum at 90–120 min PI, and it was maintained until the host cells lysed. Like A181/182R, A260R was not packaged in neosynthesized PCBV-1 virions.^109^

The A292L protein is a chitosanase that belongs to the GH family 46,^221^ with 97% amino acids sequence identity with the chitosanase from chlorovirus CVK2,^138^ and about 29% identity with bacterial chitosanases (Table 2). The enzyme had a pH optimum value in the range 5 to 8 and was active up to 50 °C.^149^*a292l* exhibited the same expression pattern as *a260r*; it first appeared at 60 min PI and remained until the lysis of the cells with a maximum production at 90–120 min PI. Like the two PBCV-1 encoded chitinases, A292L is not packaged in the virion.^109^

##### 3.4.1.2 CVK2 Chitin and Chitosan Hydrolases

Like PBCV-1, chlorella virus strain CVK2 also encodes chitinase (vChti-1), and chitosanase (vChta-1) enzymes (Table 2).^138,139^ vChti-1 (836 aa) has 98% identity with A181/A182R from PBCV-1, with two chitinase domains assigned to the GH family 18. The first domain (144–413 aa) has the most similarity with *Saccharopolyspora erythrae* (30% identity), while the second domain (aa 560–836) has a match (35%) with the enzyme from *Ewingella americana*. Like A181/182R of PBCV-1, it was suggested that the two chitinase domains of vChti-1 are the result of a duplication event in their common ancestor. Such events were also proposed for the hyperthermophile archaeon *Pyrococcus kodakaraensis* KOD1 and for the plant pathogenic bacterium *Flexibacter* sp. FL824A.^225^

Regarding the timing of transcription, analysis of the viral RNA during the infectious cycle revealed that *vChti-1* was a late gene because it appeared after 120 min PI and remained up to 360 min PI, gradually decreasing afterward. Like its orthologue A181/182R in PBCV-1, the protein was not packaged in the virions, and it was proposed that vChti-1 chitinase was not involved in the initial infection events, while its presence in the host cytoplasm served to digest the host cell wall to enable viral release at the final stage of infection.^139^

As for the chitosanase, vChta-1 (328 aa) has 95% sequence identity with PBCV-1 A292L, with a predicted MW of 36.8 kDa. The recombinant protein was used to produce antibodies to monitor the presence of this protein in virus-infected cells.^138^ This identified two proteins of 37 and 65 kDa, respectively, which were detected at 240 min PI. The larger protein was later packaged in the virion, while the other remained in the cell lysate. In-depth analysis of the CVK2 genome suggested that these two chitosanases arose from the same gene, and it was hypothesized that the *a292l* stop codon was fused with the next downstream gene by a read-through mechanism to produce the 65 kDa protein. It was proposed that the 65 kDa protein packaged in the virion was involved in cell wall degradation at the beginning of infection and the 37 kDa enzyme produced late during infection contributed to cell lysis at the final step of infection. However, subsequent experiments indicated that the 65 kDa protein was not packaged in the virion. Interestingly, *Chlorella* NC64A cells treated with both chitinase (vChti-1) and chitosanase (vChta-1) did not show relevant morphological changes, indicating that additional enzymatic activities are required to digest its complex cell wall.^139^

#### 3.4.2 PBCV-1 β-Glucanase

PBCV-1 also encodes a functional β-(1→3)-glucanase,^144^ A094L (Table 2), sharing 26–30% sequence identity with GH family 16 of endo-β-(1→3)-glucanases from several bacteria, with conservation of residues important for activity: ^234^Glu, ^236^Asp, and ^239^Glu. The recombinant protein A094L expressed in *E. coli* was able to hydrolyze laminarin, a linear polysaccharide made of β-(1 → 3) glucose units, better than other β-glucans, like lichenan and barley β-glucan, both having mixed β-(1→3)/β-(1→4)-linkages. A094L was active over a pH range of 4–10, with an optimum at about 8. Its activity increased from 25 to 65 °C, and was inhibited by 1 mM silver, cobalt, copper, or manganese ions.^144^

Interestingly, this gene is expressed 15 min PI and disappeared after 60 min PI, so that it was classified as an early gene. For this reason, no role during the late phase of viral infection was proposed.^144,210^ Accordingly, this protein was not predicted to be involved in breaking down the host cell wall. Instead, it was hypothesized that it might degrade β-glucans stored by the host to produce the energy required for viral replication. Indeed, laminarin is a common reserve polysaccharide for many algae, although there is no experimental evidence that *Chlorella* NC64A also stores this polysaccharide. The search for this gene in other chloroviruses revealed it was not conserved,^144^ suggesting it is not essential for their replication, although many of the viruses devoid of A094L homologue contained another gene, *bgl2*, also predicted to encode an endo-β-(1→3)-glucanase.^208^

#### 3.4.3 CVN1 Alginate Lyase

Chloroviruses can remodel the host cell wall also by using polysaccharide lyases (PL), as described for CVN1.^140^ Screening of the CVN1 viral DNA library cloned into an *E. coli* expression vector, with the resulting *E. coli* strains assayed on *Chlorella* NC64A, identified the CL2 protein (333 aa, Table 2) encoded by *orf2* as capable of lysing different *Chlorella* species. The recombinant CL2 protein had 65% sequence identity with PBCV-1 A215L, a protein of unknown function, with a weak homology (20% sequence identity) with the mannuronate lyase from the mollusk *Turbo cornutus*.^140^ Indeed, CL2 was a PL able to cleave alginate, an acidic polysaccharide produced by some algae and bacteria made by d-mannuronic acid and l-guluronic acid, in proportions that depend on the source used. The CL2 protein had an optimal pH of 10.5 and required Ca^2+^ ion for activity as the addition of EDTA to the reaction mixture inhibited its activity. CL2 was not active on other polysaccharides such as chitin, chitosan, cellulose, or pectin. Regarding its antialgal activity, CL2 degraded the cell wall of *Chlorella* sp. NC64A, *Chlorella vulgaris* C-27, and *Chlorella vulgaris* C-207, suggesting all these green microalgae might have alginate as a component of their cell wall.

#### 3.4.4 CVK2 Alginate Lyase

By using an approach similar to that described for chlorovirus CVN1, it was found that chlorovirus CVK2 also encoded a PL enzyme. The gene encoding vAL-1 (349 aa, Table 2) has 93% sequence identity at the nucleotide level, with the gene encoding CVN1 alginate lyase CL2,^140^ and 97% sequence identity with the gene encoding the PBCV-1 A215L, an uncharacterized protein.^224^ The vAL-1 gene was expressed early, at 60 min PI, and gradually decreased. At the end of the infection cycle, the vAL-1 protein was not incorporated into the viral particles but remained in the cell lysate, suggesting that its role was limited to the digestion of the cell wall prior to virion release.

Biochemical assays with the recombinant vAL-1 protein demonstrated that it had strong cell-lysing activity on different *Chlorella* species and that it was an alginate lyase.^136,226^ Interestingly, vAL-1 had maximum activity at alkaline pH (about 10) and was minimally active at neutral pH, even though the enzyme could only digest the algal cell wall under physiological conditions. When the protein was stored at high protein and/or low salt concentration, it spontaneously degraded into vAL-1(S), by losing its N-terminal 105 amino acids resulting in a shift in the MW from 38 to 27 kDa. This N-terminal loss included 10 repeats of a Pro–Ala–Pro–Lys sequence that appeared to play an important role in the attachment of the protein to the algal cell wall.^137^ As a result, vAL-1(S) lost the ability to degrade the algal cell wall, but its pH range of activity expanded from pH 7–10, compared to that of the native form, suggesting that the N-terminal domain regulated the lyase activity of the protein.

**Figure 18:**
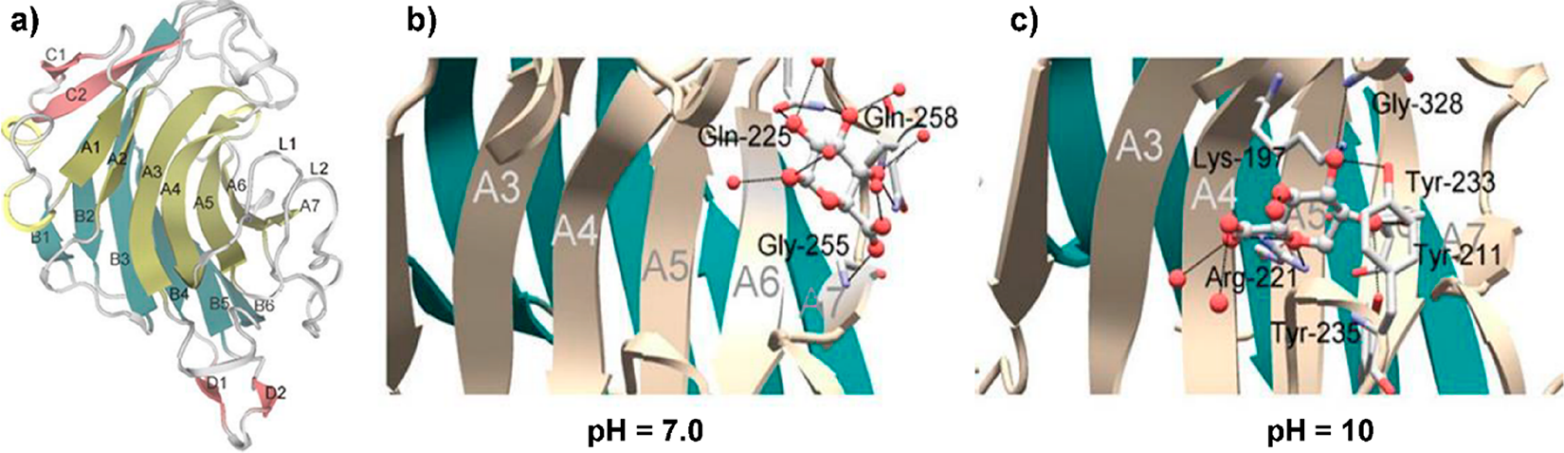
Crystal structure of vAL-1(S) alginate lyase from chlorovirus CVK2. (a) Cartoon representation of the overall fold of the protein without ligand (PDB 3GNE). (b) Details of the CL2 complex with GlcA at pH 7.0 (PDB 3A0N), and (c) at pH 10.0 (PDB 3IM0). Depending on the pH, GlcA bound outside (pH 7, b) or inside (pH 10, c) the cleft located nearby A3–6 strands, as detailed by the interactions in place between the monosaccharide and the amino acids of the protein, that are not same in the two cases. Adapted with permission from ref (137). Copyright 2009 Elsevier.

PLs can be endo- or exoenzymes, and vAL-1(S) could switch from one to the other activity as a function of pH, with endolytic activity at neutral pH, and exolytic activity at alkaline pH.^137^ The X-ray crystal structure of vAL-1(S) (PDB 3GNE) determined that the enzyme belonged to the family 14 of PL lyases and had a β-jelly roll fold composed of two antiparallel β-sheets, named A and B, and two minors sets, named C and D (Figure 18a). vAL-1(S) was studied in complex with GlcA, an acidic monosaccharide used in place of mannuronic or guluronic acids, which were not available for the experiments. The vAL1(S)– GlcA complex at different pH values revealed that GlcA was bound outside of the active site cleft at neutral pH (Figure 18b) and in the cleft at alkaline pH (Figure 18c). These two different arrangements of the complex depended on the charge status of the different groups of the protein and of the ligand, and regulated the endo/exo activity of the enzyme in a unique way compared to other enzymes of the same class.^137^

#### 3.4.5 PBCV-1 A561L, a putative alginate lyase

As noted above, chlorovirus PBCV-1 encodes five functional polysaccharide degrading enzymes, two chitinases, a chitosanase, a β-(1 → 3)-glucanase, and an alginate lyase. However, PBCV-1 does not use any of them to digest the host chlorella cell wall for virus entry because none of these enzymes are packaged in the virion. Re-examination of the 148 virus-encoded proteins that are packaged in the PBCV-1 virions led to the identification of A561L (649 aa, Table 2), a protein expressed late during the viral infection cycle.^150,210^

In silico analyses found that this protein is made of four domains.^150^ The first is an N-terminal transmembrane domain 1 (69 aa; A561L-D1) homologous to a transporter protein encoded by *Listeria kieliensis*. The second domain, A561L-D2 (from aa 70 to 310), resembled the TolA protein from *Trichomonas vaginalis*, a protein similar to the Gram-negative bacterial Tol proteins that are involved in the stability of the outer membrane. The third, A561L-D3 (from aa 311 to 407), had homology with the LEA 2 domain-containing protein encoded by *Selaginella moellendorffii*, an important protein for plants as it is involved in the hypersensitive response to plant pathogen infection. The last, C-terminal domain (A561L-D4, aa 408–649) shared 32% sequence identity over 211 amino acids (50% similarity) with the C-terminal domain of A215L, a close homologue of vAL-1, the chlorovirus CVK2 alginate lyase (section 3.4.4).^137^

Accordingly, the Phyre2 model of A561L-D4 corresponded to alginate lyases in family 14 (87% coverage, 100% confidence) with the *Aplysia kurodai* enzyme used as a reference (PDB 5GMT). A561-D4 was produced as a recombinant protein in *E. coli*, and when assayed on chlorella ghost cells (algal cells without chlorophyll by treatment with methanol) dyed with calcofluor-white, it induced the release of the dye, thus demonstrating the protein ability to degrade the algae cell wall. Optimal activity was at 37 °C and at pH 7.0–8.0; while the activity was not cation dependent, it was enhanced by Ca^2+^ and Mg^2+^ cations.

A561L extracted from PBCV-1 virions was shown to degrade the cell wall of different *Chlorella* isolates, thus demonstrating it was not host-specific. The *a561l* gene was conserved in all the 52 chloroviruses that have been sequenced, with domains D2 and D4 being the most conserved between all viral strains. The biochemical activity of A561L-D4 has not been determined, although its strong homology with the other proteins suggests that it has alginate lyase activity. Therefore, this enzyme is believed to be responsible for degrading the host cell wall during virus infection.

## 4 Chemical Synthesis of Glycans Inspired from Giant Viruses

Given their unusual structures, viral glycans have attracted the attention of synthetic chemists. To date, efforts to synthesize these molecules have focused on two areas: (1) derivatives of viosamine, a component of APMV glycans, and (2) the complex *N*-glycans produced by chloroviruses.

### 4.1 Viosamine

**Figure 19:**
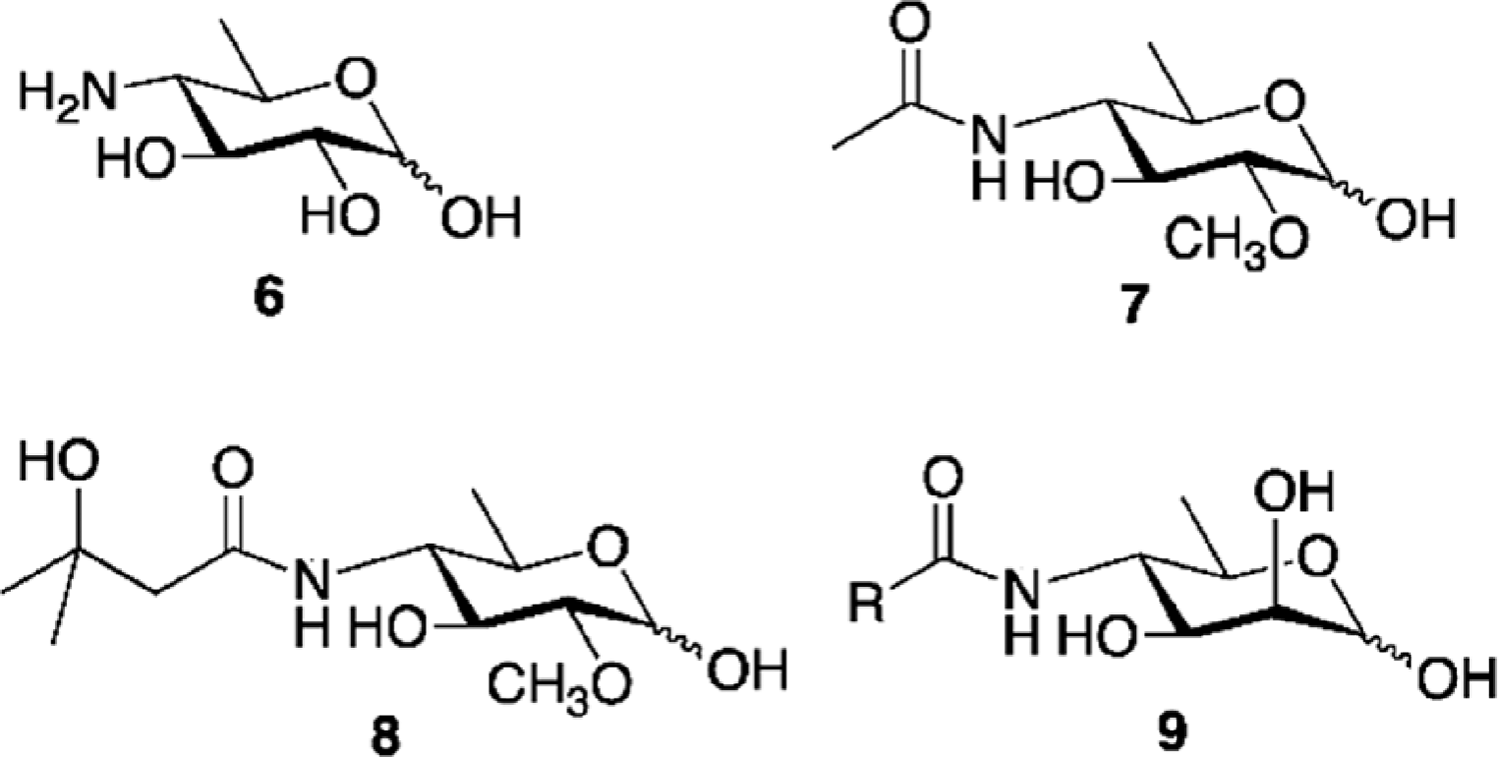
Structures of Vio4N and related derivatives from viral and bacterial sources. Viosamine (**6**); 2OMeVio4NAc (**7**), the Vio4N derivative found in APMV glycan poly_2; anthrose (**8**), a Vio4N unit methylated at *O*-2 and with a complex acyl group at *N*-4; *N*-acyl-perosamine (**9**), the C-2 epimer of Vio4N.

The presence of viosamine (**6**, Figure 19), in its *N*-acetylated, 2-*O*-methylated form (**7**), was recently identified in APMV.^60^ However, this monosaccharide, with different acyl groups, had been identified earlier in bacteria.^227,228^ Prominent among these derivatives is anthrose (**8**), a focus of many synthetic investigations.^229−236^ Anthrose is a monosaccharide found in the *Bacillus anthracis* glycoprotein BclA,^237^ the detection of which has been suggested to have potential in diagnosing anthrax.^238^ In addition, *N*-acylated forms of perosamine (**9**), the C-2 epimer of viosamine, are found in a number of microbial glycans.^239,240^ This section will focus not only on previous syntheses of viosamine but also anthrose because straightforward modification of the routes used for the preparation of the latter could be applied to the viosamine derivative present in APMV glycans.

**Figure 20:**
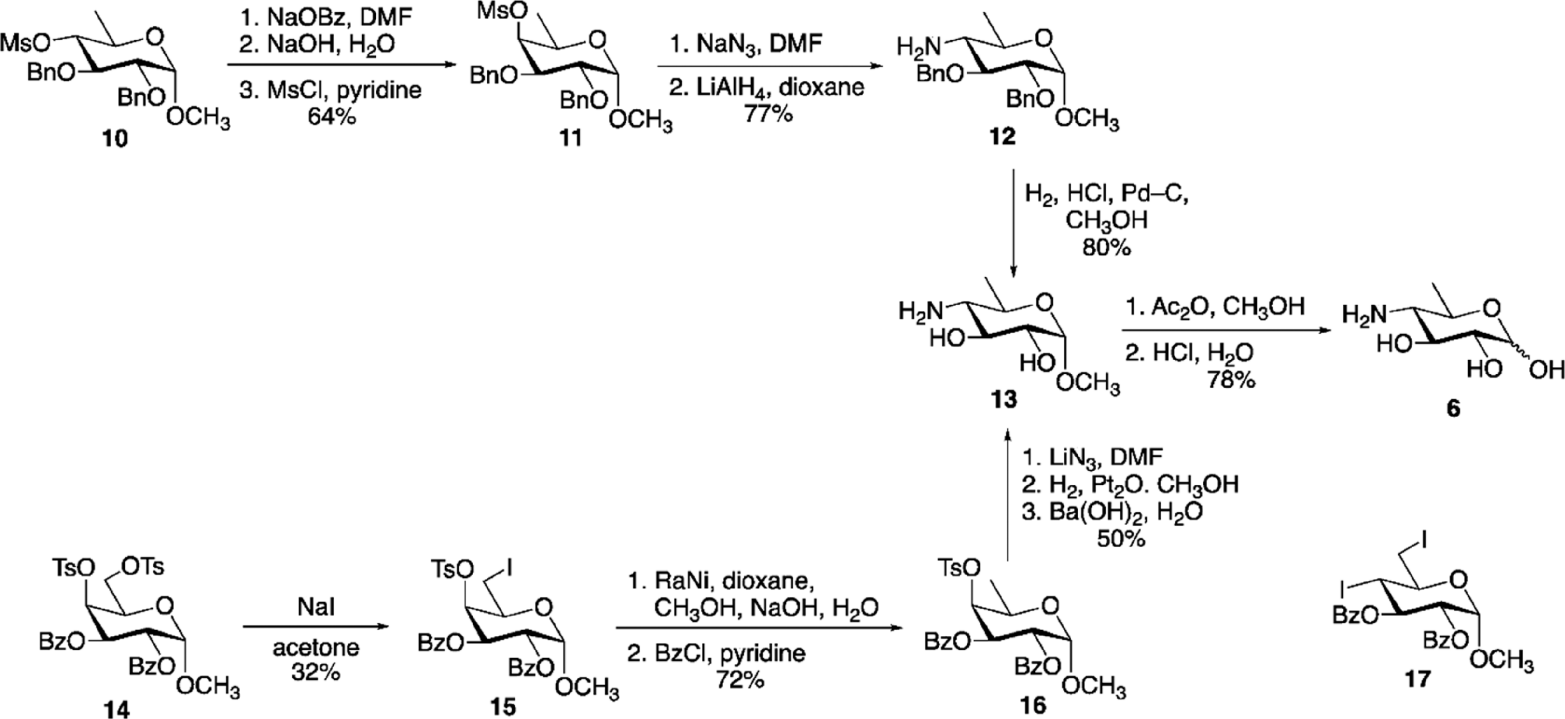
First syntheses of viosamine (**6**) by Stevens and co-workers.^241,242^

Over 50 years ago, Stevens and co-workers were the first to report the synthesis of viosamine.^241,242^ Starting from the mesylated methyl 6-deoxy-glucopyranoside derivative **10** (Figure 20), a three-step inversion, deprotection, and mesylation sequence provided a 64% overall yield of **11**. Conversion of this compound to amine **12** was carried out in 77% yield via displacement with sodium azide and reduction with lithium aluminum hydride. Hydrogenation (giving **13**), *N*-acetylation, and the acid hydrolysis provided viosamine (**6**) in 62% overall yield. An alternate, lower-yielding, approach was also reported starting from ditosyl galactopyranoside **14**. Displacement with sodium iodide led to **15**, which was reduced and benzoylated to give **16** in 23% overall yield. Azide displacement, reduction, and cleavage of the benzoyl groups provided **13** in 50% overall yield. The low yield of the conversion of **14** into **15** was due to the formation of the 4,6-di-iodo-derivative **17** in 45% yield.

**Figure 21:**
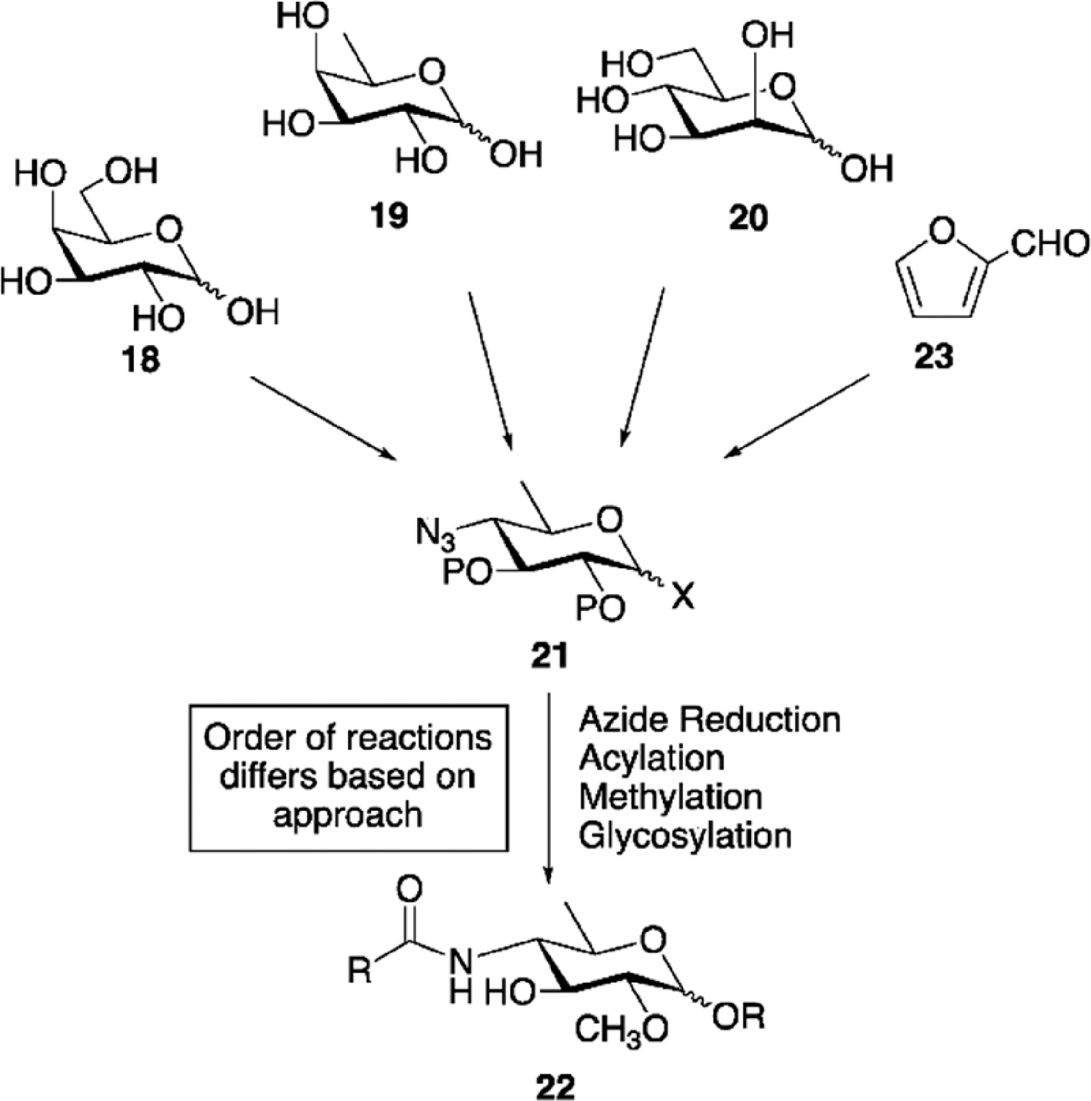
General synthetic approaches to 2-*O*-methyl-*N*-acylated viosamine derivatives.^229−236,243^

Since these early syntheses, a number of other approaches have been developed, which, for the most part, use the same general strategy: the conversion of a readily available monosaccharide to a viosamine derivative. Rather than detail all of these, the general strategies employed are instead shown in Figure 21. The common approaches have started with d-Gal (**18**),^229−231^d-Fuc (**19**),^232−234^ or d-Man (**20**)^235,236^ and, in several steps, arrived at a 4-azido-4,6-dideoxy derivative **21**. Depending on the final goal, **21** can be converted to a target molecule **22** through a combination of azide reduction, acylation, methylation, and glycosylation in an order depending on the desired molecule and/or convenience. A twist on this general approach has been reported by O’Doherty, who developed a de novo route to molecules of this type starting from furfural (**23**).^243^

### 4.2 Chlorovirus *N*-Glycans

The unique structures of chlorovirus *N*-glycans have piqued the curiosity of synthetic chemists not only as an intellectual and technical challenge but also to provide probe molecules that can be used to understand their biosynthesis and function. The major challenge in the synthesis of these molecules is the construction of the hyberbranched central Fuc residue, in which every hydroxyl group is glycosylated. Such highly branched glycans are rare in nature, and understanding how to assemble these types of systems efficiently remains relatively poorly understood. The discussion below focuses primarily on the approaches used to install this highly congested motif.

**Figure 22:**
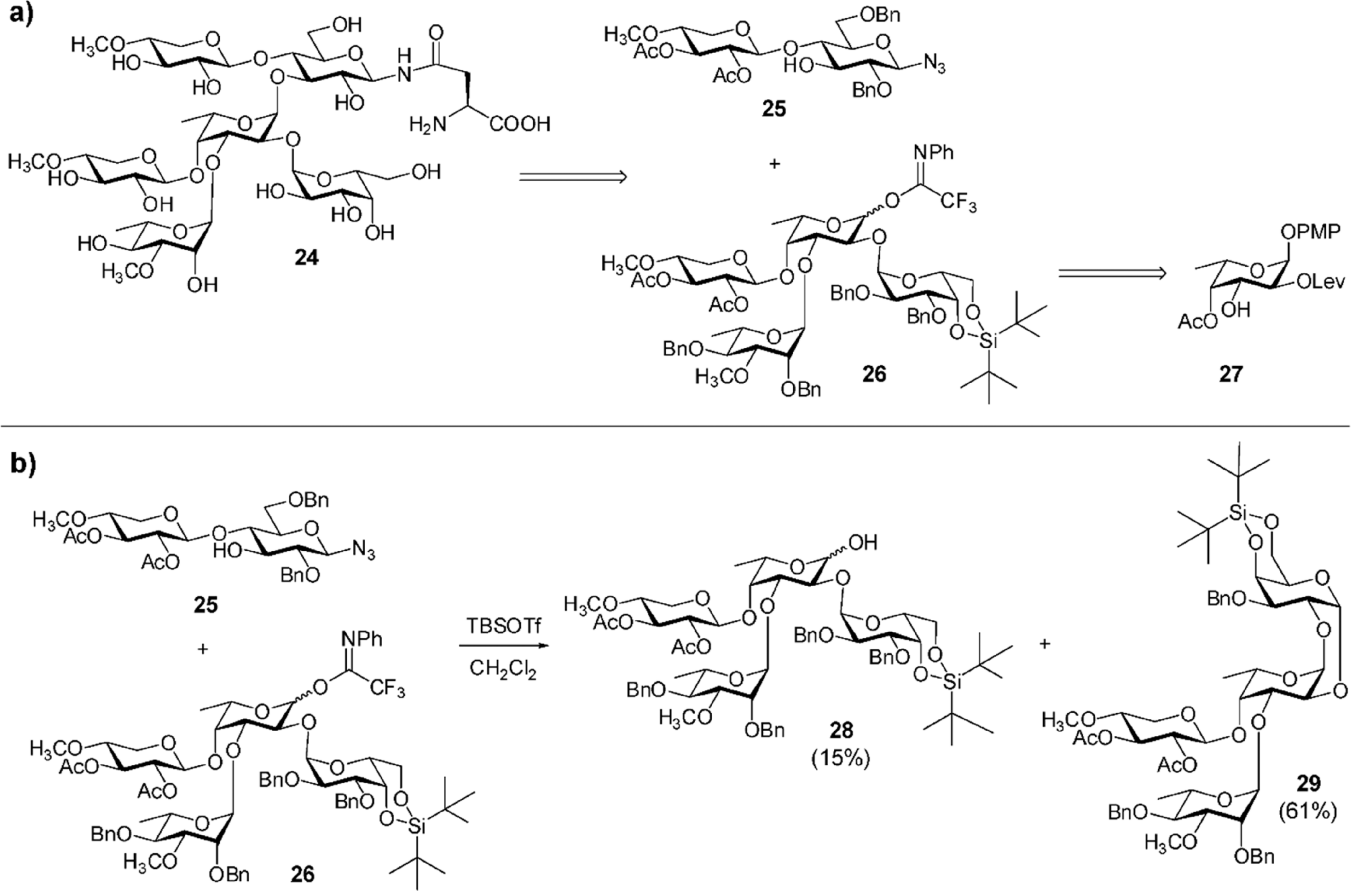
(a) High-level retrosynthetic analysis of the chlorovirus ATCV-1 *N*-glycan. (b) Attempted synthesis of the ATCV-1 hexasaccharide via a 4 + 2 coupling of **25** and **26**.^244^

The first chlorovirus *N*-glycan to be synthesized was the smallest of the structures, the hexasaccharide motif from ATCV-1 (**24**, Figure 22a or Figure 8).^244^ An initial attempt to make **24** involved a 2 + 4 coupling between the disaccharide acceptor **25** and the tetrasaccharide donor **26** as the key step. The latter of the compounds was prepared starting from the *p*-methoxylphenyl fucoside **27**, which was selectively elaborated by a series of glycosylations and deprotections and finally conversion to the activated imidate donor. The sequence used to install the monosaccharide substituents to the Fuc involved glycosylation of *O*-3, then *O*-2 and finally *O*-4.

Attempts to couple **25** and **26** (Figure 22b) failed to provide the desired hexasaccharide product; instead, two major products were generated: hemiacetal **28** (15%) and bicyclic product **29** (61%). The former was produced by hydrolysis of the donor, and the latter presumably by intramolecular trapping of the oxacarbenium ion intermediate generated from **26** by *O*-2 of the Gal residue and loss of a benzyl group. The formation of such products^245^ points to the hindered nature of the acceptor alcohol in disaccharide **25**. However, all efforts to change the outcome through the use of a different activatable group in the donor, alternative activation conditions, or a different acceptor molecule, failed to produce the desired glycosidic bond.

**Figure 23:**
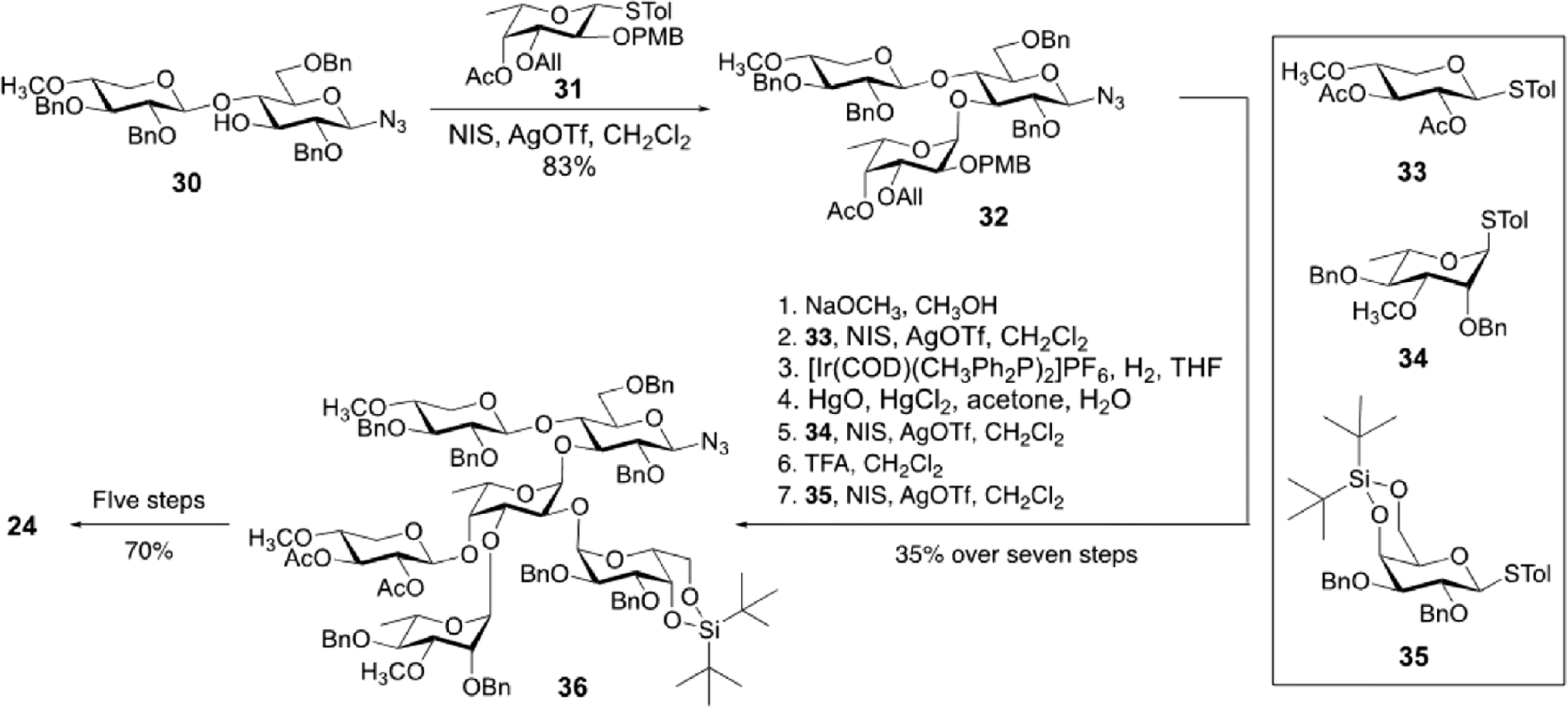
Successful synthesis of the chlorovirus ATCV-1 *N*-glycan **24**.^244^

Fortunately, the target could be assembled using a stepwise approach, the key steps of which are illustrated in Figure 23. Disaccharide alcohol **30** was assembled using appropriate monosaccharide precursors and then glycosylated with Fuc thioglycoside **31**, which contains three protecting groups that can be cleaved independently. This reaction produced trisaccharide **32** in 83% yield. The key strategic decision at this point was the order of the addition of the monosaccharides to the Fuc. A number of sequences were investigated, many of which failed. The successful route involved the “counterclockwise” addition of the substituent monosaccharides (glycosylation of *O*-4, then *O*-3 and then *O*-2) via donors **33**, **34**, and **35**, respectively. Using this strategy, the hexasaccharide **36** was produced in 35% yield over seven steps. Reduction of the azide, installation of the amino acid and then total deprotection gave the final target **24** (five steps, 70% overall yield).

**Figure 24:**
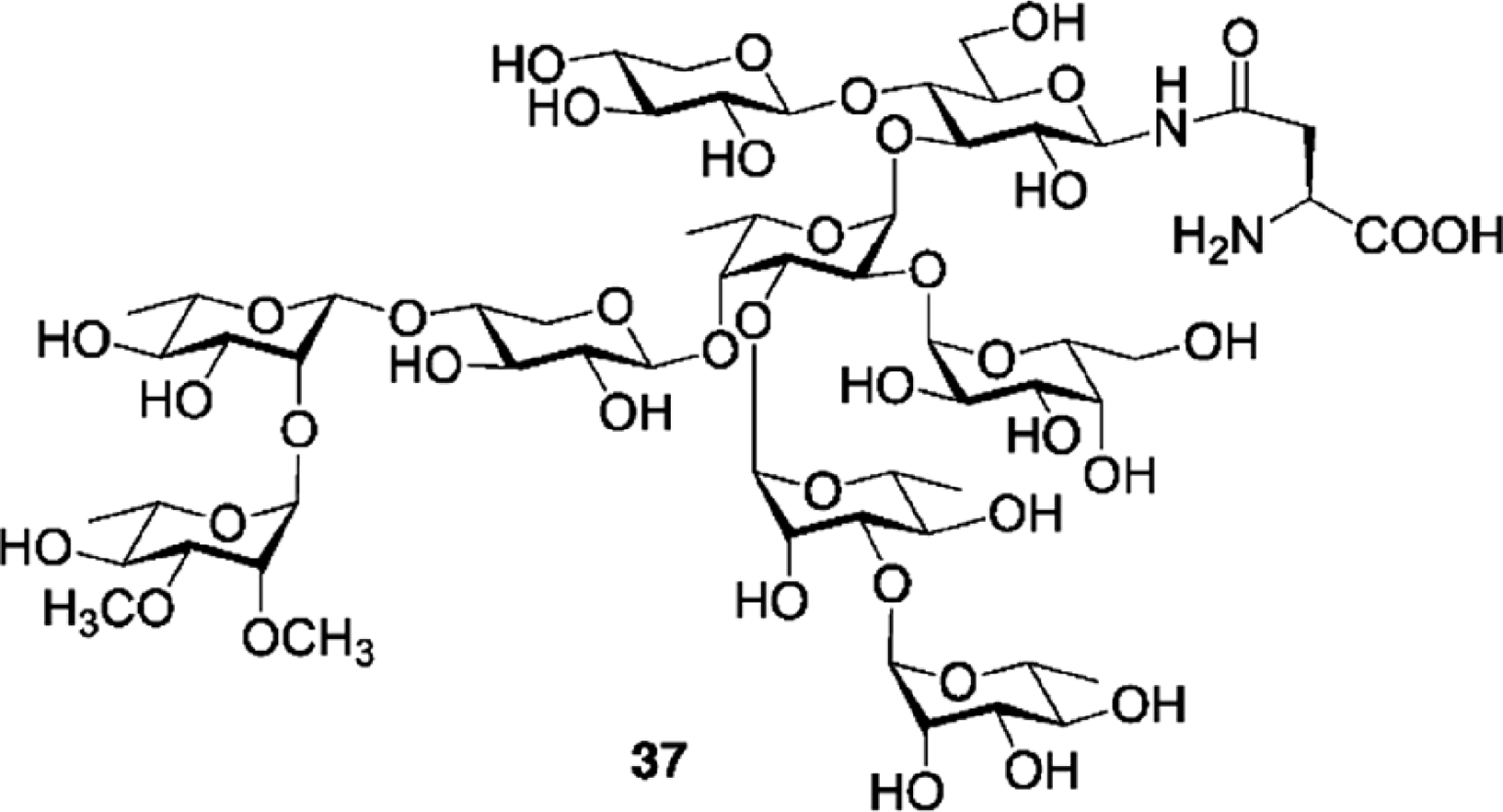
Chlorovirus PBCV-1 nonasaccharide *N*-glycan **37** that has been synthesized.^246^

The same “counterclockwise” strategy was subsequently applied to a more complex target, a nonasaccharide from virus PBCV-1 (**37**, Figure 24).^246^ A number of fragments of **37** have also been synthesized to probe the biosynthetic pathway by which these compounds are assembled by the virus.^141^

**Figure 25:**
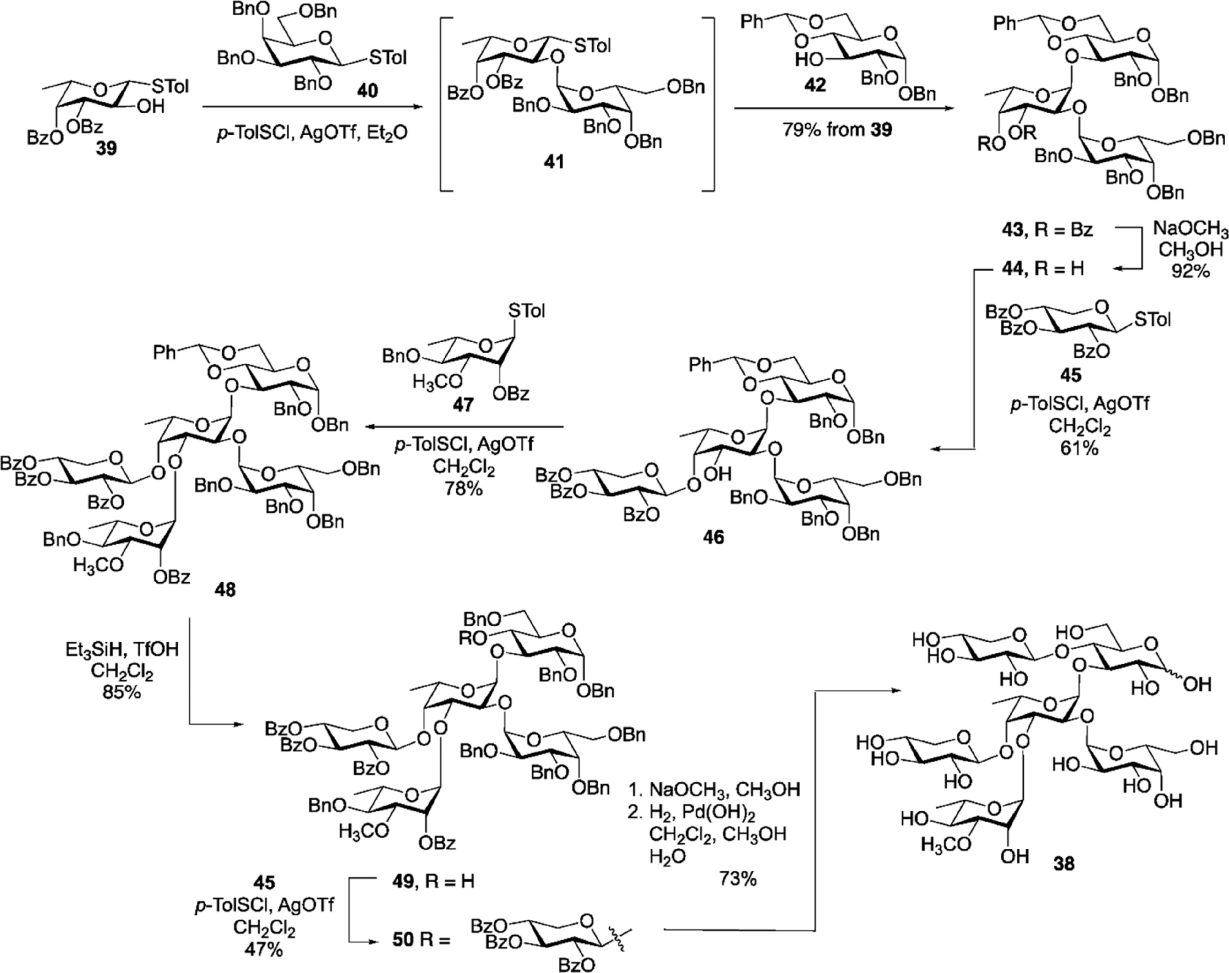
Synthesis of the chlorovirus ATCV-1 hexasaccharide **38**.^247^

Soon after the first synthesis of the chlorovirus ATCV-1 *N*-glycan was described, the synthesis of a related molecule (**38**, Figure 25) was reported by Ye and co-workers.^247^ As was the case for the synthesis of **24**, more than one approach was explored, with some being unsuccessful. The successful approach involved first the chemoselective activation of **40** in the presence of **39**, leading to the disaccharide thioglycoside **41**.^248^ Subsequent addition of the benzyl glycoside **42** to the reaction mixture produced trisaccharide **43**, in 79% overall yield. Debenzoylation of **43** proceeded in 92% yield, affording diol **44**. Interestingly, glycosylation of this diol with thioxyloside **45** led to preferential reaction at the presumably less reactive axial alcohol giving tetrasaccharide **46** in 61% yield. The authors attributed this selectivity to steric blocking of the C-3 hydroxyl group by the adjacent Gal residue. Also formed, in 15% yield was a pentasaccharide derived from the addition of two Xyl residues to **44**. The remaining hydroxyl group in **46** was then glycosylated with donor **47** producing pentasaccharide **48** in good (78%) yield. Regioselective opening of the benzylidene acetal afforded an 85% yield of alcohol **49**, which was then glycosylated in 47% yield, with **45** producing the hexasaccharide **50**. Global deprotection of **50** afforded **38** in 73% yield.

**Figure 26:**
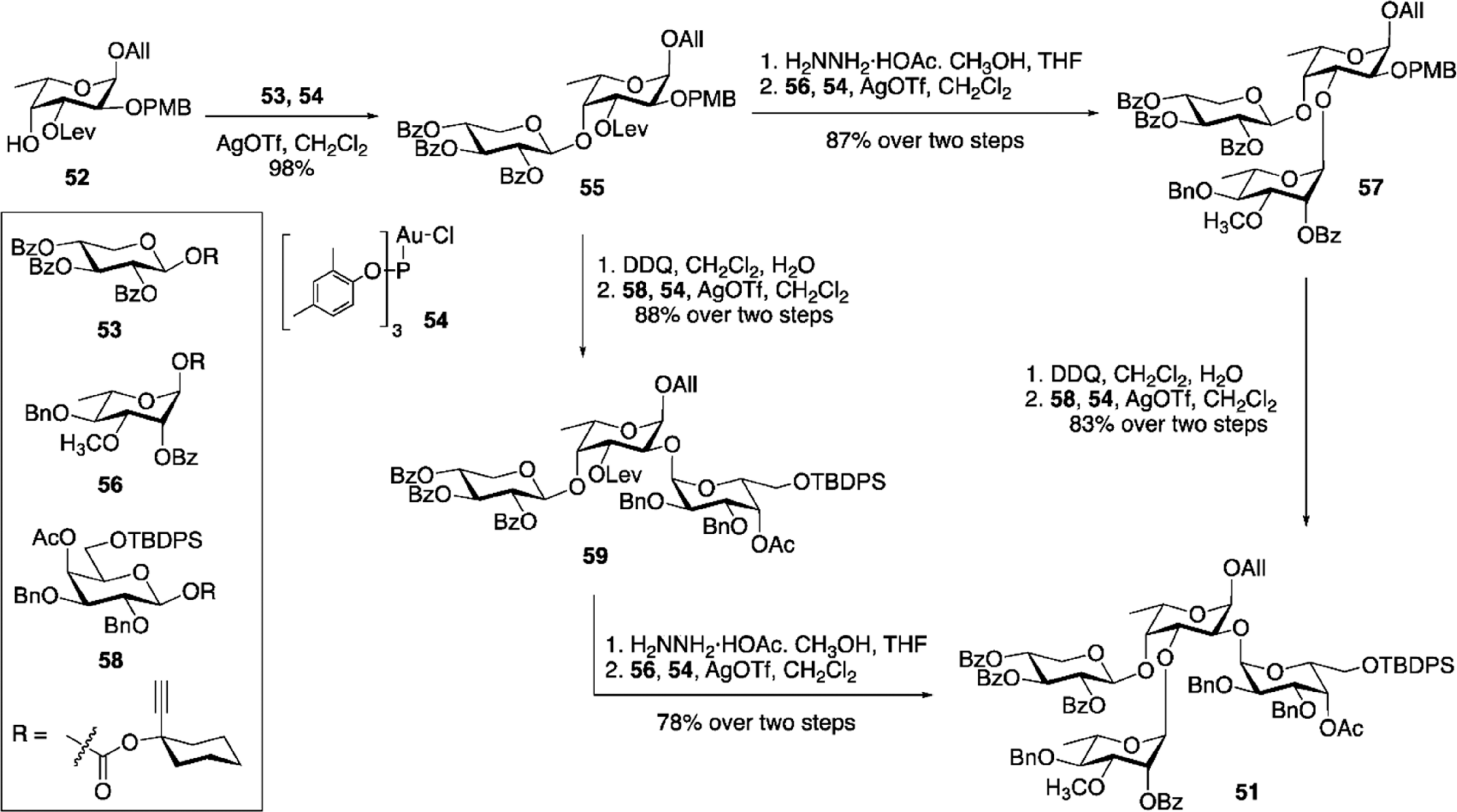
Synthesis of **51**, a protected tetrasaccharide containing the hyperbranched fucose residue in chlorovirus ATCV-1 *N*-glycans.^249^

More recently, Hotha and co-workers reported the synthesis of a fragment of the ATCV-1 *N*-glycan.^249^ The target was a tetrasaccharide containing the hyperbranched Fuc moiety (**51**, Figure 26) in protected form. The strategy made use of a gold-catalyzed glycosylation method with alkynyl-carbonate donors.^250^ Glycosylation of **52** with donor **53** in the presence of catalyst **54** and silver triflate afforded a 98% yield of **55**. Moving on from this disaccharide, two approaches were explored, both of which could successfully provide target **51**. In one route, the levulinate ester was removed from **55**, and the resulting product was glycosylated with donor **56**, a sequence that afforded trisaccharide **57** in 87% yield over the two steps. Cleavage of the *p*-methoxybenzyl ether and glycosylation of the product alcohol with donor **58** yielded tetrasaccharide **51** in 83% overall yield. Alternatively, the *p*-methoxybenzyl ether in **55** was removed and the product was glycosylated with **58**, providing an 88% yield of trisaccharide **59**. Finally, a 78% yield of tetrasaccharide **51** could be produced from **59** in two steps: removal of the levulinate ester and glycosylation of the resulting product with donor **56**.

In summary, although glycans from large viruses are a relatively recent discovery, there have been approaches developed to chemically synthesize those that have been described. A number of routes are available to prepare viosamine derivatives. With regard to the more complex chlorovirus *N*-glycans, it has been possible to assemble the key hyperbranched Fuc residue that characterizes these structures via different strategies. When the Fuc is linked to a simple aglycone, the assembly of this motif is possible using a variety of addition sequences: i.e., O-3→O-2→O-4 glycosylation,^244^ O-4→O-3→O-2 glycosylation,^249^ and O-4→O-2→O-3 glycosylation.^249^ However, when the Fuc is attached to a carbohydrate residue, greater difficulties are observed. In these latter cases, the successful strategies described have been the “counterclockwise” addition of the substituents (O-4→O-3→O-2 glycosylation) starting from a trisaccharide acceptor,^244,246^ or an O-2→O-4→O-3 glycosylation strategy producing a pentasaccharide where the proximal Xyl residue is added last.^247^ Given their unique structures, the synthesis of chlorovirus *N*-glycans will undoubtedly continue to attract interest.

**Figure 27:**
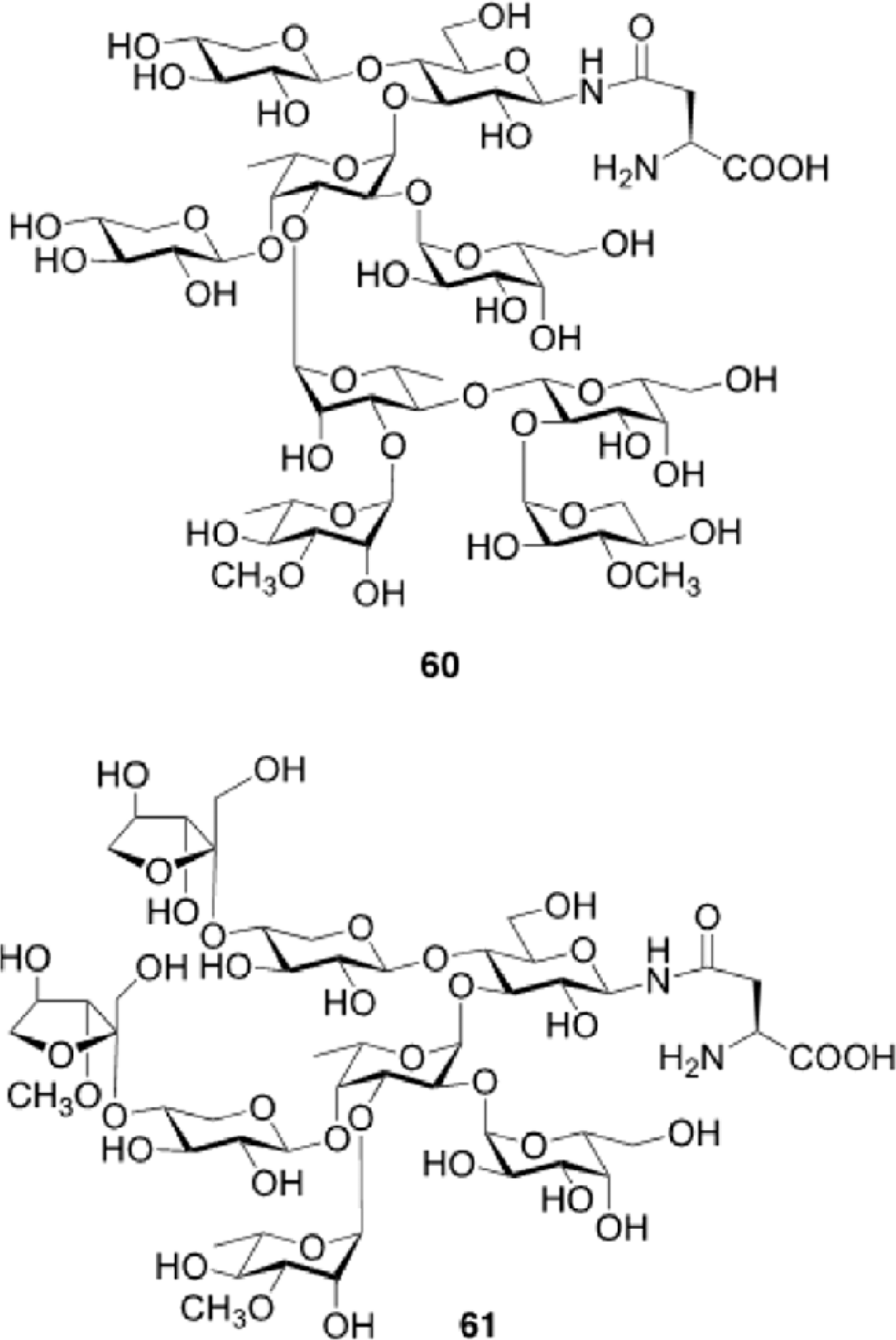
Examples of additional chlorovirus*N*-glycans that have not been synthesized.^13^ The species **60** is equivalent to the oligosaccharide **NY-2A**_**1**_ isolated from the chlorovirus NY-2A (Figure 9), **61** is common in chloroviruses TN603 and MT325, and the difference between the two viral glycans is the methylation pattern detailed in Figure 9.

Several chlorovirus *N*-glycans of greater complexity^13^ remain to be synthesized. Examples include even more highly branched structures as that produced by the chlorovirus NY-2A (**60**, Figure 27, or **NY-2A**_**1**_ in Figure 9) and those containing unusual monosaccharides, e.g., xylulofuranose in its unsubstituted or methylated forms (**61**), present in the chloroviruses MT325 and TN603 (Figure 8). Synthetic access to these molecules will require overcoming challenges in the construction of sterically congested glycans and access to usual monosaccharide residues, in addition to clarifying the absolute configuration of the xylulose unit, which is yet to be defined. Another goal should be to provide key fragments to assay the activity of viral GTs as has been done for PBCV-1 A064R-D1, the GT that forms the β-l-Rha-(1→4)-β-d-Xyl glycosidic linkage.^141,251,252^

## 5 Conclusions and Future Perspectives

The story of large and giant viruses started ∼40 years ago, and since that time these entities have continued to surprise the scientific community. Giant viruses are unusual, and their size ranges from 150 to 2000 nm (Figure 1, Table 1), and the genomes of some of them encode up to 1500 proteins. This number of proteins is just the tip of the iceberg because their annotation reveals many other surprises: a large proportion of these proteins have functions remaining to be discovered. Of the annotated proteins, many are uncommon in viruses, such as the genes involved in carbohydrate metabolism.

The past few years have seen a significant advances in the structural glycobiology of giant viruses, with experimental-based data collected primarily on two of them: PBCV-1 and APMV, belonging to *Phycodnaviridae* and *Mimiviridae* families, respectively (Table 1, section 2). The first report connecting giant viruses to sugar metabolism appeared in 1997 with the discovery that PBCV-1 encoded a functional HAS along with some of the genes necessary to produce the monosaccharides that make this polymer (section 3.3.1).^11^ The second report followed few years later and demonstrated that this same virus encoded a functional pathway for the production of GDP-α-d-Rha and GDP-β-l-Fuc (section 3.1.1.1).^148^

Since that time, reports have steadily increased. New sugar biosynthetic routes have been characterized (section 3.1), and the enzymes discovered have triggered an interest in the scientific community, as noted by the crystallographic studies that have flourished in response. Another advance has been the characterization of the glycans that decorate the capsids of the giant viruses, with the first report appearing less than a decade ago. This report detailed the structure of the *N*-glycans of PBCV-1 (section 3.2.1),^177^ and subsequently other studies have provided more information on the glycosylation patterns of chloroviruses (section 3.2.1)^177,179,192,193^ and on the fibril architecture of APMV (section 3.2.2).^65,194^

As always happens in science, every finding triggers new questions. As for PBCV-1, and chloroviruses in general, the finding of *N*-glycans that differ from those in any cellular organism opens many research avenues. For example, GTs involved in their synthesis are still being discovered. These enzymes are interesting because most are predicted to be soluble; consequently, they might be of interest for biotechnological applications.

Another intriguing question is how are these *N*-glycans added to the protein. Does this occur by a sequential addition of the sugars? Or, are they preassembled and transferred en block onto the protein? Perhaps the mechanism is a compromise of the previous two: a small core oligosaccharide is preassembled and transferred onto the capsid protein, and then the other sugars are added sequentially. Related to these questions are if any of the sugars, in particular the Glc moiety, is linked to a dolichol-like carrier. The *N*-glycosylated asparagine occurs in an atypical sequon, so that one can expect that the addition of these glycans occurs by a mechanism different from those previously identified. In support of this hypothesis is the fact that *N*-glycosylation in *Chlorella* is inhibited by tunicamycin, while the viral glycosylation continues undisturbed.

All of the points mentioned above are only a fraction of the stimuli that these viruses can trigger in the scientific community. Organic chemists have already taken up the challenge of the synthesis and structural chemistry of chlorovirus glycans, and their involvement is also key to studying the GTs involved by preparing the appropriate substrates to test.

However, other scientific areas can take this challenge by offering the tools appropriate to answer the many open questions. For instance, these viruses can be an attractive playground to develop new chemical biology probes.^253^ Such tools can offer shortcuts to trap biosynthetic intermediates or to follow the destiny of sugar analogues to learn about the capsid assembly mechanism, a fundamental step in viral replication.

Another interesting question concerns what happens to the host once infected. It is already clear that some chloroviruses modify the chlorella cell wall, which becomes covered with a thick layer of hyaluronan and/or chitin (section 3.3.1). However, nothing is known about the glycosylation pattern of the algal proteins, and this is an unexplored field of research.

All of the questions raised from chloroviruses apply, with the appropriate adaptations, to the other virus for which experimental data are available, APMV. The capsid of this virus, like almost all the others in the same family, is covered by a thick layer of fibrils, recently demonstrated to be made of proteins and polysaccharides. Notably, APMV is the first virus to be glycosylated with polysaccharides.^65^ Again, the mechanism by which these glycans are produced is unknown.

Another unique point for this virus is the involvement of collagenic proteins in the assembly of the capsid. Collagen is a molecule only present in the animal world, and APMV encodes seven collagen-like proteins, together with L230, a protein able to glycosylate the collagenic proteins by adding glucose instead of galactose (the monosaccharide present in mammalian collagen) to the hydroxylated lysine (section 3.3.3). A last intriguing point is the GlcNAc biosynthetic route that in APMV follows the eukaryotic strategy by using enzymes that are not necessarily from eukaryotes (section 3.1.2.2).

Collectively, data on PBCV-1 and APMV lead to deeper evolution questions: (i) How did these viruses acquire their glycosylation machinery? (ii) Why do they maintain it? (iii) What advantages does glycosylation confer? The answers to each of these questions and more remain to be addressed, and they just denote that the field of viral glycosylation is all but explored.

Moreover, answers to these questions may change depending on the virus considered. It should be noted that these questions were raised just by studying two viruses, PBCV-1 and APMV, while the number of giant viruses is constantly increasing, and information on their glycosylation machinery is only from in silico analyses; they are a new world to explore. For instance, one simple question already arises by considering that CroV and Fadolivirus encode Kdo biosynthetic machinery (section 3.1.2.4). This monosaccharide is a hallmark of Gram-negative bacteria, and outside this domain it is only found in plant pectin. This raises the question: Where do these two giant viruses stand from an evolutionary perspective? By extending this observation to all giant viruses, it appears that their glycosylation machinery mixes traits from the three forms of life. It should be noted that the evolutionary history of giant viruses has been proposed to predate the emergence of modern eukaryotic organisms.^16^ Maybe the suggestion in 2001 that glycosylation of the PBCV-1 major capsid protein occurred by an ancestral pathway that existed prior to ER and Golgi is not far out of line.^254^

Despite the many unanswered questions, one thing is clear: giant viruses are a new source of inspiration for chemists, biochemists, and other colleagues in life science because the understanding of their glycobiology is intriguing. These viruses provide major challenges but will offer many scientific rewards.

## Abbreviations

A5P: d-arabinose-5-phosphate
aa: amino acids
aaRS: amino-acyl tRNA synthetases
aa: amino acid
AaV: Aureococcus anaphagefferens virus
ADP: adenosine diphosphate
API: arabinose-5P isomerase
Asn: asparagine
Ara: arabinose
ATCV-1: Acanthocystis turfacea chlorovirus 1
BsV: Bodo saltans virus
CBD: cellulose binding domain
CeV: Chrysochromulina ericina virus
CHS: chitin synthase
CKS: CMP-Kdo synthase
CMP: cytidine monophosphate
CroV: Cafeteria roenbergensis virus
CTP: cytidine triphosphate
Da: Dalton
dHex: deoxyhexose
dHexN: deoxyhexosamine
dTDP-d-glucose: thymidine-5′-diphospho-α-d-glucose
DTT: dithiothreitol
*E. coli*: *Escherichia coli*
EDTA: ethylenediaminetetraacetic acid
Fuc: fucose
FucT: fucosyltransferase
Gal: galactose
GalT: galactosyltransferase
GFAT: glutamine-fructose 6-phosphate amidotransferase
GlcA: glucuronic acid
GlcNAc: *N*-acetylglucosamine
GDP: guanosine diphosphate
GMD: GDP-d-mannose-4,6-dehydratase
GMER: GDP-4-keto-6-deoxy-d-mannose epimerase/reductase
GNAT: GlcN-6P *N*-acetyltransferase
GST: glutathione S-transferase
GT: glycosyltransferase
HA: hyaluronan or hyaluronic acid
HAS: hyaluronan synthase
Hex: hexose
HexN: hexosamine
HexNAc: *N*-acetyl-hexosamine
Hys: histidine
HPLC: high pressure liquid chromatography
IPTG: isopropyl β-d-1-thiogalactopyranoside
Kdo: 3-deoxy-d-*manno*-oct-2-ulosonic acid
KdoPS: Kdo-8P synthase
KdoPase: Kdo-8P Phosphatase
Lys: lysine
Man: mannose
MBP: maltose-binding protein
MCP: major capsid protein
MW: molecular weight
NADP^+^: nicotinamide adenine dinucleotide phosphate
NCLDV(s): nucleocytoplasmic large DNA virus(es)
OG: oxoglutarate
ORF: open reading frame
PBCV-1: Paramecium bursaria chlorella virus
PBS: phosphate buffer saline
PDB: Protein Data Bank
PEG: protein-encoding genes
PEP: phosphoenolpyruvate
PFU: plaque-forming units
PI: post infection
PL: polysaccharide lyase
PLP: pyridoxal phosphate
PMSF: phenyl-methylsulfonylfluoride
PNGase: peptide-*N*-glycosidase F
PSORT: protein subcellular localization prediction tool
Qui: 6-deoxy-Glc or quinovose
QuiNAc: 2-acetamido-2,6-dideoxy-Glc or 2-acetamido-quinovose
QuiNAc4NAc: 2,4-diacetamido-2,4,6-trideoxy-Glc or 2,4-diacetamido-2,4-quinovose or di-*N*-acetyl-bacillosamine
Rha: rhamnose
2RHAMM: receptor for hyaluronan mediated motility
Ru5P: d-ribulose-5-phosphate
SAM: *S*-(5′-adenosyl)-l-methionine iodide
SDR: short-chain dehydrogenase/reductase enzyme
SDS-PAGE: sodium dodecyl sulfate–polyacrylamide gel electrophoresis
Ser: serine
TetV: tetraselmis virus
Thr: threonine
Tyr: tyrosine
UAP: UDP-GlcNAc pyrophosphorylase
UDP: uridine diphosphate
UGD: UDP-d-Glc (or GlcNAc) 4,6-dehydratase
UGDH or UDP-GlcDH: UDP-d-glucose dehydrogenase
UGER: UDP-4-keto-6-deoxy-d-glucose 3,5-epimerase/4-reductase
VF: viral factory
Xyl: xylose
XylT: xylosyltransferase
Xul: xylulose
WT: wild-type
